# Testing Two Different Doses of Tiotropium Respimat® in Cystic Fibrosis: Phase 2 Randomized Trial Results

**DOI:** 10.1371/journal.pone.0106195

**Published:** 2014-09-04

**Authors:** Judy M. Bradley, Paul Koker, Qiqi Deng, Petra Moroni-Zentgraf, Felix Ratjen, David E. Geller, J. Stuart Elborn, on behalf of the Tiotropium Cystic Fibrosis Study Group

**Affiliations:** 1 Institute of Nursing and Health Research, University of Ulster, Ulster, Northern Ireland; 2 Boehringer Ingelheim Pharmaceuticals Inc, Ridgefield, Connecticut, United States of America; 3 Boehringer Ingelheim Pharma GmbH & Co KG, Ingelheim, Germany; 4 The Hospital for Sick Children, Toronto, Ontario, Canada; 5 Florida State University College of Medicine, Orlando, Florida, United States of America; 6 Centre for Infection and Immunity, Queen’s University Belfast, Belfast, Northern Ireland; University of Modena and Reggio Emilia, Italy

## Abstract

**Background:**

Tiotropium is a once-daily, long-acting anticholinergic bronchodilator with the potential to alleviate airway obstruction in cystic fibrosis. Our objective was to evaluate the efficacy and safety of 2.5 and 5 µg once-daily tiotropium delivered via the Respimat Soft Mist Inhaler vs. placebo in people with cystic fibrosis.

**Methods:**

This phase 2, 12-week, randomized, double-blind, placebo-controlled parallel-group study of tiotropium Respimat as add-on to usual cystic fibrosis maintenance therapy included people with cystic fibrosis with pre-bronchodilator forced expiratory volume in 1 second (FEV_1_) ≥25% predicted. Co-primary efficacy end points were change from baseline in percent-predicted FEV_1_ area under the curve from 0 to 4 hours (FEV_1_ AUC_0–4h_), and trough FEV_1_ at the end of week 12.

**Findings:**

A total of 510 subjects with cystic fibrosis aged 5–69 years were randomized. Both doses of tiotropium resulted in significant improvement compared with placebo in the co-primary efficacy end points at the end of week 12 (change from baseline in percent-predicted FEV_1_ AUC_0–4h_: 2.5 µg: 2.94%, 95% confidence interval 1.19–4.70, p = 0.001; 5 µg: 3.39%, 95% confidence interval 1.67–5.12, p = 0.0001; in percent-predicted trough FEV_1_∶2.5 µg: 2.24%, p = 0.2; 5 µg: 2.22%, p = 0.02). There was a greater benefit with tiotropium 5 vs. 2.5 µg. No treatment-related adverse events or unexpected safety findings were observed in patients taking tiotropium.

**Conclusions:**

Tiotropium significantly improved lung function in people with cystic fibrosis. The improvement was greater with the higher dose than the lower dose, with no difference in adverse events.

**Trial Registration:**

ClinicalTrials.gov NCT00737100 EudraCT 2008-001156-43.

## Introduction

Respiratory disease is the major cause of death in people with cystic fibrosis (CF), and new therapies addressing important pathophysiologic aspects of CF are urgently needed [1]. Treatments targeting airflow obstruction may provide clinical benefit to people with CF. Current standard therapy includes antibiotics, airway clearance techniques, dornase alfa, hypertonic saline, and inhaled bronchodilators [2]. Approximately 80% of people with CF use short- and/or long-acting bronchodilators, although their place as long-term therapy is not clearly defined and there are no bronchodilators with an approved indication for CF [3], [4].

Bronchodilator agents improve pulmonary function and lessen wheezing in many people with CF [4]. The similar effectiveness of β_2_ agonists and anticholinergics suggests that a significant proportion of airflow obstruction in CF is parasympathetically mediated [5]. Anticholinergic treatment may therefore prove a valuable add-on to current CF treatments.

A single dose of ipratropium bromide, a short-acting anticholinergic compound, has demonstrated lung function improvements in people with CF [6]. However, the long-term effects of maintenance therapy with ipratropium bromide have not been studied in CF. Tiotropium bromide (hereafter referred to as tiotropium), a long-acting anticholinergic compound delivered as an aqueous solution using the Respimat Soft Mist Inhaler, has consistently shown superior efficacy to ipratropium [7], and is well tolerated in the treatment of chronic obstructive pulmonary disease (COPD); these findings are consistent with those observed with the tiotropium 18 µg HandiHaler [8]. Tiotropium Respimat therefore has the potential to alleviate airflow obstruction in people with CF. Tiotropium Respimat was selected because the inhaler and formulation are suitable for populations of all ages. The inhaler is an active device that releases drug substance as an aerosol; hence, no minimal inspiratory flow is required by the patient. Suitability of the Respimat Soft Mist Inhaler was demonstrated in children aged <5 years using Respimat with a valved holding chamber and facemask [9], [10]. Results from a phase 1 study *(manuscript in press)* showed that safety and tolerability of tiotropium are acceptable when compared with placebo in people with CF. Based on available data, tiotropium delivered by Respimat was tested at doses of 2.5 and 5 µg in this phase 2 study.

The primary objective was to evaluate the efficacy and safety of 12 weeks’ treatment with tiotropium Respimat 2.5 or 5 µg once daily compared with placebo in people with CF. We hypothesized that treatment with tiotropium for 12 weeks is more effective in improving lung function compared with placebo, and that the higher dose of tiotropium (5 µg) is more effective than the 2.5 µg dose.

## Methods

The protocol for this trial and supporting CONSORT checklist are available as supporting information; see Checklist S1 and Protocol S1.

### Study Design

This was a double-blind, placebo-controlled, multicenter, multinational, parallel-group study to evaluate treatment with two doses of tiotropium (2.5 and 5 µg once daily, two inhalations at the same time of day) compared with placebo over 12 weeks (ClinicalTrials.gov identifier: NCT00737100). The trial period was from 23 September 2008 to 2 April 2010. The study was conducted at 94 centers in Australia, Europe, Canada, and the United States.

Ethical review was undertaken for each site/country by the local institutional review board/independent ethics committee and approval granted by the Competent Authority in each state.

### Participants

Participants with documented CF had to be able to perform spirometric maneuvers according to American Thoracic Society (ATS) standards [11]. Patients of all ages were included, except in Russia, where patients aged <6 years were excluded. Subjects had to have a screening pre-bronchodilator forced expiratory volume in 1 second (FEV_1_) ≥25% of predicted values [12], [13] and inhale medication competently using the Respimat Soft Mist Inhaler. Patients were trained using a placebo Respimat device. They were to be clinically stable as defined by no evidence of acute respiratory tract infection or pulmonary exacerbation requiring use of antibiotics or oral corticosteroids within 4 weeks of screening. Pre-bronchodilator FEV_1_ at week 0 or start of treatment had to be within 15% of FEV_1_ at screening. All patients continued to receive their standard-of-care treatment during the study; tiotropium and placebo were considered add-on to usual therapy. If patients were diagnosed with a pulmonary exacerbation during the study period then this was recorded as an adverse event (AE) or serious AE, but there was no requirement to discontinue medication, and this decision was determined by the patient and physician. If taking long-term medication, participants had to agree to continue it throughout the study. Patients were allowed to continue using short-acting β-agonists (SABAs) and long-acting β-agonists (LABAs) if they were stabilized at least 4 weeks prior to randomization and throughout the study. Furthermore, patients taking SABAs and LABAs were required to have a 6-hour or 12-hour washout period, respectively, prior to all spirometry measurements.

Daily inhaled antibiotic use was allowed if stabilized for at least 6 weeks prior to and throughout the study period. Cyclic therapies (e.g., 4-week on/off tobramycin inhalation solution [TIS]) can impact treatment outcomes in short-term early phase trials. Cycled inhaled antibiotic use (e.g., TIS every other month) was allowed; however, the start of treatment had to be scheduled 2 weeks after the most recent TIS cycle and the last TIS cycle was to happen 2 weeks before the last treatment visit. This trial’s 12-week design minimized the impact of cyclic medications by ensuring that baseline and final assessments were scheduled at a similar time point in the TIS cycle (i.e., both 2 weeks following completion of a TIS cycle). In order to comply with a 4-week on/off TIS regimen, the end-of-treatment visit may have been scheduled within 10–14 weeks after start of treatment to ensure baseline and end of study assessments occurred at mid-point of an off month in participants cycling TIS on alternate months. Physicians were advised to follow similar timing for other cycled medication use as for TIS, although this was not an official protocol procedure. Written informed consent was obtained from each patient or the patient’s legal representative. Exclusion criteria (see Protocol S1 and File S1) included previous intolerance to tiotropium.

### End Points

The co-primary end points for this trial were: i) the change from baseline (30 min prior to administration of first tiotropium dose at randomization; week 0) in percent-predicted FEV_1_ area under the curve from time 0–4 hours (AUC_0–4h_) at the end of week 12; and ii) the change from baseline (30 min prior to administration of first tiotropium dose at randomization; week 0) in percent-predicted trough FEV_1_ at the end of week 12. The secondary outcomes in this study were changes from baseline in forced vital capacity (FVC), residual volume/total lung capacity (RV/TLC), forced expiratory flow (FEF) from 25% to 75% of vital capacity (FEF_25–75_), Respiratory and Systemic Symptoms Questionnaire (RSSQ), and health-related quality of life (Cystic Fibrosis Questionnaire-Revised [CFQ-R]) at the end of week 12 [14]. AEs were also recorded. To measure exposure to the study drug, all patients were asked to return all dispensed Respimat inhalers.

### Assessments

#### Pulmonary function tests (PFTs)

The use of spirometers, including daily calibration, met ATS/European Respiratory Society criteria [15]. The qualifying PFTs (FEV_1_ and FVC) were conducted at the screening visit. At weeks 0 and 12, PFTs were performed pre-dose (–10 minutes prior to study drug inhalation), at 30 minutes, and at 1, 2, 3, and 4 hours after inhalation of study drug. FEV_1_ AUC_0–4h_ was calculated using the trapezoidal rule [16], divided by the duration (4 hours) reported in liters. Trough FEV_1_ was defined as the FEV_1_ value performed at –30 minutes prior to study drug inhalation. At weeks 4 and 8, PFTs were performed 30 minutes prior to drug administration. During the follow-up visit, after 30 days post-treatment (or at the time of premature study discontinuation), a single PFT was performed. Percent-predicted FEV_1_ was calculated using the reference equations by Wang and colleagues [12] for pediatric/adolescents (aged 6–18 years) and by Knudson and colleagues [13] for adults (aged >18 years). RV/TLC measurements were performed as described in File S1.

#### Patient-reported outcomes

The proportion of patients with at least one pulmonary exacerbation during the double-blind period was analyzed using the RSSQ [17], [18]. Health-related quality of life was assessed as the change from baseline in CFQ-R score [14]. Both methods are described in detail in File S1. For patients aged 6–13 years and depending on the patient’s age and reading ability, the CFQ-R was administered to the patient directly, indirectly, or administered to their parent or caregiver. No patient under the age of 6 years completed the CFQ-R.

#### Safety

Medical history was recorded at screening. All patients were reviewed at screening and baseline entry into the study and at 2, 4, 8, and 12 weeks of therapy, and then at 30 days’ post-treatment. Health status of patients who withdrew prematurely from the treatment period was followed up until their predicted study completion date. At each visit, all AEs and serious AEs, regardless of causality, were recorded.

### Statistical Analyses

The primary analyses were performed in all randomized patients who received at least one dose of study drug and had both baseline and at least one post-dose PFT measurement at or before 12 weeks for either co-primary efficacy variable. Mean change from baseline in the co-primary efficacy variables (FEV_1_ AUC_0–4h_ and trough FEV_1_) was analyzed using a restricted maximum likelihood–based mixed-effect model with repeated measures (MMRM). To model the within-patient errors, an unstructured covariance matrix was used. Analyses were implemented using Statistical Analysis System software version 8.2 (SAS Institute Inc., Cary, NC, USA). The primary comparisons were between treatments after 12 weeks. The first co-primary end point, FEV_1_ AUC_0–4h_, was also stratified by age group. The age variable was dichotomized into patients aged 12 years and older and patients aged 11 years and younger, and was included in the model as a fixed effect. Other terms in the model were “treatment,” “visit,” “treatment-by-visit interaction,” “baseline” and “baseline-by-visit interaction,” and “random effect of center.” Prespecified subgroup analyses for both primary end points included age group and baseline LABA use. The subgroup analyses were performed by adding subgroup, treatment-by subgroup, visit-by-subgroup, and treatment-by-visit-by subgroup interaction into the primary model.

All secondary end points on additional PFT parameters were analyzed as in the primary analysis, using the same MMRM model. For exacerbation analyses, Mantel-Haenszel test was adjusted for age group; treatment and age group were covariates for logistic regression analysis. The duration of pulmonary exacerbation was calculated based on the AE listings. Safety end points were summarized descriptively.

Data considered to be missing at random were not imputed, but handled in the analysis via the use of the MMRM model; only data that could be considered not missing at random were imputed. Randomly missing data with no subsequent non-missing values for that visit were imputed using the last observation carried forward technique (FEV_1_, FVC, and FEF_25–75_ measurements).

In order to detect a difference of five units in the percentage change from baseline in FEV_1_ values between tiotropium and placebo with 80% power at a two-sided significance level of 5%, a minimum of 465 patients in the full analysis set were required (155 in each treatment group). Based on a previous study [19], an approximate estimate of the expected standard deviation for FEV_1_ percent-predicted value at screening was used (15.66). In this study, the ratio of adult to pediatric patients was approximately 2∶1.

A detailed description of blinding and randomization and handling of missing data can be found in File S1.

## Results

**Figure 1:**
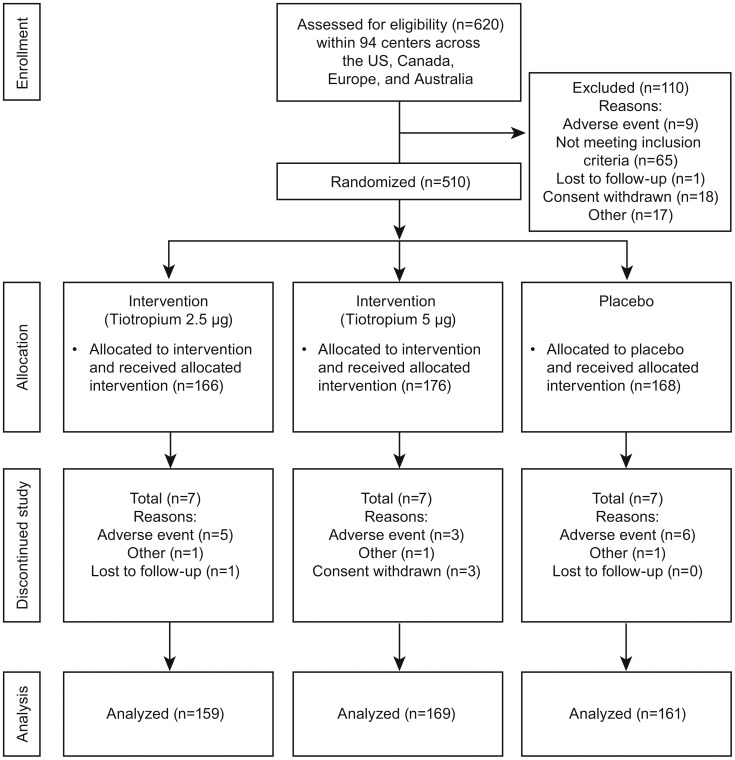
Flow diagram of participant recruitment and randomization [20].

The study flow for this trial is summarized in Figure 1 [20].

### Demographics and Other Baseline Characteristics

Of 620 recruited patients, a total of 510 patients (54% male) were randomized and treated (placebo, n = 168; tiotropium 2.5 µg, n = 166; tiotropium 5 µg, n = 176). Mean (SD) age was 20.9 (11.6) years (range, 5–69 years) and 27.1% were aged ≤11 years (range, 5–11 years). There were no clinically relevant differences between treatment groups (overall or by age group) in any demographic or baseline characteristic (Table 1).

**Table 1:** Patient demographics and characteristics of the study population at baseline.

	Placebo	Tiotropium 2.5 µg	Tiotropium 5 µg	Total
No. of patients	168	166	176	510
Male sex, n (%)	96 (57.1)	85 (51.2)	94 (53.4)	275 (53.9)
Age, years (mean, SD)	20.4 (11.6)	21.5 (12.0)	20.7 (11.3)	20.9 (11.6)
Age group, n (%)				
≤11 years	44 (26.2)	42 (25.3)	52 (29.5)	138 (27.1)
≥12 years	124 (73.8)	124 (74.7)	124 (70.5)	372 (72.9)
Percent-predicted FEV_1_, (mean, SD)	76.6 (23.8)	75.4 (26.7)	75.9 (22.6)	76.0 (24.3)
≤11 years	94.9 (16.7)	98.8 (16.1)	90.3 (15.5)	94.3 (16.3)
≥12 years	70.1 (22.5)	67.5 (24.9)	69.9 (22.4)	69.2 (23.3)
BMI, kg/m^2^ (mean, SD)	20.3 (4.4)	19.9 (4.0)	20.0 (4.1)	20.1 (4.2)
Baseline* concomitant pulmonary medications, n (%)	129 (76.8)	121 (72.9)	128 (72.7)	378 (74.1)
SABA	103 (61.3)	90 (54.2)	111 (63.1)	304 (59.6)
LABA	67 (39.9)	63 (38.0)	58 (33.0)	188 (36.9)

*Baseline includes all medications used on at least 1 day between informed consent and randomization (inclusive) and on at least 1 day between randomization and the first day of randomized drug intake (inclusive). BMI, body mass index; FEV_1_, forced expiratory volume in 1 second; LABA, long-acting β-agonist; SABA, short-acting β-agonist; SD, standard deviation.

### Co-primary Efficacy End Points

**Figure 2:**
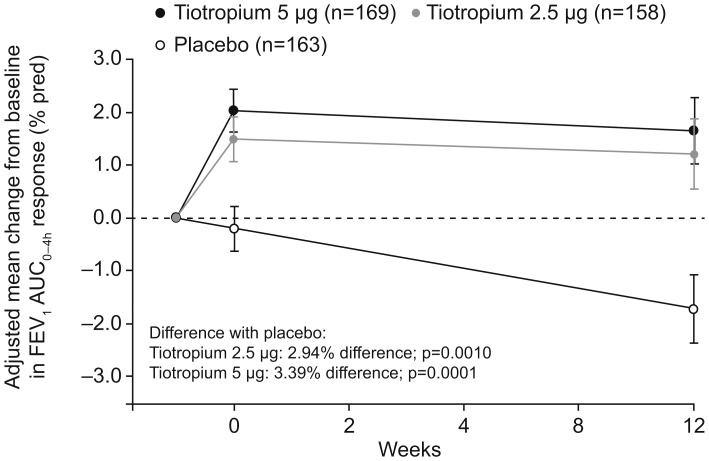
Adjusted mean FEV_1_ AUC_0–4h_ (percent-predicted ± SE) change from baseline (full analysis set). AUC_0–4h_, area under the curve from 0 to 4 hours; FEV_1_, forced expiratory volume in 1 second.

For percent-predicted FEV_1_ AUC_0–4h_ at the end of week 12, adjusted changes (improvements) from baseline were greater for both doses of tiotropium compared with placebo (tiotropium 2.5 µg: 1.20%; tiotropium 5 µg: 1.65%; placebo: –1.74%. Tiotropium 2.5 µg difference from placebo [95% CI]: 2.94% [1.19, 4.70], p = 0.001; tiotropium 5 µg difference from placebo [95% CI]: 3.39% [1.67, 5.12], p = 0.0001) (Fig. 2). The adjusted mean change was larger in the tiotropium 5 µg group than in the tiotropium 2.5 µg group (Fig. 2). The additional bronchodilator efficacy observed in the tiotropium 5 µg group was driven by the results obtained in patients younger than 12 years (Table 2). Both tiotropium dose groups had estimated mean changes (improvements), measured in liters at the end of week 12 (Table 2), that were statistically significantly greater than that of placebo.

**Table 2:** Adjusted mean (SE) changes from baseline in FEV_1_ AUC_0–4h_ overall and in patients (aged ≤11 years and ≥12 years) treated with tiotropium (2.5 or 5 µg) or placebo after 12 weeks*.

Age group	Treatment	Difference from placebo		
Treatment	Mean (SE)	Mean (SE) p value		95% CI
**Overall, % predicted**				
Placebo (n = 163)	–1.74 (0.65)			
Tiotropium 2.5 µg (n = 158)	1.20 (0.66)	2.94 (0.89)	0.0010	1.19–4.70
Tiotropium 5 µg (n = 169)	1.65 (0.63)	3.39 (0.88)	0.0001	1.67–5.12
**Overall, L**				
Placebo (n = 163)	−0.07 (0.02)			
Tiotropium 2.5 µg (n = 158)	0.02 (0.02)	0.09 (0.03)	0.0004	0.04–0.14
Tiotropium 5 µg (n = 169)	0.03 (0.02)	0.09 (0.02)	0.0002	0.05–0.14
**≤11 years, % predicted**				
Placebo (n = 43)	–0.32 (1.26)			
Tiotropium 2.5 µg (n = 42)	2.01 (1.29)	2.33 (1.74)	0.1801	–1.08 to 5.75
Tiotropium 5 µg (n = 50)	3.57 (1.16)	3.89 (1.67)	0.0199	0.62–7.17
**≥12 years, % predicted**				
Placebo (n = 120)	–2.55 (0.75)			
Tiotropium 2.5 µg (n = 116)	0.57 (0.76)	3.12 (1.04)	0.0028	1.08–5.17
Tiotropium 5 µg (n = 119)	0.55 (0.75)	3.11 (1.03)	0.0028	1.08–5.14

*Analysis of the full analysis set study group based on mixed-effect model with repeated measures model using unstructured covariance matrix.

AUC_0–4h_, area under the curve from 0 to 4 hours; CI, confidence interval; FEV_1_, forced expiratory volume in 1 second; SE, standard error.

Adjusted changes (improvements) from baseline in percent-predicted trough FEV_1_ (at end of week 12) were greater for both doses of tiotropium compared with placebo (tiotropium 2.5 µg: 0.81%; tiotropium 5 µg: 0.78%; placebo: –1.44%. Tiotropium 2.5 µg difference from placebo [95% CI]: 2.24% [0.38, 4.11], p = 0.02; tiotropium 5 µg difference from placebo [95% CI]: 2.22% [0.38, 4.06], p = 0.02; Table 3). The mean adjusted change was similar between the tiotropium 2.5 µg and 5 µg groups and was not affected by age.

**Table 3:** Adjusted mean (SE) changes from baseline in overall trough FEV_1_ response and in patients (aged ≤11 years and ≥12 years) treated with tiotropium (2.5 or 5 µg) compared with placebo after 12 weeks*.

	Treatment	Difference from placebo		
Treatment	Mean change (SE)	Mean change (SE) p value		95% CI
**Overall, % predicted**				
Placebo (n = 163)	–1.44 (0.71)			
Tiotropium 2.5 µg (n = 158)	0.81 (0.71)	2.24 (0.95)	0.0184	0.38–4.11
Tiotropium 5 µg (n = 169)	0.78 (0.69)	2.22 (0.93)	0.0179	0.38–4.06
**Overall, L**				
Placebo (n = 163)	−0.06 (0.02)			
Tiotropium 2.5 µg (n = 158)	−0.00 (0.02)	0.06 (0.03)	0.0330	0.00–0.11
Tiotropium 5 µg (n = 169)	−0.00 (0.02)	0.06 (0.03)	0.0281	0.01–0.11
**≤11 years, % predicted**				
Placebo (n = 43)	−0.83 (1.35)			
Tiotropium 2.5 µg (n = 42)	2.71 (1.38)	3.54 (1.86)	0.0577	−0.12 to 7.19
Tiotropium 5 µg (n = 50)	1.85 (1.24)	2.68 (1.78)	0.1322	−0.81 to 6.18
**≥12 years, % predicted**				
Placebo (n = 120)	−2.14 (0.80)			
Tiotropium 2.5 µg (n = 116)	−0.38 (0.81)	1.76 (1.11)	0.1128	−0.42 to 3.94
Tiotropium 5 µg (n = 119)	−0.11 (0.80)	2.03 (1.10)	0.0668	−0.14 to 4.20

*Analysis of the full analysis set study group based on mixed-effect model with repeated measures model using unstructured covariance matrix.

CI, confidence interval; FEV_1_, forced expiratory volume in 1 second; SE, standard error.

### Other Lung Function Measures

Both the 2.5 and 5 µg doses of tiotropium had adjusted mean improvements from baseline in percent-predicted FVC AUC_0–4h_ response that were greater than placebo (tiotropium 2.5 µg: 0.53%; tiotropium 5 µg: 1.81%; placebo: –1.30%. Tiotropium 2.5 µg difference from placebo [95% CI]: 1.83% [–0.19, 3.86], p = 0.08; tiotropium 5 µg difference from placebo [95% CI]: 3.12% [1.12, 5.12], p = 0.002). The mean adjusted change was greater for tiotropium 5 than 2.5 µg and was only statistically significant for the tiotropium 5 µg group. The observed improvement for FVC AUC_0–4h_ in the tiotropium 5 µg group was largely driven by results in patients aged ≤11 years (described in detail in File S1).

Both doses of tiotropium had estimated adjusted mean improvements in percent-predicted trough FVC response that were greater than placebo, but not statistically significant (tiotropium 2.5 µg: 0.47%; tiotropium 5 µg: 0.81%; placebo: –0.39%. Tiotropium 2.5 µg difference from placebo [95% CI]: 0.85% [–1.08, 2.79], p = 0.4; tiotropium 5 µg difference from placebo [95% CI]: 1.19% [–0.72, 3.11], p = 0.2). FEF_25–75_ results were consistent with these results; static lung hyperinflation as measured by RV/TLC showed no difference between dose groups (described in detail in File S1).

### Exposure

Overall exposure to study drug was acceptable, with a mean (SD) duration of treatment of 85.6 days (12.1); 87.5% of patients (446/510) took their study drug between >10 and ≤14 weeks. Approximately 9% of patients (47/510) took their study drug for ≥14 weeks.

### Pulmonary Exacerbation as Assessed by RSSQ and Health-Related Quality of Life by CFQ-R

The proportions of patients who had at least one pulmonary exacerbation as determined by the RSSQ and intravenous antibiotic use were lower in the tiotropium 2.5 µg (7.8%) and 5 µg (6.9%) groups compared with the placebo group (9.6%; not statistically significant; p = 0.9 and p = 0.6 vs. placebo for tiotropium 2.5 µg and 5 µg respectively). Except for one pulmonary exacerbation in each treatment group, all events occurred in patients aged ≥12 years. The CFQ-R scores did not differ from baseline throughout the study period and no difference between the treatment groups was observed (additional results detailed in File S1).

### Subgroup Analysis

Differences in response to tiotropium by baseline LABA were explored by a prespecified subgroup analysis. In patients who used LABA at baseline, a larger difference compared with placebo (n = 64) was observed for the tiotropium 5 µg group (n = 56; 5.30% difference; 3.09% vs. –2.21%; 95% CI, 2.44–8.2; p = 0.0003) than for the tiotropium 2.5 µg (n = 60; 1.79% difference; –0.43% vs. –2.21%; 95% CI, –1.03 to –4.61; p = 0.2). In patients who did not use LABA at baseline, similar differences compared with placebo (n = 99) were observed for both tiotropium groups (tiotropium 2.5 µg: n = 98, 3.63% difference, 2.19% vs. –1.44%, 95% CI, 1.40–5.86, p = 0.002; tiotropium 5 µg: n = 113, 2.37% difference, 0.93% vs. –1.44%, 95% CI, 0.22–4.53, p = 0.03).

### Adverse Events

The majority of patients (82.9%) reported one or more AE during the study. Most were mild to moderate in intensity and were primarily of respiratory nature; the most common AEs (defined as AE with an incidence >5% in any treatment group) were cough, exacerbation of CF, pyrexia, nasopharyngitis, and headache (Table 4). All deaths (two in the placebo group and one in the tiotropium 2.5 µg group) were due to exacerbation and/or complications of CF. An overall summary of AEs is provided in Table 5 (and in File S1). Frequency of AEs was similar in all treatment arms and there was no evidence of a treatment relationship for any AE category; small differences between treatment groups were considered consequent to variability related to the overall small number of events.

**Table 4:** Summary of adverse events that occurred in >5% of patients in any treatment group (treated set).

	Placebo	Tiotropium 2.5 µg	Tiotropium 5 µg	Total
No. of patients, n	168	166	176	510
Cough, n (%)	34 (20.2)	35 (21.1)	46 (26.1)	115 (22.5)
Cystic fibrosis*, n (%)	17 (10.1)	23 (13.9)	25 (14.2)	65 (12.7)
Pyrexia, n (%)	17 (10.1)	9 (5.4)	18 (10.2)	44 (8.6)
Nasopharyngitis, n (%)	14 (8.3)	11 (6.6)	14 (8.0)	39 (7.6)
Headache, n (%)	18 (10.7)	7 (4.2)	14 (8.0)	39 (7.6)
Sputum increased	8 (4.8)	12 (7.2)	13 (7.4)	33 (6.5)
Abdominal pain	10 (6.0)	13 (7.8)	9 (5.1)	32 (6.3)
Hemoptysis	7 (4.2)	13 (7.8)	12 (6.8)	32 (6.3)
Oropharyngeal pain	13 (7.7)	5 (3.0)	11 (6.3)	29 (5.7)
Bronchitis	9 (5.4)	6 (3.6)	10 (5.7)	25 (4.9)
Upper respiratory tract infection	6 (3.6)	8 (4.8)	11 (6.3)	25 (4.9)
Rhinorrhea	9 (5.4)	6 (3.6)	9 (5.1)	24 (4.7)
Dyspnea	9 (5.4)	8 (4.8)	6 (3.4)	23 (4.5)
Nasal congestion	4 (2.4)	9 (5.4)	10 (5.7)	23 (4.5)
Sinusitis	6 (3.6)	3 (1.8)	9 (5.1)	18 (3.5)
Arthralgia	9 (5.4)	5 (3.0)	4 (2.3)	18 (3.5)

*The preferred adverse event term used for “CF exacerbation” was “cystic fibrosis.”

A patient may have been counted in more than one preferred term. Percentages were calculated using the total number of patients per treatment as the denominator. Medical Dictionary for Regulatory Activities (MedDRA) version used for reporting: 13.0.

**Table 5:** Overall summary of adverse events (treated set).

	Placebo	Tiotropium 2.5 µg	Tiotropium 5 µg	Total
No. of patients, n	168	166	176	510
Patients with any AE, n (%)	139 (82.7)	139 (83.7)	145 (82.4)	423 (82.9)
Patients with an SAE, n (%)	7 (4.2)	13 (7.8)	10 (5.7)	30 (5.9)
Patients with a study drug-related AE*, n (%)	14 (8.3)	15 (9.0)	18 (10.2)	47 (9.2)
Patients with other significant AE^†^, n (%)	3 (1.8)	2 (1.2)	3 (1.7)	8 (1.6)
Patients with AE leading to discontinuation of study drug, n (%)	6 (3.6)	6 (3.6)	3 (1.7)	15 (2.9)
Patients with SAEs, n (%)	21 (12.5)	28 (16.9)	21 (11·9)	70 (13.7)
Fatal, n	2	1	0	3
Immediately life-threatening, n	1	0	0	1
Disability/incapacity, n	0	0	1	1
Required hospitalization, n	21	28	21	70
Prolonged hospitalization, n	1	1	0	2
Congenital anomaly, n	0	0	0	0
Other, n	0	0	0	0

*As assessed by the study investigators. ^†^As defined by International Conference on Harmonisation (ICH) E3 guidelines.

A patient may have been counted in more than one seriousness criterion. AE, adverse event; SAE, serious AE.

## Discussion

This is the first study to evaluate and provide evidence for the safety and efficacy of tiotropium in people with CF. Both doses of tiotropium resulted in statistically significant improvements compared with placebo in the co-primary efficacy end points of FEV_1_ AUC_0–4h_ and trough FEV_1_. Similarly, results for FEV_1_ AUC_0–4h_ in liters and for FVC support the findings of the primary end points. Interestingly, a treatment benefit for respiratory symptoms (based on RSSQ) or health-related quality of life (based on CFQ-R) was not observed with either dose of tiotropium. Both doses of tiotropium were safe and well tolerated. The age subgroup analyses performed in our study demonstrate that tiotropium 5 µg is effective, with a favorable safety profile in children as well as in adults. The results from this study led to a phase 3 trial being performed using the 5 µg tiotropium dose [21].

This study has a number of challenges in design, relating in particular to the use of cycled inhaled antibiotics. Study design was such that measurements were made on the off phase of inhaled antibiotic treatment for those on such treatment. The study was not sufficiently long to confidently determine the presence of any significant changes in symptoms or pulmonary exacerbations. The effects seen in the study should therefore be interpreted as demonstrating short- to medium-term effect of bronchodilator therapy by tiotropium. The study was, however, sufficiently powered to detect a meaningful change in FEV_1_.

Despite widespread use, there is limited evidence supporting the use of bronchodilators in CF. A recent systematic review concluded that short-acting anticholinergics had no consistent effect on lung function tests in people with CF [22]. Efficacy of the LABA salmeterol in people with CF has only been investigated in several short-term (<1 week duration) clinical trials [23], [24]. Results from one trial of longer-term use (24 weeks) showed improvement in FEV_1_ in people with CF compared with treatment with salbutamol; however, no significant effect of salmeterol on other spirometric indices were detected in this trial, and therefore the clinical relevance of these results is debatable [25], [22].

The highest tiotropium dose tested in this study, 5 µg daily, resulted in lung function improvement that was numerically above that observed for 2.5 µg. The study was not powered for differences between doses and no statistical comparison was performed; however, the effect of the 5 µg dose was about 15% greater than that of the 2.5 µg dose (percent-predicted FEV_1_ AUC_0–4h_) and the effect of the higher dose was more consistent across visits. Based on a numerically superior efficacy and a similar AE profile of both doses, 5 µg appeared to be the preferred dose for subsequent clinical development of tiotropium in people with CF. Longer term studies are required to evaluate the effect of tiotropium on symptoms.

Tiotropium Respimat 5 µg is also the approved dose for COPD patients, in whom evidence for improvements in lung function, exercise tolerance, and health-related quality of life, as well as reduction in the frequency of exacerbations, exists [26], [27], [28].

Effect sizes of lung function improvements for treatments used in CF patients vary widely [22] and it is difficult to compare the magnitude of improvement between different inhaled therapies such as mannitol [29], [30], hypertonic saline [31], and TIS [32], as different variables are used (absolute change in FEV_1_ from baseline vs. relative change). Currently, people with CF are recommended complex, time-intensive daily therapies that are often difficult to sustain over the long term, and the addition of new therapies usually increases that burden [33]. Drug delivery using the Respimat Soft Mist Inhaler is fast, easier than the more traditional metered-dose inhaler, and does not require a power source [34]. Furthermore, because it is effective over 24 hours, tiotropium only requires once-daily inhalation and therefore may ease the burden of the number of daily medications required in CF, contributing to improved compliance.

## Conclusion

This study provides evidence of the efficacy and safety of inhaled tiotropium delivered by the Respimat Soft Mist Inhaler in people with CF and supports proceeding to further investigations in phase 3.

## Supporting Information

File S1 **Supporting information file.**
(DOCX)

Protocol S1 **Trial Protocol.**
(PDF)

Checklist S1 **CONSORT checklist.**
(DOC)
