# Supplementary material for: Testing Two Different Doses of Tiotropium Respimat® in Cystic Fibrosis: Phase 2 Randomized Trial Results
Source: PLoS One. 2014 Sep 4;9(9):e106195. doi: 10.1371/journal.pone.0106195 (PMC4154718; doi:10.1371/journal.pone.0106195)
Supplement: File S1 — Supporting information file. (DOCX) [file pone.0106195.s001.docx]

**Supporting information**

**Supplementary Methods**

**Exclusion Criteria**

Exclusion criteria were known hypersensitivity to study drug or its components or known medication allergy; participation in another study with an investigational drug within 1 month or six half-lives (whichever was greater) preceding the screening visit; risk of not complying with or abusing the study drug administration; pregnant or lactating females or those of child-bearing potential who were not using a medically approved form of contraception; commencement of a new long-term medication for cystic fibrosis (CF) within 4 weeks of screening (cycling tobramycin inhalation solution [TIS] regimen must have completed at least two cycles of every other month of TIS administration prior to the screening visit—the last TIS cycle should have been performed 2 weeks prior to study entry); and clinically significant disease or medical condition other than CF or CF-related conditions that, in the opinion of the investigator, would have compromised the safety of the patient or the quality of the data.

**Changes in the Conduct of the Trial or Planned Analyses**

Protocol was amended to implement change in the inclusion criterion pre-bronchodilator forced expiratory volume in 1 second (FEV_1_) at start of treatment from 10% to 15% of the value at screening. A 15% change in FEV_1_ is within the normal range of variability for the CF population, giving the sites greater flexibility for including patients.

**Blinding and Randomization**

Boehringer Ingelheim generated the randomization schedule using simple blocked randomization with a block size 6, prepared and coded the medication in a blinded fashion, and provided all study supplies. The patients were allocated into the three treatment groups in a 1:1:1 ratio. An emergency code break was available to the investigator. Prior to unblinding of the trial database, the random code was forwarded to the bioanalytical laboratory to conduct the analytical determinations. The responsible person confirmed in writing that the random plan was treated confidentially and that all unblinding information was restricted to the laboratory staff.

**Sample Size**

From a previous study [1], an appropriate estimate of the expected standard deviation for FEV_1_ percent-predicted value at screening was 15.66. In this study, the ratio of adult to pediatric patients was approximately 2:1. To detect a difference of five units in the percentage change from baseline in FEV_1_ values between tiotropium and placebo with 80% power at two-sided level of significance level of 5%, 155 patients per group were required.

**Respiratory and Systemic Symptoms Questionnaire (RSSQ)**

At weeks 0, 2, 4, and 12, the RSSQ was administered to each patient aged ≥6 years in the available language version to capture the presence or absence of the following signs and symptoms: increased sputum production; change in sputum appearance; increased chest congestion; new or increased coughing up of blood; increased cough; decreased exercise tolerance; increased dyspnea with exertion; malaise, fatigue, or lethargy; fever (temperature >38°C); anorexia or weight loss; sinus pain or tenderness; change in sinus discharge; school or work absenteeism due to illness; decreased appetite.

The investigators assessed the presence or absence of the following based on physical findings and pulmonary functions for each patient: new finding on chest examination (e.g., wheezing, rattles, crackles); decline in FEV_1_ >10% since the previous visit; radiographic changes (if a chest x-ray was clinically shown) indicative of pulmonary infection. The results of the patient and investigator assessments of signs and symptoms possibly indicative of worsening CF were used to determine the presence or absence of exacerbations using pre-defined definitions [2,3]. Pulmonary exacerbations were defined requiring treatment with intravenous antibiotics for any four of the 12 signs or symptoms assessed by the RSSQ questionnaire.

**Residual Volume/Total Lung Capacity (RV/TLC)**

Measurements were performed according to ATS/ERS recommendations on standardization of lung volume measurements [4]. Static lung volumes were measured by body plethysmography at randomization and at week 12 (–15 minutes prior to study drug inhalation and 3 hours 45 minutes after inhalation of study drug). RV and TLC were calculated from body plethysmographic measurement of functional residual capacity.

**CF Questionnaire – Revised (CFQ-R)**

Health-related quality of life was assessed via the CFQ-R, administered at weeks 1 and 12. This is a disease-specific instrument that measures health-related quality of life for adolescents and adults with CF. This validation questionnaire consisted of 50 items on generic and disease-specific scales. For pediatric patients, the CFQ-R was administered to the patient and, where appropriate for patients aged 6–13 years, also to their parent/caregiver.

**Handling of Missing Data**

Every effort was made to collect FEV_1_ data at the specified time points, except if the patient used rescue medication. Randomly missing data were linearly interpolated when data were available from the visit before and after inhalation. Randomly missing data with no subsequent non-missing values for that visit were imputed using the last observation carried forward technique to calculate peak and area under the curve (AUC) and for obtaining values for the analysis of individual time points. If a patient discontinued the trial due to worsening CF, his/her subsequent missing data were imputed by his/her least favorable value observed at that time point. All other cases of missing data (e.g., due to early dropout) were considered missing at random and were not imputed. The likelihood-based repeated measures mixed-effects model described handled missing data under those situations. The above rules for imputing missing data were used in primary analyses. A sensitivity analysis without imputation was performed and yielded similar results.

**Supplementary Results**

**Pulmonary Function Tests**

For pre-bronchodilator percent-predicted forced expiratory flow from 25% to 75% of vital capacity, both doses of tiotropium had estimated adjusted mean improvements from baseline that were greater than placebo (tiotropium 2.5 μg: 4.19% difference [2.78% vs. –1.40%], p=0.0363; tiotropium 5 μg: 5.34% difference [3.94% vs. –1.40%], p=0.0073). Adjusted mean post-dose changes from baseline in RV/TLC were similar in all treatment groups at the end of week 12 (placebo: –0.01; tiotropium 2.5 μg: 0.00; tiotropium 5 μg: 0.04) and adjusted mean trough response (placebo: 0.00; tiotropium 2.5 μg: 0.01; tiotropium 5 μg: 0.04).

**Pulmonary Exacerbation as Assessed by the RSSQ**

Logistic regression analysis indicated that patients treated with placebo were slightly more likely to have a pulmonary exacerbation (16 of 167, 9.6%) than patients treated with tiotropium 2.5 μg (13 of 166, 7.8%; odds ratio [OR]: 0.79; 95% confidence interval [CI] for the OR 0.037–1.72; p=0.8515) and tiotropium 5 μg (12 of 175, 6.9%; OR: 0.72; 95% CI for the OR 0.33–1.59; p=0.5565); these differences were not statistically significant. Except for one pulmonary exacerbation in each treatment group, all events occurred in patients aged ≥12 years. Logistic regression analysis of the two tiotropium groups combined compared with placebo showed similar results overall.

**CF Health-Related Quality of Life as Assessed by CFQ-R**

There was no treatment benefit in adult patients or in patients aged between 6 and 13 years (regardless of administration of the questionnaire to the child or the parent). Though there were isolated trends, these findings are not considered clinically relevant due to the lack of consistent trends and small sample sizes.

**Adverse Events (AEs)**

The incidence of AEs was approximately 8% higher in younger patients than in older patients (≥12 years), whereas the majority of severe AEs, drug-related AEs, serious AEs, “other” significant AEs, and AEs leading to study drug discontinuation were reported in older patients. These differences were not related to treatment or tiotropium dose, and are consistent with AE frequency profiles in CF. There were no clinically relevant findings with respect to laboratory evaluations or vital signs.

**References**

1. Koker P, Bhattacharya S, Staab A (2007) A randomized, double-blind, placebo controlled study to investigate the efficacy and safety of 24 weeks of oral treatment with BIIL 284 BS in adult (75 mg, 150 mg) and pediatric (75 mg) cystic fibrosis patients. Boeringher Ingelheim Report.

2. Fuchs HJ, Borowitz DS, Christiansen DH, Morris EM, Nash ML, et al (1994) Effect of aerosolized recombinant human DNase on exacerbations of respiratory symptoms and on pulmonary function in patients with cystic fibrosis. The Pulmozyme Study Group. N Engl J Med 331: 637-642.

3. Rosenfeld M, Emerson J, Williams-Warren J, Pepe M, Smith A, et al (2001) Defining a pulmonary exacerbation in cystic fibrosis. J Pediatr 139: 359-565.

4. Miller MR, Hankinson J, Brusasco V, Burgos F, Casaburi R, et al (2005) Standardisation of spirometry. Eur Respir J 26: 319-338.
