# Supplementary material for: Testing Two Different Doses of Tiotropium Respimat® in Cystic Fibrosis: Phase 2 Randomized Trial Results
Source: PLoS One. 2014 Sep 4;9(9):e106195. doi: 10.1371/journal.pone.0106195 (PMC4154718; doi:10.1371/journal.pone.0106195)
Supplement: Protocol S1 — Trial Protocol. (PDF) [file pone.0106195.s002.pdf]

Clinical Trial Protocol Revision A (Including Amendment No. 1)

Text marked in Bold followed by ***(Rev A)***

Doc. No.: U08-3220-02

**EudraCT No.:**	2008-001156-43
**BI Trial No.:**	205.339<br>Including Amendment No. 1
**Investigational Products:**	Tiotropium bromide solution in Respimat® inhaler
**Title:**	A randomized, double-blind, placebo-controlled parallel group study to investigate the safety and efficacy of two doses of tiotropium bromide (2.5 µg and 5 µg) administered once daily via the Respimat® device for 12 weeks in patients with cystic fibrosis
**Clinical Phase:**	II
**Trial Clinical Monitor:**	Kay Tetzlaff, M.D.<br>Boehringer Ingelheim Pharmaceuticals, Inc.<br>900 Ridgebury Road<br>P.O. Box 368<br>Ridgefield, CT 06877-0368<br>Phone: +1 203 798-4034<br>Fax: +1 203 791-6158
**Co-ordinating Investigator:**	Prof P. Stuart Elborn<br>Belfast City Hospital<br>Adult CF Centre<br>Ground Floor, Lisburn Rd<br>Belfast BT9 7AB<br>Northern Ireland, UK<br>Phone: +44 28 90 263683<br>Fax: +44 28 90 263546
**Date of Protocol:**	**Original** 26 March 2008<br>**Revision A** 6 October 2008
**Planned Dates of Trial:**	May 2008 to January 2010

Proprietary confidential information.
© 2008 Boehringer Ingelheim International GmbH or one or more of its affiliated companies. All rights reserved.
This document may not - in full or in part - be passed on, reproduced, published or otherwise used without prior written permission.

REV A

CLINICAL TRIAL PROTOCOL REVISION PAGE

I herewith certify that this Clinical Trial Protocol Revision (Revision A) gives an accurate and complete revision of the protocol, including *the* Amendment*(s)* No. 1.

Trial Clinical Monitor 6 Oct 2008
Date

Dr. Kay Tetzlaff
Clinical Research- Respiratory

The official documents are the original protocol and applicable amendments. This unofficial copy of the protocol does not require signature, and therefore, the signature pages remain blank.

REV A

CLINICAL TRIAL PROTOCOL SYNOPSIS

**Name of company:**<br><br>Boehringer Ingelheim	**Tabulated Trial Protocol**	
**Name of finished product:**<br><br>Tiotropium bromide		
**Name of active ingredient:**<br><br>Tiotropium bromide		

**Protocol date**<br>26 March 2008	**Trial number**<br>205.339	**Planned Trial period**<br>May 2008 to January 2010

**Title of trial:**	A randomized, double-blind, placebo-controlled parallel group study to investigate the safety and efficacy of two doses of tiotropium bromide (2.5 µg and 5 µg) administered once daily via the Respimat® device for 12 weeks in patients with cystic fibrosis
**Co-ordinating Investigator):**	Prof P. Stuart Elborn<br>Belfast City Hospital<br>Adult CF Centre<br>Ground Floor, Lisburn Rd<br>Belfast BT9 7AB<br>Northern Ireland, UK
**Trial sites:**	60 sites worldwide
**Clinical phase:**	II
**Objectives:**	This study evaluates the effects of 12-week treatment with two doses of tiotropium bromide (2.5 µg q.d. and 5 µg q.d.) compared to placebo administered via the Respimat® device on lung function in patients with CF
**Methodology:**	Randomized, double-blind, placebo-controlled, parallel design
**No. of patients:**	
**total:**	465 randomized
**each treatment:**	155 randomized
**Diagnosis and main criteria for inclusion:**	Male or female cystic fibrosis patients; FEV_1 ≥25% of predicted
**Test products:**	Tiotropium solution for inhalation
**dose:**	2.5 µg q.d. (2 inhalations of 1.25 µg) and 5 µg q.d. (2 inhalations of 2.5 µg)
**mode of admin. :**	Oral inhalation via the Respimat® device.
**Reference therapy:**	Placebo solution for inhalation
**dose:**	Not applicable
**mode of admin. :**	Oral inhalation via the Respimat® device.
**Duration of treatment:**	12 weeks

**Name of company:**<br><br>Boehringer Ingelheim		**Tabulated**<br>**Trial Protocol**	
**Name of finished product:**<br><br>Tiotropium bromide			
**Name of active ingredient:**<br><br>Tiotropium bromide			
**Protocol date**<br>26 March 2008	**Trial number**<br>205.339	**Planned Trial period**<br>May 2008 to January 2010	
**Criteria for efficacy:**	Co-primary endpoints: FEV_1 $AUC_{0\text{-}4h}$ (first) and trough FEV_1 (second) at 12 weeks.<br>Secondary endpoints: FVC $AUC_{0\text{-}4h}$, trough FVC, $FEF_{25\text{-}75}$ % predicted, RV/TLC, exacerbations as assessed by the RSSQ		
**Criteria for pharmacokinetics:**	Plasma and urine concentrations of tiotropium bromide		
**Criteria for safety:**	Physical exams, vital signs, laboratory tests, adverse events		
**Statistical methods:**	Hierarchical testing procedure will be adapted to test for primary and secondary PFT endpoints between each of two active study medications and placebo.<br>Linear mixed effect models will be used to analyze PFT endpoints. Mantel-Haenszel test will be used to test for the rate of exacerbations between active drug and placebo		

REV A

FLOW CHART

**Trial Phase**		**Screen**	**On Treatment**					**Follow-up**
**Visit**	**0**	**1**	**2**	**3[1]**	**4**	**5**	**6**	**7**
**Day**	**--**	**-7 to -10**	**1**	**15[2]**	**29[2]**	**57[2]**	**85 ±14**	**Visit 6 + 30[2]**
Informed consent	X							
Inclusion/exclusion criteria		X	X					
Physical examination (including vital signs)		X	X		X	X	X[8]	X
History/Demographics		X						
Laboratory (chemistry, haematology)		X					X[8]	
Laboratory (pregnancy;if applicable)		X						
Respimat® Training		X	X		X	X		
Randomization			X					
Drug administration			X		X	X	X	
Dispense medication			X		X	X		
Return unused medication					X	X	X[8]	
RSSQ[3]		X	X		X		X[8]	
**CFQ** ***(Rev A)***			X				X	
Spirometry		X	X[9]		X	X	X[8,9]	X
Body plethysmography[4]			X				X	
PK – blood[5]and urine[6]							X	
Adverse events		X	X	X	X	X	X[8]	X
Concomitant medication		X	X	X	X	X	X[8]	X
Conclude patient participation								X
Life status follow-up[7]								X

1 Visit 3 may be conducted as phone visit
2 Visit may be performed within ±2 days of scheduled visit
3 RSSQ to be used only in the available languages and patients aged 6 and above
4 Body plethysmography only to be performed at sites where a body plethysmograph is available
5 **Pre-dose (-0.10 min.) and 5 min., 20 min., 1 hr and 2 hr post-dose samples will be obtained in a subpopulation of 80 patients aged ≥12 years and a single 5 min. post-dose sample will be obtained in another subpopulation of 20-40 patients aged ≤11 years only** ***(Rev A)***
6 Pre-dose and 0-2 and 2-4 hour post-dose urine collection (applies to all participating patients)
7 Must be performed once on the originally scheduled Visit 7 date in all patients who discontinue treatment early
8 Must be performed if patient discontinues the trial early
9 Spirometry to be done 30 min. prior to dosing and 30 min., 1, 2, 3 and 4 hrs post-dosing

REV A

TABLE OF CONTENTS

Page

TITLE PAGE 1
CLINICAL TRIAL PROTOCOL REVISION PAGE 2
CLINICAL TRIAL PROTOCOL SYNOPSIS 3
FLOW CHART.. 5
TABLE OF CONTENTS 6
ABBREVIATIONS.. 9
1. INTRODUCTION 12
1.1 MEDICAL BACKGROUND... 12
1.2 DRUG PROFILE.. 12
1.3 RATIONALE FOR PERFORMING THE TRIAL.. 13
1.4 BENEFIT - RISK ASSESSMENT 13
2. TRIAL OBJECTIVES 14
2.1 GENERAL AIM - OBJECTIVES.. 14
2.2 PRIMARY ENDPOINTS.. 14
2.3 SECONDARY ENDPOINTS... 14
2.4 SAFETY ENDPOINTS 14
2.5 PHARMOCOKINETICS... 15
3. DESCRIPTION OF DESIGN AND TRIAL POPULATION............ 16
3.1 OVERALL TRIAL DESIGN AND PLAN - DESCRIPTION......................... 16
3.2 DISCUSSION OF TRIAL DESIGN, INCLUDING THE CHOICE OF CONTROL GROUPS... 16
3.3 SELECTION OF TRIAL POPULATION 17
3.3.1 Inclusion criteria 17
3.3.2 Exclusion criteria... 18
4. TREATMENTS 19
4.1 TREATMENTS TO BE ADMINISTERED... 19
4.1.1 Identity of investigational products... 19
4.1.2 Method of assigning patients to treatment groups.................................... 20
4.1.3 Selection of doses in the trial.. 20
4.1.4 Selection and timing of doses for each patient... 20
4.1.5 Blinding... 20
4.1.7 Storage conditions.. 21
4.2 CONCOMITANT THERAPY... 22
4.2.1 Rescue medication and additional treatments... 22
4.2.2 Restrictions 23
4.3 TREATMENT COMPLIANCE... 23

Page
5. OBSERVATIONS 25
5.1 EFFICACY - CLINICAL PHARMACOLOGY OR PHARMACODYNAMICS 25
5.2 SAFETY 28
5.3 OTHER 30
5.4 APPROPRIATENESS OF MEASUREMENTS 30
5.5 DRUG CONCENTRATION MEASUREMENTS - PHARMACOKINETICS 30
5.5.1 Methods and timing of sample collection 31
5.5.2 Analytical determinations 32
5.6 BIOMARKER - PHARMACODYNAMIC SAMPLING 32
5.6.1 Methods and timing of sample collection 32
5.6.2 Analytical determinations 33
5.7 PHARMACOKINETIC - PHARMACODYNAMIC RELATIONSHIP 33
5.8 DATA QUALITY ASSURANCE 33
6. INVESTIGATIONAL PLAN 34
6.1 VISIT SCHEDULE 34
6.2 TRIAL PROCEDURES AT EACH VISIT 34
6.2.1 Screening 34
6.2.1.1 Visit 1 34
6.2.2 Treatment 35
6.2.2.1 Visit 2 35
6.2.2.2 Visit 3 (phone) 36
6.2.2.3 Visit 4 36
6.2.2.4 Visit 5 37
6.2.2.5 Visit 6 37
6.2.3 End of trial (EoT) and follow-up 38
6.2.3.1 Visit 7 38
6.3 REMOVAL OF PATIENTS FROM THERAPY OR ASSESSMENT 38
7. STATISTICAL METHODS AND DETERMINATION OF SAMPLE SIZE 40
7.1 STATISTICAL DESIGN - MODEL 40
7.2 NULL AND ALTERNATIVE HYPOTHESES 40
7.3 PLANNED ANALYSES 41
7.3.1 Primary analyses 41
7.3.4 Interim analyses 42
7.3.5 Pharmacokinetic methods 42
7.4 HANDLING OF MISSING DATA 42

Page

7.5 RANDOMISATION 43
7.6 DETERMINATION OF SAMPLE SIZE 43
8. ADMINISTRATIVE MATTERS 44
8.1 ETHICS 44
8.1.1 Independent Ethics Committee or Institutional Review Board 44
8.1.2 Patient Information and Informed Consent 45
8.2 RECORDS 45
8.2.1 Drug accountability 45
8.2.2 Emergency code break 46
8.2.3 Case Report Forms (CRFs) 46
8.2.4 Source documents 46
8.2.5 Direct access to source data - documents 47
8.3 QUALITY ASSURANCE AUDIT 47
8.4 PROCEDURES 47
8.4.1 Adverse events 47
8.4.2 Emergency procedures 49
8.5 RULES FOR AMENDING PROTOCOL 49
8.6 DISCONTINUATION OF THE TRIAL BY THE SPONSOR 49
8.7 STATEMENT OF CONFIDENTIALITY 50
8.8 PUBLICATION POLICY 50
8.9 COMPLETION OF TRIAL 50
9. REFERENCES 51
9.1 PUBLISHED REFERENCES 51
9.2 UNPUBLISHED REFERENCES 53
10. APPENDICES 54
APPENDIX 10.1 RESPIRATORY AND SYSTEMIC SYSTEMS QUESTIONNAIRE (RSSQ) 54
APPENDIX 10.2 PREDICTED EQUATIONS FOR PULMONARY FUNCTION 64
APPENDIX 10.3 USE OF THE RESPIMAT DEVICE 69
APPENDIX 10.4 USE OF AN MDI 74
APPENDIX 10.5 PHARMACOKINETIC METHODS 75
10.5.1 Planned analyses for pharmacokinetic evaluations 75
10.5.2 Handling of missing data 75
APPENDIX 10.6 CYSTIC FIBROSIS QUESTIONNAIRES 79

ABBREVIATIONS

Ae	Amount of drug eliminated
AE	Adverse Event
ALT	Alanine aminotransferase
AP	Alkaline phosphatase
AST	Aspartate aminotransferase
ATS	American Thoracic Society
AUC	Area under the curve
BI	Boehringer Ingelheim
BIPI	Boehringer Ingelheim Pharmaceuticals, Inc.
BLQ	Below the limit of quantification
BP	Blood Pressure
C_{max}	maximum measured concentration
CA	Competent authority
CF	Cystic fibrosis
CL_R	Renal clearance
$CL/F_{,ss}$	Apparent clearance in the plasma after extravascular administration
CML	Clinical Monitor Local
COPD	Chronic obstructive pulmonary disease
CRA	Clinical Research Assistant/Associate
CRF/eCRF	Case Report Form / electronic Case Report Form
CRO	Clinical Research Organisation
CTMF	Clinical Trial Master File
CTP	Clinical Trial Protocol
CTR	Clinical Trial Report
CV	Coefficient of variation
DCF	Data Clarification Form
DMPK	Drug Metabolism and Pharmacokinetics
DMC	Data monitoring committee

REV A

DOC	Documentation of Change
EDC	Electronic Data Capture
EDTA	Ethylenediaminetetraacetic acid
EOT	End of Trial
ERS	European Respiratory Society
EU	European Union
FAS	Full Analysis Set
FDA	Food and Drug Administration
fe	Fraction of drug eliminated
FEF_{25-75}	Forced expiratory flow (at 25-75% of vital capacity) [also known as MMEF, maximum mid-expiratory flow]
FEV_1	Forced expiratory volume in one second
FVC	Forced vital capacity
GGT	Gamma-glutamyl transferase
GCP	Good Clinical Practice
HCG	Human chorionic gonadotropin
HPLC	High performance liquid chromatography
EC/IEC	(Independent) Ethics Committee
ICH	International Conference on Harmonisation
IND	Investigational New Drug
IRB	Institutional review board
ISF	Investigator Site File
IUD	Intrauterine Device
$\lambda_{z,ss}$	terminal rate constant in plasma
LABA	Long Acting Beta Agonist
LDH	Lactic Dehydrogenase
LOCF	Last observation carried forward
MDI	Meter Dose Inhaler
MMRM	Mixed effects model with repeated measures
MRT	Mean residence time

REV A

MS/MS	Tandem mass spectrometry
N	Number
NA	Not applicable
NC	Not calculated
NOA	Not analyzed
NOP	No peak detectable
NOR	No valid result
NOS	No sample
OPU	Operative Unit (of BI)
PBO	Placebo
PFTs	Pulmonary Function Tests
PK	Pharmacokinetic(s)
PPS	Per Protocol Set
PTM	Planned Time
RDC	Remote Data Capture
REML	Restricted maximum likelihood
RSSQ	Respiratory and Systemic Symptoms Questionnaire
RV	Residual volume
SAE	Serious Adverse Event
SGOT	Serum glutamate-oxalacetate transaminase
SGPT	Serum glutamate-pyruvate transaminase
SOP	Standard Operating Procedure
$t_{1/2}$	terminal half-life
$t_{max,ss}$	Time from dosing to maximum concentration in plasma
TLC	Total lung capacity
TOBI®	Tobramycin treatment
$V/F_{,ss}$	apparent volume of distribution during the terminal phase λ_z following an extravascular dose

REV A

1. INTRODUCTION

1.1 MEDICAL BACKGROUND

Cystic fibrosis (CF) is an inherited autosomal recessive disease that disrupts ion transport in epithelial-lined organs, including pulmonary airways, sweat ducts, pancreatic ducts and intestine [R05-1407, R01-1277]. Because obstruction of pulmonary airways is the cause of death of more than 90% of the patients with CF, understanding and treatment of the pathophysiology of CF lung disease is an urgent goal. New medications to address specific elements of pulmonary obstruction may provide benefit to CF patients.

Standard therapy of CF patients includes antibiotics, airway clearance techniques and devices, pancreatic enzymes and nutritional supplements, and drugs such as dornase alfa, ibuprofen and, most often, inhaled bronchodilators [R05-1456].

Only two medications have CF as an approved indication, those being Pulmozyme® and inhaled Tobramycin®, so the remaining inhaled agents are used off-label since there is no bronchodilator with an approved indication for CF and, yet, approximately 80% of CF patients use short- and/or long-acting bronchodilators although their use as a therapy is not clearly defined [P04-10376]. The available evidence indicates that bronchodilator agents in general are able to improve pulmonary function and lessen wheezing in many patients with cystic fibrosis. There is also a suggestion that CF patients with milder lung disease and better lung function respond better to bronchodilators than do patients with more advanced disease [P04-10376]. In addition, the approximately equal effectiveness of β_2-agonists and vagal efferent blocking agents suggests that much of the airway obstruction in cystic fibrosis is parasympathetically mediated. It is, therefore reasonable to conclude that anticholinergics such as ipratropium bromide or tiotropium bromide could make an important contribution to reversing airway obstruction caused by bronchospasm and accumulated airway secretions [P96-1115].

1.2 DRUG PROFILE

Tiotropium is a non-chiral, quaternary ammonium compound developed as a long-acting anticholinergic bronchodilator for treatment of bronchospasm and dyspnea in patients with chronic obstructive pulmonary disease (COPD). It is two to four-fold more potent than ipratropium bromide and has a slower onset of action. The duration of action for tiotropium exceeds 24 hours compared to 6 hours for ipratropium bromide. As with other inhaled quaternary anticholinergic agents, tiotropium does not cross the blood-brain barrier to any relevant extent. It has a low oral bioavailability. Clinical trials have established that the adverse event profile of tiotropium follows that of ipratropium bromide. With multiple doses of tiotropium up to 144 μg, there were no clinically significant changes in vital signs or laboratory parameters.

For further information regarding tiotropium bromide inhalation spray, please refer to the tiotropium bromide Investigator's Brochure for CF [U08-3223-01].

REVA

1.3 RATIONALE FOR PERFORMING THE TRIAL

Since there is evidence of reversible airway obstruction in many patients with cystic fibrosis and of a significant role of parasympathetic mediation of bronchoconstriction, anticholinergic treatment may prove to be a valuable add-on to the standard of care of these patients. Ipratropium bromide, a short-acting anticholinergic compound, has been investigated in single dose studies and has in general demonstrated an increase in lung function [P89-1237, P92-1920, P92-6329]. However the long term effects of maintenance therapy with ipratropium bromide have not been studied in CF. Tiotropium, either as a dry powder (delivered via the HandiHaler®, 18 µg) or as an aqueous solution in the Respimat® device (at doses of both 5 and 10 µg), has consistently shown superior efficacy to ipratropium [U04-3400, U04-3343]. Therefore, it is expected that tiotropium has the potential to alleviate airway obstruction and symptoms of breathlessness in patients with cystic fibrosis. Tiotropium also has a 24-hour duration of action which may improve compliance and ease the burden on patients who already utilize a large medication regimen.

1.4 BENEFIT - RISK ASSESSMENT

Based on completed studies in animals and humans, tiotropium bromide was observed to have a favorable safety profile. Adverse events were observed that can be attributed to the anticholinergic mechanism of action. The most common adverse event that was related to tiotropium was dry mouth.

A Data Monitoring Committee (DMC) will be established to advise Boehringer Ingelheim regarding the continuing safety of study subjects, both those currently participating and those yet to be recruited. A DMC charter will be developed prior to initiation of the study to clearly describe the specific responsibilities (e.g., recommending that the trial continue or stop based on their review of data) of the DMC during the trial. Specific emphasis will be placed on any safety issues that might have been noted in the 205.338 trial. In addition, if patients discontinue early from the study, they will be followed until the time that they would have completed the study.

REV A

2. TRIAL OBJECTIVES

2.1 GENERAL AIM - OBJECTIVES

This study evaluates the effects of 12-week treatment with two doses of tiotropium bromide (2.5 µg q.d. and 5 µg q.d.) compared to placebo administered via the Respimat® device on lung function in patients with CF.

The selection of the optimal dose will be based on bronchodilator efficacy, safety evaluations and pharmacokinetic evaluations. Treatment with tiotropium or placebo will be on top of usual maintenance therapy in study patients.

2.2 PRIMARY ENDPOINTS

The co-primary endpoints for this trial are:

- Change from baseline in pct. predicted forced expiratory volume in one second (FEV_1) area under the curve $(AUC)_{0\text{-}4h}$ at the end of Week 12 (Visit 6)
- **Change from baseline in pct. predicted trough FEV_1 at the end of Week 12 (Visit 6) *(Rev A)***

2.3 SECONDARY ENDPOINTS

- Change from baseline in forced vital capacity (FVC) $AUC_{0\text{-}4h}$ at the end of Week 12 (Visit 6)
- Change from baseline in trough FVC at the end of Week 12 (Visit 6)
- Change from baseline in pre-bronchodilator mean forced expiratory flow between 25-75% of FVC ($FEF_{25\text{-}75}$) pct. predicted at the end of Week 12 (Visit 6)
- Change from baseline in static lung hyperinflation as measured by residual volume/total lung capacity (RV/TLC) at the end of Week 12 (Visit 6)
- Proportion of patients with at least 1 pulmonary exacerbation during the treatment period as assessed by the Respiratory and Systemic Symptoms Questionnaire (RSSQ); using the definition as given in Fuchs et al. [R02-1107]
- **Change from baseline in cystic Fibrosis questionnaire (CFQ) scores at the end of Week 12 (Visit 6) *(Rev A)***

2.4 SAFETY ENDPOINTS

- Changes in vital signs from treatment baseline (systolic and diastolic blood pressure and pulse)

REV A

- Adverse events (new physical examination findings at Visit 6 (end of treatment) will be recorded as adverse events)
- Laboratory evaluation (hematology, serum chemistry, urinalysis)

2.5 PHARMOCOKINETICS

- The amount of tiotropium that is eliminated in urine over a 4 hr interval post-tiotropium inhalation will be evaluated in all patients recruited in this study.
- In a subgroup of 80 patients ≥12 years old, blood samples for the purpose of pharmacokinetic characterization will be obtained until 2 hrs post-dose.
- **In a subset of 20-40 patients ≤11 years old, one blood sample will be obtained 5 min. post-dose and tiotropium plasma concentrations observed will be listed *(Rev A)*.**

Tiotropium steady state will be assumed and following parameters will be determined at Week 6, if feasible:

1.) AUC_{t1-t2} (area under the concentration time curve of tiotropium in plasma over the time interval t_1 to t_2),

2.) $C_{max,ss}$ (maximum measured concentration of tiotropium in plasma),

3.) $t_{max,ss}$ (time from dosing to the maximum concentration of tiotropium in plasma),

4.) $\lambda_{Z,SS}$ (terminal rate constant in plasma),

5.) $t_{½}$ (terminal half-life of the tiotropium in plasma),

6.) $MRT_{ih,ss}$ (mean residence time of the tiotropium in the body after inhalational administration),

7.) $CL/F_{,ss}$ (apparent clearance of the tiotropium in the plasma after extravascular administration),

8.) $V_z/F_{,ss}$ (apparent volume of distribution during the terminal phase λ_z following an extravascular dose),

9.) Ae_{t1-t2} (amount of drug that is eliminated in urine from the time point t_1 to time point t_2 (Ae_{0-2}, Ae_{2-4}, Ae_{0-4}),

10.) fe_{t1-t2} (fraction of drug eliminated in urine from time point t_1 to time point t_2 (fe_{0-2}, fe_{2-4}, fe_{0-4}),

11.) $CL_{R,t1-t2}$ (renal clearance of the drug from the time point t_1 until the time point t_2) ($CL_{R,0-2}$, $CL_{R, 2-4}$, $CL_{R,0-4}$).

Further pharmacokinetic parameters may be calculated as appropriate.

REV A

3. DESCRIPTION OF DESIGN AND TRIAL POPULATION

3.1 OVERALL TRIAL DESIGN AND PLAN - DESCRIPTION

This is a 12-week, multi-dose, multi-center, multi-national, randomized, double-blind, placebo-controlled parallel group study to determine the optimal dose of tiotropium inhalation solution delivered by the Respimat® inhaler in patients with CF.

A co-ordinating investigator has been selected for this trial. A central laboratory will be used in this trial for the standard hematology and chemistry panels. Lung function equipment for spirometry will be provided to the study sites. In addition, an external DMC will be used for this study.

3.2 DISCUSSION OF TRIAL DESIGN, INCLUDING THE CHOICE OF CONTROL GROUPS

A sufficient number of CF patients will be enrolled in the study to ensure a total of 465 (adult and pediatric) patients in the full analysis set (see Section 7.3 for definition of full analysis set); 155 patients will be assigned to placebo, 155 patients will be assigned to the 5 µg tiotropium bromide group, and 155 patients will be assigned to the 2.5 µg tiotropium bromide group. The patients will be recruited from approximately 60 Investigational sites in the United States, Canada, Europe, and Australia.

After an initial screening visit, patients will enter a 7- to 10-day screening period. Patients who meet all inclusion and exclusion criteria and have no clinically significant laboratory results will be randomized into the 12-week, double-blind treatment portion of the study in which they will receive either 2.5 µg or 5.0 µg of tiotropium bromide or placebo. Patients will be evaluated for an additional 30 days following completion of the randomized treatment period.

At Visits 2 and 6, pulmonary function testing will be done 30 min. prior to dosing and at 30 min., 1, 2, 3 and 4 hrs post-dosing. The RSSQ will be administered at Visits 1, 2, 4 and 6. Other observations include laboratory tests, adverse events, and use of concomitant therapies. **The CFQ will be administered at Visits 2 and 6 *(Rev A)*.**

A log will be kept of all patients for study enrolment and will document the reasons for exclusion for each subject not enrolled.

Every effort should be made to keep patients in the study until they complete all study procedures. Sufficient patients will be randomized to ensure that a minimum of 465 patients of either gender, with a diagnosis of CF are entered (randomized) into the study.

Recruitment is competitive. However, it is anticipated that each site will at least enrol approximately 6 patients.

REV A

3.3 SELECTION OF TRIAL POPULATION

A log of all patients screened will be maintained in the Investigator's Site File (ISF) at the investigational site.

3.3.1 Inclusion criteria

1. Male or female patients with a documented diagnosis of CF (positive sweat chloride ≥60 mEq/liter, by pilocarpine iontophoresis) and/or a genotype with two identifiable mutations consistent with CF accompanied by one or more clinical features with the CF phenotype

2. Patients must be able to perform acceptable spirometric maneuvers, according to the American Thoracic Society (ATS) standards

3. Pre-bronchodilator FEV_1 ≥25% of predicted values* (Appendix 10.2)

* pediatric/adolescent (up to 18 years of age, inclusive) predicted equations from: Wang X et al. Pulmonary function between 6 and 18 years of age. Pediatr Pulmonol 1993;15:75-88 [R02-1109]

* adult (>18 years) predicted equations from: Knudson RJ et al. Changes in normal maximal expiratory flow-volume curve with growth. Am Rev Respir Dis 1983;127:725-734 [R98-1298]

4. Patients must be able to inhale medication in a reproducible manner from the Respimat® inhaler and from a metered dose inhaler (MDI)

5. Patients must be clinically stable as defined by:

   a. no evidence of acute upper or lower respiratory tract infection within 4 weeks of screening

   b. no pulmonary exacerbation requiring use of i.v./oral/inhaled antibiotics, or oral corticosteroids within 4 weeks of screening (Visit 1)

   c. Pre-bronchodilator FEV_1 at Visit 2 must be within 10% of FEV_1 at Visit 1. If pre-bronchodilator FEV_1 at Visit 2 is not within 10% of FEV_1 at Visit 1, Visit 2 may be re-scheduled once within 7 days

6. The patient or the patient's legally acceptable representative must be able to give informed consent in accordance with International Conference on Harmonization (ICH) Good Clinical Practice (GCP) guidelines and local regulation

7. Patients taking a chronic medication must be willing to continue this therapy for the entire duration of the study.

3.3.2 Exclusion criteria

1. Patients with a known hypersensitivity to study drug or its components or known medication allergy that is deemed relevant to the trial as judged by the Investigator. “Relevance” in this context refers to any increased risk of hypersensitivity reaction to trial medication

2. Patients who have participated in another study with an Investigational drug within one month or six half-lives (whichever is greater) preceding the screening visit

3. Patients who are currently participating in another trial. Observational studies are allowed. Permission should be obtained from sponsor of other study

4. Patients with known relevant substance abuse, including alcohol or drug abuse. The intention of this criterion is to exclude patients who are considered to be at risk of not complying with or abusing the trial medication administration directives

5. Female patients who are pregnant or lactating, including females who have a positive serum pregnancy test at screening (pregnancy tests will be performed for all females of child bearing potential)

6. Female patients of child bearing potential who are not using a medically approved form of contraception. The ICH Document M3, defines highly effective forms of birth control as: implants, injectables, combined oral contraceptives, some intrauterine contraceptive devices (IUDs), sexual abstinence, or a vasectomized partner. And, as such, this definition will constitute medically approved forms of birth control for this study

7. Patients who have started a new chronic medication for CF within 4 weeks of screening. Patients who are on a cycling TOBI® regimen must have completed at least 2 cycles of every other month TOBI® administration prior to the screening visit. **The last TOBI® cycle should have been performed 2 weeks before study entry (Visit 2) *(Rev A)*.** The last TOBI® cycle during treatment with the investigational drug should have been performed two weeks before the last treatment visit (Visit 6). As there are other cycles used with TOBI®, the clinical monitor should be consulted before the patient is enrolled

8. Clinically significant disease or medical condition other than CF or CF-related conditions that, in the opinion of the Investigator, would compromise the safety of the patient or the quality of the data. This includes significant hematological, hepatic, renal, cardiovascular, and neurologic disease. Patients with diabetes may participate if their disease is under good control prior to screening. This criterion provides an opportunity for the investigator to exclude patients based on clinical judgment, even if other eligibility criteria are satisfied

REV A

4. TREATMENTS

4.1 TREATMENTS TO BE ADMINISTERED

4.1.1 Identity of investigational products

Tiotropium inhalation solution is administered via Respimat® inhaler. The solution is manufactured by Boehringer-Ingelheim in Ingelheim. The Respimat® inhaler is manufactured by microParts GmbH in Dortmund.

The investigational drug consists of a cartridge containing a 1.25 µg or 2.5 µg per actuation solution for administration using the Respimat® inhaler. Respimat® and cartridge will be provided as a kit.

1 mg tiotropium cation (Molecular Weight: 392.5 g/mol) equals 1.25 mg tiotropium bromide monohydrate (= BA 679 BR) (Molecular Weight: 490.4 g/mol). The doses given below refer to the tiotropium cation.

<u>Test</u>

Substance (INN):	Tiotropium
Trade name:	NA
Pharmaceutical form:	Inhalation solution
Unit strength:	2.5 µg (1.25 µg per actuation) tiotropium<br>5 µg (2.5 µg per actuation) tiotropium
Duration of treatment:	12 weeks
Route of administration:	Oral inhalation via Respimat® inhaler
Posology:	2.5 µg (1.25 µg per actuation) tiotropium: 2 inhalations once daily at the same time of day<br>5 µg (2.5 µg per actuation) tiotropium: 2 inhalations once daily at the same time of day

<u>Reference</u>

Substance (INN):	placebo
Trade name:	NA
Pharmaceutical form:	Inhalation solution
Unit strength:	NA
Duration of treatment:	12 weeks
Route of administration:	Oral inhalation via Respimat® inhaler
Posology:	placebo matching 2.5 µg tiotropium: 2 inhalations once daily at the same time of day<br>placebo matching 5 µg tiotropium: 2 inhalations once daily at the same time of day

REV A

4.1.2 Method of assigning patients to treatment groups

Patients are assigned to treatment at Visit 2. The treatment for each patient is determined by simple random assignment. After assessment of all inclusion and exclusion criteria, each eligible patient will be assigned the lowest available medication number at the investigational site at the time of randomization. Note that the medication number is different from the patient number (the latter is assigned at Visit 0). Site personnel will enter the medication number on the eCRF.

4.1.3 Selection of doses in the trial

Based on data obtained in a Phase I study in CF patients (PK, safety and tolerability) and patients with COPD (efficacy/safety), the proposed daily doses in this study are 2.5 µg and 5 µg.

4.1.4 Selection and timing of doses for each patient

Patients will be encouraged to maintain a standard therapeutic regimen (i.e., type and timing of procedures) during the trial, especially prior to clinic visits. Patients should be encouraged to take their study medication in the morning, at approximately the same time, between 6:00 AM and 10:00 AM. The rationale for this is that on the PK testing day (at Visit 6) the patient and site personnel will not need to stay late in the clinic to fulfil the 4 hour PK and PFT testing. However, if it is more convenient for the patient to routinely take their medication later in the day (at approximately the same time each day) and the required testing late in the day can be accommodated by site personnel, this will be acceptable.

4.1.5 Blinding

This study incorporates a double-blind study design. Boehringer Ingelheim will generate the randomisation schedule, will prepare and code the medication in a blinded fashion and will provide all study supplies.

An emergency code break will be available to the Investigator. Rules for breaking the code for an individual or for all subjects are described in Section 8.2.2.

Prior to unblinding of the trial database, the random code will be forwarded to the bioanalytical laboratory in order to conduct the analytical determinations as described in Section 5.5.2. In this case, the responsible person will confirm in writing that the random plan will be treated confidentially and that all unblinding information is restricted to the laboratory staff.

4.1.6 Packaging, labelling, and re-supply

All trial medication will be contained in a patient treatment box identified with the trial number and a specific medication number. **Each patient box will contain three visit-specific boxes and two reserve boxes *(Rev A)*.** The visit boxes at Visits 2, 4, and 5 will contain one Respimat® inhaler and one cartridge. The reserve boxes will contain one Respimat® inhaler and one cartridge each.

The visit specific boxes and reserve boxes will have a two part tear-off label. At the time of dispensing, one part of the tear-off label will remain on the container and the other part will be attached to a special drug-dispensing log which will be part of the Investigator Site File (ISF).

The visit specific boxes and reserve box will have a two part tear-off label. At the time of dispensing, one part of the tear-off label will remain on the container and the other part will be attached to a special drug-dispensing log which will be part of the Investigator Site File (ISF).

The label of the visit-specific boxes will include the following information (an example of the label text is provided in the ISF):

- Trial number
- Medication number
- Visit number, Reserve number
- Daily dose
- Directions for use
- Storage instructions
- Batch number
- Name of sponsor (and address and telephone number if agreed upon for local regulations)

A re-supply is planned for this study.

Emergency envelopes will be provided for emergency situations (see Section 8.2.2).

Boehringer Ingelheim will provide the following open-label supplies:

- Placebo Respimat® inhalers and cartridges and disposable mouthpieces for training purposes will be provided centrally by BI in Ingelheim
- Albuterol (Salbutamol) inhalation aerosol (MDI) (100µg per actuation) for use as rescue medication during the trial will be provided by Boehringer Ingelheim local OPUs

4.1.7 Storage conditions

Clinical supplies should be stored at 25° C (excursions are permitted to 15-30° C (59-86° F)) in a locked, secure cabinet. Do not refrigerate or freeze.

REV A

Drug supplies will be kept in their original packaging under the recommended storage conditions and may only be dispensed to trial subjects according to the protocol. Throughout the trial, drug receipt, usage and return must be documented and verified. Any discrepancies in drug supplies will be noted and explained.

4.2 CONCOMITANT THERAPY

The investigator must record all medication used by the patient for the two months preceding the screening visit and throughout the trial, on the eCRF. This record will include the name of the medication, the total daily dose, route of administration, dates when medication was started and stopped, and the indication for medication usage.

4.2.1 Rescue medication and additional treatments

Administration of rescue medication can occur at any point during the trial as deemed necessary by the patient or the investigator. Open label albuterol (salbutamol) MDI (100 µg per puff) will be provided as rescue medication by BI; only the albuterol (salbutamol) MDI provided by BI is allowed for rescue medication use. A re-supply will be dispensed at clinic visits as needed.

If the patient requires rescue medication during the pulmonary function test (PFT) days (Visits 2, 4, 5 and 6), the PFTs will be discontinued; however, the remainder of the PK testing at Visit 6 should be completed for the visit. The visits may be re-scheduled twice. The medication used, route and 24-hour clock time of administration will be recorded on the Rescue Medication eCRF page.

Trial medication will be administered as <u>add-on therapy to standard care</u>. Thus, patients will remain on their usual medication throughout the study.

Medications allowed if stabilized for at least 4 weeks prior to and throughout the study period:

- Theophylline
  Dose adjustments or discontinuation is <u>NOT</u> permitted during the study
  Will require 24 h washout for short-acting and 48 h washout for long-acting theophyllines prior to PFT testing days
- Pulmozyme®
- Mucolytics
- Anti-leukotrienes, leukotriene receptor antagonists
- Ibuprofen
- Hypertonic saline
- Cromolyn / Nedocromil
- Short-acting beta adrenergics
  Will require 6 h washout prior to PFT testing days
- Long-acting beta adrenergics (e.g., formoterol, salmeterol)
  Will require 12 hr washout prior to PFT testing days *(Rev A)*
- Oral corticosteroids

Allowed only if the patient is deemed stabilized on minimal doses by the investigator after consultation with the clinical monitor.

- Inhaled corticosteroids
  No washout required on PFT testing days
- **Long-acting beta adrenergic/ Inhaled corticosteroid fixed dose combinations (e.g., Advair®)*(Rev A)***
- Oral or Intravenous antibiotics
  May be used as medically necessary for exacerbations and/or other infections at any time during the trial
- Inhaled Antibiotics
  Daily inhaled antibiotic use is allowed if stabilized for at least 6 weeks prior to and throughout the study period. Cycled inhaled antibiotic use (e.g., TOBI® every other month) is also allowed. **Visit 2, however, has to be scheduled 2 weeks after the most recent TOBI® cycle *(Rev A)*.** In addition, the last TOBI® cycle during the treatment period has to be scheduled 2 weeks before the last treatment visit (Visit 6). **In order to comply with a 4-week on/off TOBI regimen Visit 6 may be scheduled within 10-14 weeks after Visit 2 *(Rev A)*.**

4.2.2 Restrictions

Medications NOT allowed for at least 30 days prior to Visit 1:

- All other investigational drugs
- Long-acting anticholinergics (e.g., Spiriva®)

Medications NOT allowed throughout the study period:

- All other investigational drugs
- Long- or short-acting anticholinergic drugs other than the study drug, including fixed-dose combinations of anticholinergics/beta adrenergics (e.g., Combivent®). Short-acting anticholinergics must be washed out at least 6 hours prior to Visit 1.

4.3 TREATMENT COMPLIANCE

Training on the use of the Respimat® inhaler will first be provided to the site staff who will train the patients subsequently. Each patient will be trained on the screening day as to the correct inhalation using a placebo Respimat®. A training video tailored to children will be available. This video will show the use of the Respimat® by children of different appropriate age groups, and the inhalation technique will be explained in terms a child and/or the parents can understand. All patients will be asked to return all dispensed Respimat® inhalers and cartridges to the clinic. Dosing compliance and proper use of the Respimat® inhaler will be reviewed with the patient at Visits 4 and 5.

Randomized patients will not be discontinued for lack of compliance without prior discussion with the local clinical monitor.

REV A

5. OBSERVATIONS

5.1 EFFICACY - CLINICAL PHARMACOLOGY OR PHARMACODYNAMICS

Spirometry

Spirometry will be performed according to the ATS/ERS recommendations on standardization of lung function testing [P05-12782]. Study sites will be supplied with spirometers that meet the standards as required by the ATS/ERS Task Force document [P05-12782]. Only these spirometers must be used for this trial. Spirometry is to be conducted by appropriately trained laboratory personnel, who should be identified on the Trial Staff List in the ISF. For each individual patient, spirometry must be conducted by the same technician whenever possible. The within- and between manoeuvre acceptability criteria as outlined in the ATS/ERS Task Force document [P05-12782] must be met.

Raw values and not predicted values for FEV_1, Forced Vital Capacity (FVC) and Forced Expiratory Flow at 25-75% of vital capacity (FEF_{25-75}) will be entered into the eCRF. Spirometry will be performed either as a single test (Visits 1, 4, 5, and 7) or 30 minutes prior to dosing as well as 30 minutes, 1, 2, 3 and 4 hours post dosing (Visits 2 and 6).

Body plethysmography

Static lung volumes will be measured by body plethysmography (Visits 2 and 6). Measurements will be performed according to the ATS/ERS recommendations on standardization of the measurement of lung volumes [R08-1121] at those study sites where a body plethysmograph is available. As for spirometry, body plethysmography is to be conducted by appropriately trained laboratory personnel, who should be identified on the Trial Staff List in the ISF. For each individual patient, body plethysmography must be conducted by the same technician whenever possible. Residual volume (RV) and total lung capacity (TLC) will be calculated from body plethysmographic measurement of functional residual capacity.

Respiratory and Systemic Symptoms Questionnaire (RSSQ)

The RSSQ will be used only in the available language versions and in children aged 6 years and older as well as adults (Appendix 10.1).

At Visits 1, 2, 4 and 6 the RSSQ will be administered to patients using a specific script to capture the presence / absence of the following signs and symptoms:

Patient History*

- **Increased sputum production**
- **Change in sputum appearance**
- **Increased chest congestion**
- **New or increased coughing up of blood**
- **Increased cough**
- **Decreased exercise tolerance**
- **Increased dyspnea with exertion**
- **Malaise, fatigue or lethargy**
- **Fever (temperature above 38°C)**
- **Anorexia or weight loss**
- **Sinus pain or tenderness**
- **Change in sinus discharge**
- **School or work absenteeism (due to illness)**
- **Decreased appetite**

The following questions on physical findings and pulmonary function are to be answered by the investigator at Visits 1, 2, 4 and 6 subsequent to administration of the RSSQ:

- **New findings on chest examination (e.g. wheezing, rales, crackles)** **Yes / No**
- **Decline in FEV_1 > 10% since previous visit**** **Yes / No**
- **Radiographic changes indicative of pulmonary infection***** **Yes / No**

* By patient report, presence of characteristic since previous clinic visit
** [(FEV_1 at Visit n) – (FEV_1 at Visit (n-1))] x 100 / (FEV_1 at Visit n)
*** Chest X-rays <u>not</u> required at study visits; this criterion only to be applied if X-ray is clinically indicated

The questionnaire will be used to determine the presence or absence of an exacerbation using the definition of Fuchs et al. [R02-1107] and Rosenfeld et al. [R02-1108]. The following interpretations of the definition by Fuchs et al. were made in developing the questionnaire:

- "Change in sputum" (Fuchs et al. definition) corresponds to **either** "increased sputum production" **or** "change in sputum appearance"
- "New or increased hemoptysis" (Fuchs et al. definition) corresponds to "new or increased coughing up of blood"
- "Increased dyspnea" (Fuchs et al. definition) corresponds to "increased dyspnea with exertion"
- "Temperature above 38°C" (Fuchs et al. definition) corresponds to "Fever (temperature above 38°C)"

- "Decrease in pulmonary function by 10 percent or more from a previously recorded value" (Fuchs et al. definition) corresponds to "decline in FEV_1 >10% since previous visit"
- **"**Change in physical examination of the chest" corresponds to "new findings on chest examination (e.g. wheezing, rales, crackles)"

<u>Cystic Fibrosis questionnaire (CFQ)</u>

The CFQ is a disease-specific instrument that measures health-related quality of life (HRQOL) for adolescents and adults with cystic fibrosis greater or equal 14 yrs., consisting of 50 items on generic and disease-specific scales (Appendix 10.6). The questionnaire has been validated for use in children ages 12 and 13 (to be completed by the child) and children ages 6-13 (to be completed by the parent or caregiver) ***(Rev A)***.

REV A

5.2 SAFETY

Physical Examinations

Physical examinations will be performed by the Investigator, or qualified designee, and will include height and weight at Visit 1. All body systems will be examined and any relevant findings will be documented in the source documents and concomitant diagnoses eCRF page.

Vital Signs

Blood pressure and pulse rate will be performed after the patients rest for 5 minutes. For each patient, all blood pressure recordings shall be made using the same type of instrument (i.e., manual BP recording vs. automatic digital vital signs monitor) on the same arm. To ensure that timing of PK samples is consistent with protocol specifications, drawing of blood will be performed prior to vital signs when both are required at the same timepoint.

Medical History

Medical history should include a history of CF, diagnosis date (including sweat test and genotype results), surgery history, other medical conditions, recent hospitalizations for exacerbations of CF lung disease (within last year) and any other information deemed relevant by the Investigator or Research Co-ordinator. Record all medical conditions related to CF; record non-CF medical conditions within the last year. Record medication allergies present during the past year.

Concomitant Therapies

All medications and treatments (including oxygen therapy, nutritional supplements, vitamin and herbal supplements and airway clearance) within the two months prior to the screening visit should be recorded on the CRF. Trade names for medications should be used. Any change in medication during the study should be recorded on the CRF including those medications used as treatment of an adverse event.

Significant Adverse Events

According to BI Corporate Standard Operating Procedures (SOP) 05-501-03 ("Adverse Event Processing and Reporting"), "a "significant" adverse event is an event that is defined to not meet the serious criteria, but is outlined as "significant" in the protocol of a specific trial because of its type".

No events have been classified as "significant" for this trial.

Laboratory Parameters

A central laboratory will perform the determination of laboratory parameters – information regarding shipment of blood and urine samples to the site of analysis is provided by the central laboratory and stored in the Investigator Site File.

REV A

For all patients, 3.0 mL of blood is required for each hematology sample and 4.0 mL of blood is required for each serum chemistry sample. Therefore, the total amount of blood needed for all patients in this trial is 14 mL. In addition, in a subset of patients ≥12 years of age another total of 37.5 mL of blood is required for PK analysis (see Section 5.5.1). Thus, the total amount of blood needed for these patients amounts to 51.5 mL. **Also, in a range of 20-40 patients ≤11 years old another total of 7.5 mL of blood is required for PK analysis. Thus, the total amount of blood needed for these patients amounts to 21.5 mL *(Rev A)*.**

The following parameters will be determined:

Hematology

Hematocrit

Hemoglobin

Erythrocyte count

Total and differential leukocyte count

Platelet count

Serum Chemistry

Albumin

Alanin aminotransferase (ALT) or SGOT

Aspartate aminotransferase (AST) or SGPT

Gamma-glutamyltranspeptidase (GGT)

Alkaline phosphatase (AP)

Lactic dehydrogenase (LDH)

Total bilirubin

Direct bilirubin

Indirect bilirubin

Protein (total)

Potassium

Sodium

Chloride

REV A

Inorganic phosphorus

Calcium

Creatinine

Urea

Uric acid

Glucose

Serum HCG (females of child bearing potential only; performed only at Visit 1)

5.3 OTHER

Not applicable

5.4 APPROPRIATENESS OF MEASUREMENTS

Pulmonary function tests will be performed approximately 30 minutes prior to dosing as well as 30 minutes, 1, 2, 3 and 4 hours post-dosing. Pulmonary function tests are a validated and well-established measurement tool for lung function testing

5.5 DRUG CONCENTRATION MEASUREMENTS - PHARMACOKINETICS

Plasma and urine concentration monitoring of tiotropium will be performed in order to assess drug exposure in CF patients and to characterize the pharmacokinetics of tiotropium bromide in this patient population. **Whereas urine concentration monitoring of tiotropium will be performed in all patients, plasma concentration monitoring will be done in a subset of 80 patients ≥12 years of age and another subset of 20-40 patients ≤11 years old *(Rev A)*.**

Date and exact clock time of administration of tiotropium as well as of pharmacokinetic sampling have to be recorded on Day 85 (Visit 6). In the following, the term “before” refers to a time window from -1:00 h to -5 minutes before drug administration. i.e., study measurements and assessments scheduled to occur "before" have to be performed and completed within 1 hour prior to drug administration. The time point zero for pharmacokinetic sampling is defined as end of last inhalation. Exact time points of blood sampling will be documented in the eCRFs by the medical personnel. These actual sampling times will be used for the determination of pharmacokinetic parameters. Both start and end of inhalation will be recorded on the eCRF.

For more information also see Appendix 10.5.

5.5.1 Methods and timing of sample collection

Blood sampling for pharmacokinetic analysis (subpopulation only)

In those patients ≥12 years old who volunteer to participate in the PK substudy, a total maximum amount of 51.5 mL blood will be taken per patient during the whole course (i.e., approximately 12 weeks) of the study, including blood samples for PK and laboratory evaluation (see Section 5.2). also, in another subset of 20-40 patients ≤11 years old another total of 7.5 mL of blood is required for PK analysis during the same duration.

In the subset of patients ≥12 years old pharmacokinetic profiles (i.e., from before until 2 hrs after drug inhalation) will be taken at Visit 6. Blood sampling for PK will be done at the following time points:

Visits 6: before (pre-dose = -10 min.) and 5 min., 20 min.,1 hr and 2 hrs following drug administration. For pre-dose samples a planned time of -0:10 will be used for database setup.

In the morning of the clinic visit, an indwelling catheter may be inserted in a forearm vein.

In the subset of patients ≤11 years, at Visit 6 one blood sample for PK analysis will be drawn 5 min. following drug administration *(Rev A)*.

For quantification of drug plasma concentrations, 6-7.5 mL of blood will be taken from a forearm vein using a Monovette or Vacutainer collection tube containing (K2) EDTA (ethylenediaminetetraacetic acid) as anticoagulant at the time points listed above.

Blood samples should be centrifuged; plasma will be divided into two plasma aliquots and will be frozen at -20 °C until frozen shipment to the central lab.

Detailed instructions for handling and shipment of plasma samples are provided in the ISF.

In order to avoid contamination of plasma samples, study personnel are required to wash their hands with soap prior to collecting the plasma or handling of plasma samples. The Respimat® Inhalers should be handled with gloves on and these gloves should be changed and discarded as soon as any container for PK samples is touched. As soon as the plasma is obtained from the blood, gloves should again be changed. Plasma vials should only be stored closed and only opened if necessary for the procedure.

The PK procedures should NOT take place in the same room where priming of the Respimat® Inhaler or drug inhalation takes place.

Urine sampling (all patients)

Urine collection for PK sampling will be done in all patients at Visit 6. Urine samples will be obtained at the following time points or sampling periods:

Visits 6: a pre-dose urine sample will be collected in the 1 hr prior to drug administration in the morning (in the interval of -1:00 hr to -0:05 min. prior to study drug inhalation). For pre-

dose samples a protocol start time of -1:00 and a stop time of -1:00 will be used for database setup. All urine voided during the sampling interval of 0-2 and 2-4 hrs after drug administration will be collected in containers. **Patients will be instructed to empty their bladder before the start and before the end of these two urine collection intervals *(Rev A)*.**

In order to enable a sufficient urine flow patients might be asked to drink at least 150-200 mL of a non-caffeinated beverage 15 min. prior to the end of each urine fraction in order to support a miction in time.

The urine will be collected in a container with known empty weight (container lid included in the weighing). In order to acidify the urine (tiotropium is not entirely stable at neutral pH) that will be collected, citric acid will be added to the container (see ISF). At the end of the collection interval, the weight of the filled urine container (container lid included in the weighing) will be determined and the weight of the empty container subtracted. Urine weights and times of collection will be documented in the eCRFs *(Rev A)*.

Detailed instructions for handling and shipment of urine samples are provided in the ISF.

In order to avoid contamination of urine samples, study personnel are required to wash their hands with soap prior to collecting the urine or handling of urine samples. The Respimat® Inhalers should be handled with gloves on and these gloves should be changed and discarded as soon as any container for urine samples is touched. Urine containers should only be stored closed and only opened if necessary for collection.

The urine collection procedures should NOT take place in the same room where priming of the Respimat® inhaler or drug inhalation takes place.

5.5.2 Analytical determinations

Tiotropium plasma and urine concentrations will be determined by validated HPLC-MS/MS assays at AAI Germany and will be described in an appendix to the Clinical Trial Report. Plasma concentration measurement of samples from the placebo treatment will be restricted to the blood samples taken before treatment and taken at C_{max} (i.e., plasma sample taken at 5 minutes after inhalation). Only if one of these samples reveal Tiotropium, the remaining samples of that particular patient will be analysed as well. In order to identify samples from placebo treatments, the bioanalyst will be unblinded.

5.6 BIOMARKER - PHARMACODYNAMIC SAMPLING

There will be no biomarker-pharmacodynamic sampling performed in this study.

5.6.1 Methods and timing of sample collection

Not applicable.

5.6.2 Analytical determinations

Not applicable.

5.7 PHARMACOKINETIC - PHARMACODYNAMIC RELATIONSHIP

There will be no pharmacokinetic-pharmacodynamic analysis performed in this study.

5.8 DATA QUALITY ASSURANCE

This trial will be conducted in accordance with the International Conference on Harmonization (ICH) Good Clinical Practice guidelines and local regulations.

An Investigator's meeting will take place prior to the initiation of the trial to ensure standardization of procedures and techniques across multiple sites. A central laboratory will be used to collect, analyze and report the results of all non-PK blood samples and cultures.

Data will be collected using a Remote Data Capture (RDC) system. Training will be provided to all investigators, coordinators and field monitors to ensure consistency and accuracy of the data. The data will be source verified by the field monitors before it is considered final.

REV A

6. INVESTIGATIONAL PLAN

6.1 VISIT SCHEDULE

Following the initial (screening) visit (Visit 1), patients who qualify will be randomized into the double-blind portion of the study at Visit 2. Additional visits will be scheduled after 2, 4, 8 and 12 weeks of therapy (Visits 3, 4, 5 and 6) and four weeks post-treatment (Visit 7). **Visit 6, however, may be scheduled within a time window of +/-14 days, i.e., the visit may be conducted within 10-14 weeks of therapy in order to allow flexible scheduling for those patients who undergo periodic TOBI cycling (see also 6.2.2.4) *(Rev A)*.** Visit 3 may be conducted as a phone visit only.

Patients should make every attempt to complete the study procedures as described in the study protocol. Investigators should encourage patient treatment compliance and adherence to all protocol requirements.

In the case of discontinuation of a clinic visit the local clinical monitor should be informed. All deviations from the planned visit schedule will be documented in the patient's source documents.

6.2 TRIAL PROCEDURES AT EACH VISIT

6.2.1 Screening

Informed consent will be obtained prior to patient participation in the trial, which includes any medication washout procedures or restrictions. Upon obtaining consent, the patient will be instructed on any medication washout requirements for the screening pulmonary function test at Visit 1 (medications that are not allowed during a defined period of time prior to Visit 1 are listed in Section 4.2.2).

6.2.1.1 Visit 1

The screening visit is to be scheduled seven to ten days prior to the randomization visit (Visit 2).

- Inclusion /exclusion criteria are reviewed
- Medication washout compliance will be verified
- Demographic data (gender, race, date of birth, height, weight, duration of CF) will be recorded. Height and weight should be measured using appropriate scales and entered into the eCRF in metric units (i.e., cm, kg)
- A relevant medical history and physical examination including vital signs (seated) should be conducted following five minutes at rest. Any findings from the physical examination should be recorded in the concomitant condition section in the eCRF.

- All adverse events experienced since signing informed consent will be reviewed and recorded
- Concomitant therapy for the previous two months will be recorded in the eCRF as described in Section 4.2
- Blood samples will be collected and submitted for haematology, serum chemistry and pregnancy (the latter only being done in females of child bearing potential)
- The RSSQ will be administered (if applicable; see Section 5.1)
- Each patient will be trained on the use of the Respimat® inhaler (Appendix 10.3) and MDI (Appendix 10.4).
- Spirometry will be performed. Patients must demonstrate a pre-bronchodilator FEV_1 ≥25% of predicted using the following formulae:
    - pediatric/adolescent (up to 18 years of age, inclusive) predicted equations from: Wang X, Dockery DW, et al. Pulmonary function between 6 and 18 years of age. Pediatr Pulmonol 1993;15:75-88 [R02-1109]
    - adult (>18 years) predicted equations from: Knudson RJ, Lebowitz MD, et al. Changes in normal maximal expiratory flow-volume curve with growth. Am Rev Respir Dis 1983;127:725-734 [R98-1298]
- Visit 2 will be scheduled and the patient will be reminded to follow medication washout procedures required prior to Visit 2

6.2.2 Treatment

Visits will be scheduled at the end of the screening period (Visit 2) and 2, 4, 8 and 12 weeks post-randomization (Visits 3, 4, 5, and 6).

6.2.2.1 Visit 2

- Inclusion/exclusion criteria are reviewed
- Medication washout compliance will be verified
- **The CFQ will be administered *(Rev A)***
- A physical examination including vital signs (seated) should be conducted following five minutes at rest. Any new findings in the physical examination will be recorded as AE's if they indicate a new or worsening condition
- Concomitant therapy since Visit 1 will be recorded in the eCRF as described in Section 4.2
- All adverse events experienced since Visit 1 will be reviewed and recorded
- The RSSQ will be administered (if applicable; see Section 5.1)

- The patient will be randomized to treatment
- Each patient will be trained on the use of the Respimat® inhaler
- The study drug will be administered (as part of spirometry)
- Spirometry will be performed 30 min. prior to the administration of the study drug and at 30 min., and 1, 2, 3 and 4 hrs post-dosing

  Note: Pre-bronchodilator FEV_1 at Visit 2 must be within 10% of FEV_1 at Visit 1 (see Section 3.3.1)

- Body plethysmography will be performed at 15 min. before and 3 hr 45 min. post-dosing (if applicable; see Section 5.1)
- The study drug supplies will be dispensed
- Visit 3 will be scheduled two weeks later

6.2.2.2 Visit 3 (phone)

- Concomitant therapy for the time since Visit 2 will be recorded in the eCRF as described in Section 4.2
- All adverse events experienced since Visit 2 will be reviewed and recorded
- Visit 4 will be scheduled two weeks later and the patient will be reminded to follow medication washout procedures required prior to Visit 4

6.2.2.3 Visit 4

- Medication washout compliance will be verified
- A physical examination including vital signs (seated) should be conducted following five minutes at rest. Any new findings in the physical examination will be recorded as AE's if they indicate a new or worsening condition
- The unused study medication will be returned
- New study drug supplies will be dispensed
- Concomitant therapy for the time since Visit 3 will be recorded in the eCRF as described in Section 4.2
- All adverse events experienced since Visit 3 will be reviewed and recorded
- The RSSQ will be administered (if applicable; see Section 5.1)
- Spirometry will be performed 30 minutes prior to the administration of the study drug
- Each patient will be trained on the use of the Respimat® inhaler
- New study drug will be administered
- The study drug supplies will be dispensed
- Visit 5 will be scheduled four weeks later and the patient will be reminded to follow medication washout procedures required prior to Visit 5

REV A

6.2.2.4 Visit 5

- Medication washout compliance will be verified
- A physical examination including vital signs (seated) should be conducted following five minutes at rest. Any new findings in the physical examination will be recorded as AE's if they indicate a new or worsening condition
- The unused study medication will be returned
- Concomitant therapy since Visit 4 will be recorded in the eCRF as described in Section 4.2
- All adverse events experienced since Visit 4 will be reviewed and recorded
- Spirometry will be performed 30 minutes prior to the administration of the study drug
- Each patient will be trained on the use of the Respimat® inhaler
- The study drug will be administered
- New study drug supplies will be dispensed
- Visit 6 will be scheduled. It must be considered that the last TOBI® treatment should be scheduled at least 14 days before Visit 6. **In order to allow this 2-week TOBI washout also for patients who are on a 4-week on/off TOBI regimen the visit should be scheduled between 10 and 14 weeks after Visit 2 *(Rev A)*.**

6.2.2.5 Visit 6

- Medication washout compliance will be verified
- **The CFQ will be administered *(Rev A)***
- A physical examination including vital signs (seated) should be conducted following five minutes at rest. Any new findings in the physical examination will be recorded as AE's if they indicate a new or worsening condition
- The unused study medication will be returned
- Concomitant therapy for the time since Visit 5 will be recorded in the eCRF as described in Section 4.2
- All adverse events experienced since Visit 5 will be reviewed and recorded
- Blood samples will be collected and submitted for haematology, serum chemistry
- The RSSQ will be administered (if applicable; see Section 5.1)
- The last study drug will be administered (as part of spirometry)
- Spirometry will be performed 30 min. prior to the administration of the study drug and at 30 min., and at 1, 2, 3 and 4 hrs post-dosing
- Body plethysmography will be performed at 15 min. before and 3 hr 45 min. post-dosing (if applicable; see Section 5.1)

REV A

- **In a subset of 80 patients ≥12 years old, PK blood samples will be collected at 10 min. pre-dose and at 5 min., 20 min., 1 hr and 2 hr post-dose as described in Section 5.5.1. Also, in another subset of 20-40 patients ≤11 years old, a PK blood sample will be collected at 5 min. post-dose. PK sampling is described in Section 5.5.1. *(Rev A)***
- PK urine collection will be done at pre-dose (sample taken 1 hr before dosing), 0-2 and 2-4 hrs after drug administration as described in Section 5.5.1

6.2.3 End of trial (EoT) and follow-up

End of trial procedures will be completed at Visit 7. If a patient drops early from the study the EoT procedures that need to be completed are final physical examination, laboratory tests and pulmonary function tests.

All patients, who agree in the informed consent, will be followed until the date on which they would have completed the post-treatment visit (Visit 7). Any patient who discontinues treatment early will be contacted once on the originally scheduled Visit 7 date to be questioned on vital status. This visit is listed separately in the eCRF.

6.2.3.1 Visit 7

- A physical examination including vital signs (seated) should be conducted following five minutes at rest. Any new findings in the physical examination will be recorded as AE's if they indicate a new or worsening condition
- Concomitant therapy for the time of the post-treatment period since Visit 6 will be recorded in the eCRF as described in Section 4.2
- All adverse events experienced in the post-treatment period since Visit 6 will be reviewed and recorded
- Spirometry will be performed

6.3 REMOVAL OF PATIENTS FROM THERAPY OR ASSESSMENT

Patients may be withdrawn from the trial prior to completion if any of the following criteria are observed:

- Intercurrent illness or an adverse event, which requires discontinuation of treatment
- Request by the patient to withdraw from the study
- Administrative reason (protocol violations, persistent non-compliance)
- Decision by Boehringer to discontinue one or all patients

No patient should be discontinued from the trial for a protocol violation before discussion with the clinical monitor.

REV A

Patients who fail to complete all study visits and all of the testing as specified in the protocol will **not** be considered complete and may **not** be re-enrolled at a later date. A record will be kept of all patients who fail to complete all study visits and their reasons for discontinuation.

All patients who discontinue during the randomized treatment period are to return for a final physical examination, pulmonary function tests and laboratory evaluation and return any unused study and rescue medication. In addition patients should fill in the RSSQ.

Once a patient is randomized and treated, all observations outlined in the protocol should be performed, unless the patient withdraws consent at any time (without having to justify the decision). All available data from patients who discontinued during the trial, for whatever reason, will be included in the safety analysis. As noted in Section 7.3, some data on incomplete patients will also be included in the efficacy analyses. Premature terminations will be reported to the sponsor.

REV A

7. STATISTICAL METHODS AND DETERMINATION OF SAMPLE SIZE

7.1 STATISTICAL DESIGN - MODEL

This is a 12-week, multi-center, randomized, double-blind, double dummy, placebo-controlled parallel design trial with two active treatments, tiotropium bromide (2.5 µg q.d. and 5 µg q.d.). Likelihood based mixed effects model with repeated measures (MMRM) will be used for the primary analysis. Details will be provided in Section 7.3.

7.2 NULL AND ALTERNATIVE HYPOTHESES

The main objective of the trial is to show superiority of tiotropium over placebo in change from baseline in % predicted FEV_1 AUC_{0-4H} and % predicted FEV_1 trough at the end of 12 weeks (Visit 6).

The following null and alternative hypotheses will be tested in hierarchical order, each at 2.5% level of significance (one-sided) to protect the overall probability of type I error at 0.025 (one-sided)

H_{10}: FEV_1 AUC_{0-4H} (tiotropium 5 µg) $\leq$ FEV_1 AUC_{0-4H} (placebo)
versus
H_{11}: FEV_1 AUC_{0-4H} (tiotropium 5 µg) $>$ FEV_1 AUC_{0-4H} (placebo),

H_{20}: FEV_1 trough (tiotropium 5 µg) $\leq$ FEV_1 trough (placebo)
versus
H_{21}: FEV_1 trough (tiotropium 5 µg) $>$ FEV_1 trough (placebo),

H_{30}: FEV_1 AUC_{0-4H} (tiotropium 2.5 µg) $\leq$ FEV_1 AUC_{0-4H} (placebo)
versus
H_{31}: FEV_1 AUC_{0-4H} (tiotropium 2.5 µg) $>$ FEV_1 AUC_{0-4H} (placebo),

H_{40}: FEV_1 trough (tiotropium 2.5 µg) $\leq$ FEV_1 trough (placebo)
versus
H_{41}: FEV_1 trough (tiotropium 2.5 µg) $>$ FEV_1 trough (placebo)

To address the multiple treatment comparisons a hierarchical testing procedure will be applied to maintain the overall α-level. First the tiotropium 5 µg will be compared to placebo. If and only if statistical superiority of the tiotropium 5 µg compared to placebo is demonstrated at the one sided α-level of 0.025 for both co-primary endpoints, will testing to compare the tiotropium 2.5 µg to placebo, at the same α-level of 0.025, be performed.

7.3 PLANNED ANALYSES

7.3.1 Primary analyses

The primary efficacy analysis will first compare the treatment groups in FEV_1 $AUC_{0\text{-}4H}$ calculated as percent predicted.

The statistical model for the primary analyses will be a linear mixed-effect model. Mean changes from baseline at Visits 4, 5 and 6 will be analyzed using a restricted maximum likelihood (REML)-based repeated measures approach. Visit 2 will be viewed as baseline in this study. Analyses will include the fixed effects of treatment, visit, and treatment-by-visit interaction, as well as age group, the continuous covariate of baseline measure and baseline-by-visit interaction. Centre will be modelled as random effect. An unstructured (co)variance structure will be used to model the within-patient errors. If this analysis fails to converge, the following structures will be tested: Compound Symmetry, Autoregressive Model, AR (1) or Spatial Covariance. The (co)variance structure converging to the best fit, as determined by Akaike's information criterion, will be used as the primary analysis. The Kenward-Roger approximation will be used to estimate the denominator degrees of freedom for testing the fixed effects. Analyses will be implemented using SAS. The primary treatment comparisons will be the contrast between treatments at the end of 12 weeks (Visit 6). Same analysis will also be used to compare the treatment groups in trough FEV_1 also calculated as percent predicted.

The primary analysis will performed in all randomized patients who have at least one post treatment measurement. This set will be called the Full Analysis Set (FAS).

After the blinded review of the magnitude and potential impact of any serious protocol violations, a subset of the data corresponding to those patients without serious deviations from the protocol will be created. This will be called the Per-Protocol set (PPS). If the number of patients in the PPS is less than 90% of the number of patients in the FAS, the primary analysis will also be performed on the PPS. This will be a supportive analysis.

Since the age-group is a crucial factor in this trial, sensitivity analysis will be done by including the age-group and treatment-by-age group interaction as factors in the model given the sample sizes are sensible for making inferences.

7.3.2 Secondary analyses

All secondary analyses will also be conducted in FAS. All the secondary endpoints on additional PFT parameters listed in Section 2.3 will be analyzed in the same way as in the primary analysis.

Proportions of patient with at least 1 pulmonary exacerbation during the 12-week treatment period will be evaluated using Mantel-Haenszel test adjusted for age group. Logistic regressions will be used to handle adjustment for additional covariates. The number of exacerbations and average duration of first exacerbation will be summarized for each treatment group. **Exploratory analyses, such as summary statistics will be presented for the changes of CFQ scores at the end of Week 12 (Visit 6) *(Rev A)*.**

Safety analyses

All randomized patients who receive at least one dose of study medication will be included in the safety analysis. This set will be called the Treated Set.

- Changes in vital signs (systolic and diastolic blood pressure and pulse) from baseline at the end of Week 12.
- Adverse events will be analyzed following the BI standard guidelines. Adverse events will be coded using the MedDRA dictionary. All adverse events will be classified according to the following trial periods: screening, on-treatment, post treatment. All adverse events with an onset date/time after the 1st dose of trial medication up to 30 days after the last intake of study medication will be assigned to the treatment period for evaluation. In addition, adverse events with onset date before start of the trial treatment but with worsening in intensity during the treatment will also be assigned to the on-treatment period. Other adverse events will be assigned to the screening or post treatment period, respectively. New physical examination findings at Visit 6 (end of treatment) will be recorded as adverse event.
- Laboratory evaluation (haematology, serum chemistry, urinalysis).

Safety endpoints will be summarized descriptively.

7.3.4 Interim analyses

No formal interim analysis is planned for efficacy and therefore no rule for stopping the trial early for greater efficacy is planned. An interim safety analysis will be conducted to monitor the safety of patients in the trial. An expert panel/DMC will compare the tiotropium treatment groups with placebo in the number of deaths, early discontinuations due to adverse events and serious adverse events and may choose to stop the trial early if clinically meaningful and significant dose dependent effects are noticed. The complete details will be provided in a charter.

7.3.5 Pharmacokinetic methods

Refer to Appendix 10.5

7.4 HANDLING OF MISSING DATA

<u>Missing time point data</u>

Every effort will be made to collect FEV_1 data at the specified time points, except if the patient has used rescue medication. Data missing due to worsening of symptom or need of rescue medication will be replaced with the least favourable data for that visit (including pre-dose values).

Randomly missing data after inhalation for which there are data from that visit both before and after inhalation will be linearly interpolated. Randomly missing data with no subsequent

non-missing values for that visit will be imputed using the last observation carried forward (LOCF) technique.

Missing visit

If a patient discontinues the trial due to worsening, his/her missing data will be imputed by his/her least favourable value observed to that time point. All other data will not be imputed. Likelihood based repeated measures mixed effects model described in Section 7.3.1 will handle missing data due to early drop outs appropriately; i.e., if the data are missing at random.

7.5 RANDOMISATION

The assignment of patients into one of the three treatment groups within each stratum will be randomized in a 1:1:1 ratio. Boehringer Ingelheim (BI) will determine the randomization list and will prepare and code the medication. Patients, investigators, study personnel and employee from BI will be blinded to the treatment codes until data base lock. Unbinding is only allowed in the case of emergency in order for the investigator to be fully informed to treat patients.

7.6 DETERMINATION OF SAMPLE SIZE

Results from previous CF-project BIIL284 [U07-3355] showed that the standard deviation of the patient's FEV1 percent predicted values at screening was 15.66. Moreover, adults and paediatric patients were observed to have participated in that study roughly in 2:1 ratio.

Calculation of Sample Size

Δ (%)	SD (%)	α (2-sided): Significance level (%)	1-β: Power (%)	N (per treatment group)
5	15.66	5	80	155

In summary, if 155 patients are randomized per treatment group, SD is assumed to be 15.66 and the expected difference in the percent change from baseline in FEV_1 values between the two treatment groups is 5 units, then at 5% (2-sided) level of significance the power of t-test will be 80%. The overall power will be less than 80% due to multiple comparisons.

8. ADMINISTRATIVE MATTERS

The trial will be carried out in compliance with the protocol, the principles laid down in the Declaration of Helsinki, version as of October 1996 (as long as local laws do not require to follow other versions), in accordance with the ICH Harmonised Tripartite Guideline for Good Clinical Practice (GCP) and relevant BI SOPs. Standard medical care (prophylactic, diagnostic and therapeutic procedures) remains in the responsibility of the treating physician of the patient.

The trial will be carried out in accordance with the European Medical Directive Device Directive (93/42/EEC) and the harmonized standards for Medical Devices (ISO 14155-01 and ISO 14155-02) and all other applicable regulatory requirements.

Insurance Cover: The sponsor will take out no-fault insurance cover for all patients included in the trial. The terms and conditions of the insurance cover are made available to the investigator and the patients via documentation in the ISF (Investigator Site File).

8.1 ETHICS

8.1.1 Independent Ethics Committee or Institutional Review Board

The trial will not be initiated before the protocol and informed consent and patient information form have been reviewed and received approval / favourable opinion from the local Institutional Review Board (IRB) / Independent Ethics Committee (IEC), the Competent Authority (CA) in each participating EU member state and other regulatory authorities, as required by local laws and regulations. Should a CTP amendment be made that needs *IRB / IEC* approval and authority notification/approval, the changes in the CTP will not be instituted until the amendment and revised informed consent (if appropriate) have been reviewed and received approval / favourable opinion from the local *IRB / IEC,* the CA in each participating EU member state and other regulatory authorities, as required by local laws and regulations. A CTP amendment intended to eliminate an apparent immediate hazard to patients may be implemented immediately providing that the regulatory authority and IRB / IEC are notified as soon as possible and an approval is requested. CTP amendments exclusively for logistical or administrative changes may be implemented with notification only of the *IRB / IEC, CA* in each participating EU member state and other regulatory authorities, as required by local laws and regulations.

The constitution of the IRB / IEC must meet the requirements of ICH GCP and of the participating countries. A list of the IRB / IEC members who attended the meeting when the CTP / CTP amendment was discussed, including names and qualifications, needs to be provided by the IRB / IEC to the sponsor. The sponsor must provide to the regulatory authorities the name and address of the IRB / IEC along with a statement from the IRB / IEC that it is organised according to GCP and the applicable laws and regulations. The IRB / IEC must perform all duties outlined by the requirements of ICH GCP and of the participating country / countries. The US IND requirements outlined in the US Code of Federal Regulations must be met.

8.1.2 Patient Information and Informed Consent

Prior to patient participation in the trial, written informed consent must be obtained from each patient (or the patient's legally accepted representative) according to ICH GCP and to the regulatory and legal requirements of the participating country. Each signature must be personally dated by each signatory and the informed consent and any additional patient-information form retained by the investigator as part of the trial records. A signed copy of the informed consent and any additional patient information must be given to each patient or the patient's legally accepted representative.

The patient must be informed that his/her personal trial-related data will be used by Boehringer Ingelheim in accordance with the local data protection law. The level of disclosure must also be explained to the patient.

The patient must be informed that his / her medical records may be examined by authorised monitors (CML/CRA) or Clinical Quality Assurance auditors appointed by Boehringer Ingelheim, by appropriate IRB / IEC members, and by inspectors from regulatory authorities.

Should a CTP amendment become necessary, the patient consent form and patient information form may need to be revised to reflect the changes to the CTP. It is the responsibility of the investigator and the sponsor to ensure that an amended consent form is reviewed and has received approval / favourable opinion from the *IRB / IEC* , the CA in each participating EU member state and other regulatory authorities as required by ICH GCP and by local laws and regulations, and that it is signed by all patients subsequently entered in the trial and those currently in the trial, if affected by the amendment.

8.2 RECORDS

8.2.1 Drug accountability

Drug supplies, which will be provided by the sponsor, must be kept in a secure, limited access storage area under the storage conditions defined by the sponsor. Where necessary, a temperature log must be maintained to make certain that the drug supplies are stored at the correct temperature.

The investigator / pharmacist (or investigational drug storage manager) must maintain records of the product's delivery to the trial site, the inventory at the site, the use by each patient, and the return to the sponsor or alternative disposition of unused product(s). These records will include dates, quantities, batch/serial numbers, expiry ('use by') dates, and the unique code numbers assigned to the investigational product(s) and trial patients. The investigator / pharmacist (or investigational drug storage manager) will maintain records that document adequately that the patients were provided the doses specified by the CTP and reconcile all investigational product(s) received from the sponsor. At the time of return to the sponsor, the investigator / pharmacist (or investigational drug storage manager) must verify that all unused or partially used drug supplies have been returned by the clinical trial patient and that no remaining supplies are in the investigator's possession.

8.2.2 Emergency code break

For blinded trials an emergency code break will be available to the investigator / pharmacist. This code break may only be opened in emergency situations when the identity of the trial drug must be known to the investigator in order to provide appropriate medical treatment or if required to assure safety of trial participants. If the code break for a patient is opened, the sponsor must be informed immediately. The reason for opening the code break must be documented on the envelope or appropriate CRF page along with the date and the initials of the person who broke the code.

8.2.3 Case Report Forms (CRFs)

All of the clinical data will be captured via electronic data capture (EDC) using the Oracle Clinical Remote Data Capture system, a web-based tool. The investigator site staff will enter and edit the data via a secure network, with secure access features (username, password and secure identification or username and password – an electronic password system). A complete electronic audit trail will be maintained. The investigator will approve the data using an electronic signature (Ref: 21 CFR Part 11), and this approval is used to confirm the accuracy of the data recorded.

Electronic CRFs (eCRFs) will be used for all patients. The investigator's data will be accessible from the investigator's site throughout the trial. Relevant medical history prior to enrolment will be documented at the baseline visit. Thereafter during the trial, narrative statements relative to the patient's progress during the trial will be maintained. The electronic CRFs must be kept current to reflect patient status at each phase during the course of the trial. The patients must not be identified on the electronic CRF by name. Appropriate coded identification (i.e. Patient Number) must be used. The investigator must make a separate confidential record of these details (patient identification code list) to permit identification of all patients enrolled in a clinical trial in case follow-up is required. While a trial is ongoing and until the access to the database has been terminated, there will be no Documentation of Changes (DOCs). All changes will be requested from the investigator through the EDC system. If a change is necessary once the investigator has no further access to the database, a DOC will be sent to the investigator for confirmation of the change. The investigator's signature <for Japan> or seal is requested to show he/she agrees with the change that was made. The original DOC is kept by the investigator.

Copies of the electronic CRF together with all data changes made will be supplied to the investigator at the end of the trial. The investigator will be responsible for retaining all records pertaining to the trial as specified in the appropriate contract.

8.2.4 Source documents

Source documents provide evidence for the existence of the patient and substantiate the integrity of the data collected. Source documents are filed at the investigator's site.

Data reported on the CRFs or entered in the eCRFs that are transcribed from source documents must be consistent with the source documents or the discrepancies must be

explained. The investigator may need to request previous medical records or transfer records, depending on the trial; also current medical records must be available.

For eCRFs all data must be derived from source documents.

For paper CRFs the following data need to be derived from source documents:

- Patient identification (gender, date of birth)
- Patient participation in the trial (substance, trial number, patient number, date patient was informed)
- Dates of Patient's visits, including dispensing of trial medication
- Medical history (including trial indication and concomitant diseases, if applicable)
- Medication history
- Adverse events (onset date (mandatory), and end date (if available))
- Serious adverse events (onset date (mandatory), and end date (if available))
- Concomitant therapy (start date, changes)
- Originals or copies of laboratory results (in validated electronic format, if available)
- Conclusion of Patient's Participation in the trial

8.2.5 Direct access to source data - documents

The investigator / institution will permit trial-related monitoring, audits, IRB / IEC review and regulatory inspection, providing direct access to all related source data / documents. CRFs/eCRFs and all source documents, including progress notes and copies of laboratory and medical test results must be available at all times for review by the sponsor's clinical trial monitor, auditor and inspection by health authorities (e.g., FDA). The Clinical Research Associate (CRA) / on site monitor and auditor may review all CRFs/eCRFs, and written informed consents. The accuracy of the data will be verified by reviewing the documents described in Section 8.2.4.

8.3 QUALITY ASSURANCE AUDIT

A quality assurance audit of this trial may be conducted by the sponsor or sponsor's designees. The quality assurance auditor will have access to all medical records, the investigator's trial-related files and correspondence, and the informed consent documentation of this clinical trial.

8.4 PROCEDURES

8.4.1 Adverse events

An adverse event (AE) is defined as any untoward medical occurrence, including an exacerbation of a pre-existing condition, in a patient in a clinical investigation who received a pharmaceutical product. The event does not necessarily have to have a causal relationship with this treatment.

All adverse events occurring during the course of the clinical trial (i.e., from signing the informed consent onwards through the observational phase) will be collected, documented and reported to the sponsor by the investigator according to the specific definitions and instructions detailed in the 'Adverse Event Reporting' section of the Investigator Site File.

A serious adverse event (SAE) is defined as any AE which results in death, is immediately life-threatening, results in persistent or significant disability / incapacity, requires or prolongs patient hospitalisation, is a congenital anomaly / birth defect, or is to be deemed serious for any other reason if it is an important medical event when based upon appropriate medical judgement which may jeopardise the patient and may require medical or surgical intervention to prevent one of the other outcomes listed in the above definitions.

All adverse events, serious and non-serious, will be fully documented on the appropriate CRF(s) / eCRFs. For each adverse event, the investigator will provide the onset, end, intensity, treatment required, outcome, seriousness and action taken with the investigational drug. The investigator will determine the relationship of the investigational drug to all AEs as defined in the 'Adverse Event Reporting' Section of the Investigator Site File.

The basis for judging the intensity of the AE as well as the causal relationship between the investigational product and the AE is described below.

Intensity of event

- Mild: Awareness of sign(s) or symptom(s) which is/are easily tolerated
- Moderate: Enough discomfort to cause interference with usual activity
- Severe: Incapacitating or causing inability to work or to perform usual activities

Causal relationship

Medical judgment should be used to determine the relationship, considering all relevant factors, including pattern of reaction, temporal relationship, de-challenge or re-challenge, confounding factors such as concomitant medication, concomitant diseases and relevant history. Assessment of causal relationship should be recorded in the case report forms.

- Yes: There is a reasonable causal relationship between the investigational drug administered and the AE.
- No: There is no reasonable causal relationship between the investigational drug administered and the AE.

If a SAE is reported from a still blinded trial, the causal relationship must be provided by the investigator for all potential trial drugs, i.e. the BI trial drug and for all other trial drugs (i.e., any active comparator or placebo according to the trial design).

The investigator has the obligation to report AEs during the specified observational phase. If defined in the CTP, the investigator also has the responsibility to report AEs occurring in a certain period after a patient completes the trial. Any AEs reported to the sponsor during this phase must be documented in the safety database.

If not stipulated differently in the ISF, SAEs are to be reported to the sponsor using the BI Serious Adverse Event Report Form including a documented causal relationship assessment and providing as much detail regarding the SAE as possible. With receipt of follow-up information, all remaining fields on the SAE form are to be completed or updated.

Any serious or significant AE, whether or not considered related to the investigational product, and whether or not the investigational product has been administered, must be reported immediately by telephone / fax to the sponsor. Expedited reporting of serious adverse events, e.g., suspected unexpected serious adverse reactions (SUSARs), will be done according to local regulatory requirements. Further details regarding this reporting procedure are provided in the ISF.

Following every such telephone / fax report, the Clinical Monitor must provide a written report of the serious or significant AE and any sequelae to Corporate Drug Safety according to the appropriate Corporate SOP(s). These narratives, which confirm the information collected by telephone, may give additional information not available at the time of the initial report.

8.4.2 Emergency procedures

Not applicable.

8.5 RULES FOR AMENDING PROTOCOL

All CTP amendments must be documented, dated and signed by all signatories (or their successors) of the original protocol. This also applies to any local amendment that may become necessary. Amendments (excluding those exclusively for administrative or logistical changes) need to be submitted for review/approval to the IRB / IEC, the CA in each participating EU member state, and any other regulatory authorities as required by local laws and regulations. Local Amendments will only be submitted in the countries / centres concerned.)

8.6 DISCONTINUATION OF THE TRIAL BY THE SPONSOR

Boehringer Ingelheim reserves the right to discontinue the trial at any time for the following reasons:

1. Failure to meet expected enrolment goals overall or at a particular trial site,
2. Emergence of any efficacy/safety information that could significantly affect continuation of the trial,
3. Violation of GCP, the CTP, or the contract by a trial site or investigator, disturbing the appropriate conduct of the trial.

The investigator / the trial site will be reimbursed for reasonable expenses incurred in case of trial termination (except in case of the third reason).

REV A

8.7 STATEMENT OF CONFIDENTIALITY

Individual patient medical information obtained as a result of this trial is considered confidential and disclosure to third parties is prohibited with the exceptions noted below. Patient confidentiality will be ensured by using patient identification code numbers.

Treatment data may be given to the patient's personal physician or to other appropriate medical personnel responsible for the patient's welfare. Data generated as a result of the trial need to be available for inspection on request by the participating physicians, the sponsor's representatives, by the *IRB / IEC* and the regulatory authorities, *i.e. the FDA*.

8.8 PUBLICATION POLICY

Boehringer Ingelheim is as much as possible dedicated to support process of free exchange of relevant scientific information. Any publication of the result of this trial must be consistent with the Boehringer Ingelheim publication policy. The rights of the investigator and of the sponsor with regard to publication of the results of this trial are described in the investigator contract. As a general rule, no trial results should be published prior to finalisation of the Clinical Trial Report (CTR).

8.9 COMPLETION OF TRIAL

The EC/competent authority in each participating EU member state needs to be notified about the end of the trial (last patient/patient out, unless specified differently in Section 6.2.3 of the CTP) or early termination of the trial.

REV A

9. REFERENCES

9.1 PUBLISHED REFERENCES

P89-1237 Weintraub SJ, Eschenbacher WL. The inhaled bronchodilators ipratropium bromide and metaproterenol in adults with CF. Chest 1989;95(4):861-864.

P92-1920 Sanchez I, Holbrow J, Chernick. Acute bronchodilator response to a combination of beta-adrenergic and anticholinergic agents in patients with cystic fibrosis. J Pediatr 1992;120(3):486-488.

P92-6329 Avital A, Sanchez I, Chernick V. Efficacy of salbutamol and ipratropium bromide in decreasing bronchial hyperreactivity in children with cystic fibrosis. Pediatr Pulmonol 1992;13(1):34-37.

P96-1115 Cropp GJ. Effectiveness of bronchodilators in cystic fibrosis. Am J Med 1996;100(1a):19s-29s.

P04-10376 Colombo JL. Long-acting bronchodilators in cystic fibrosis. Curr Opin Pulm Med 2003;9(6):504-508.

P05-12782 Miller MR, Hankinson J, Brusasco V, Burgos F, Casaburi R, Coates A, et al. Standardisation of spirometry. Eur Respir J 2005;26(2):319 – 338.

R98-1298 Knudson RJ, Lebowitz MD, Holberg CJ, Burrows B. Changes in the normal maximal expiratory flow-volume curve with growth and aging. Am Rev Respir Dis 1983;127:725-734.

R01-1277 Davis PB, Drumm M, Konstan MW. Cystic fibrosis. Am J Respir Crit Care Med 1996;154:1229-1256.

R02-1107 Fuchs HJ, Borowitz DS, Christiansen DH, Morris EM, et al. Effect of aerosolized recombinant human DNase on exacerbations of respiratory symptoms and on pulmonary function in patients with cystic fibrosis. N Engl J Med 1994;331(10):637-42.

R02-1108 Rosenfeld M, Emerson J, Williams-Warren J, Pepe M, Smith A, Montgomery B, et al. Defining a pulmonary exacerbation in cystic fibrosis. J Pediatr 2001;139(3):359-65.

R02-0874 Wang X, Dockery DW, Wypij D,Fay ME, Ferris BG. Pulmonary function between 6 and 18 years of age. Pediatr Pulmonol 1993;15:75-88.

R05-1407 Boucher RC. Human airway ion transport, part 2. Am J Respir Crit Care Med 1994;150(2):581-593.

REVA

R05-1456 Konstan MW, Butler SM, Schidlow DV, Morgan WJ, Julius JR, Johnson CA. Patterns of medical practice in cystic fibrosis: Part II. Use of therapies. Pediatr Pulmonol 1999;28:248-254.

R08-1121 Wanger J, Clausen JL, Coates A, Pedersen OF, Brusasco V, Burgos F, et al. Standardisation of the measurement of lung volumes. Eur Respir J 2005;26:511-522.

REV A

9.2 UNPUBLISHED REFERENCES

U04-3343 Van Andel AE, Korducki L, Leon M. A randomized, double-blind, double-dummy, placebo- and active-controlled, parallel group efficacy and safety comparison of 12-week treatment of two doses [5 µg (2 actuations of 2.5 µg) and 10 µg (2 actuations of 5 µg)] of tiotropium inhalation solution delivered by the Respimat inhaler, placebo and ipratropium bromide inhalation aerosol (MDI) in patients with chronic obstructive pulmonary disease (COPD). Unpublished report (205.252). 25 August 2004.

U04-3400 Van Andel AE, Korducki L. A randomized, double-blind, double-dummy, placebo- and active-controlled, parallel group efficacy and safety comparison of 12-week treatment of two doses [5 µg (2 actuations of 2.5 µg) and 10 µg (2 actuations of 5 µg)] of tiotropium inhalation solution delivered by the Respimat inhaler, placebo and ipratropium bromide inhalation aerosol (MDI) in patients with chronic obstructive pulmonary disease (COPD). Unpublished report (205.251). 16 September 2004.

U07-3355 Koker P, Bhattacharya S, Staab A. A randomized, double-blind, placebo-controlled study to investigate the efficacy and safety of 24 weeks of oral treatment with BIIL 284 BS in adult (75 mg, 150 mg)and pediatric (75 mg) cystic fibrosis patients. Unpublished report (543.45). 07 September 2007.

U08-3223-01 Koker P. Investigator's Brochure. Version 1, (Report in Progress).

REV A

10. APPENDICES

APPENDIX 10.1 RESPIRATORY AND SYSTEMIC SYSTEMS QUESTIONNAIRE (RSSQ)

Introduction: key points

1. The respiratory and systemic symptoms questionnaire is to be administered to the patient (with the help of a parent for patients aged 6 – 12 years) by the study coordinator (termed "interviewer" in the questionnaire) at the beginning of each visit. Every effort should be made to ensure that the same person administers the questionnaire to the patient at all visits.

2. The questionnaire is intended to gather information from the patient about the types of symptoms that the patient has experienced since the last study-related visit, including (but not restricted to) any symptoms the patient is experiencing on the actual day of the interview.

3. We are interested in obtaining information about abrupt, acute, or sudden changes in the patient's symptoms which are noticeably greater (in the view of the patient) than day to day fluctuations in these symptoms normally experienced by the patient. It is very important that patients understand that they are **not** being asked to indicate changes relative to the pre-treatment baseline. It is also very important that patients understand that they are being asked to describe symptoms that have occurred *since* the last study-related visit, but that they are **not** being asked to compare these symptoms with the descriptions they provided at the last study-related visit.

4. The questionnaire asks about a number of symptoms that patients' are likely to experience routinely as part of their normal "stable" condition, but there may also be symptoms which a patient does not normally experience from day to day, or which they have never experienced at all (e.g. coughing up of blood). It is important for patients to appreciate this, and to understand that they should indicate if they have never experienced a particular symptom. It may also be necessary to clarify that a symptom that the patient has noticed since the last visit but does not normally experience on a day to day basis does constitute a change in that symptom (e.g. patient does not normally have a cough, but has been coughing since the last visit).

5. It is important for patients to understand that there are no right or wrong answers, and that they may ask for clarification if they do not understand certain words or phrases, or if they do not understand the question in general.

6. Any clarifications that are provided to the patient should not have any direction attached or implied. For example, clarifiers such as "more or less" or "thicker or thinner" should be used rather than "more" or "thicker".

REV A

7. In order to ensure that patients continue to describe changes in their symptoms relative to their normal day to day fluctuations, it is important to remind the patient of this as the interview proceeds; specific reminders have been placed at appropriate points within the questionnaire.

8. For each symptom, the interviewer will begin by saying the words in quotation marks to the patient ***verbatim.*** A less structured interaction between the patient and the interviewer will then take place, as the patient provides an initial response and the interviewer provides further clarification, if necessary. Once the interviewer is confident that the patient understands what is being asked and the patient has provided information as to whether the symptom has increased, decreased or not changed, the interviewer will then ask the patient to specify the magnitude of change using the specific response choices provided, even if the patient has already provided information about the magnitude of the change – in order to ensure consistency across patients and sites, it is very important that the patient rates the magnitude of the change using the response choices provided. It is not necessary to provide all the response choices, e.g. if the patient has indicated that the symptom has increased, then only the response choices related to an increase in the symptom need to be provided to the patient.

<u>Example</u>

Interviewer: **“**The first question deals with changes in your sputum production. Another word for sputum that you may be more familiar with is mucus. Tell me about any changes in the amount of sputum you have been coughing up since your last visit.”

Patient: “I have noticed that I have been coughing up a lot more sputum in the last couple of weeks than I normally do”

Interviewer: “Compared with the amount of sputum you normally cough up, would you say that you have been coughing up **much more** or **a little more**?

Patient: “I would say it has been **much more**”

9. For item 2.2 “Sputum color”, the interviewer will ***synthesize*** the patient’s comments and the **interviewer** will complete the response choice (<u>**not**</u> the patient) based on the criteria provided in the interviewer notes.

10. For item 12 “Change in sinus discharge”, the interviewer will ***synthesize*** the patient’s comments and the **interviewer** will complete the response choice, <u>**not**</u> the patient. This is because the question has more than 1 dimension (i.e. color, thickness and amount of sinus discharge).

INTERVIEW SCRIPT

Please note that text in quotation marks is to be provided to the patient verbatim.

"In the next few minutes, I will be asking you a number of questions about how you have been feeling since your last study-related visit. This does include how you are feeling today, but is not limited to today only. In order to find out how you have been feeling, I will be asking you about specific symptoms that you may or may not have experienced. You are likely to be familiar with some of the symptoms I ask you about, but there may also be other symptoms that you have never experienced. If this is the case, it is O.K. to let me know that you have never experienced that symptom. If you are not sure how to answer a question, please let me know and I will try to give you some more help in understanding the question.

When you are answering the questions, I would like you to think back over the time since your last study-related visit, and let me know whether the symptom I am asking about is clearly different compared with what you are normally used to on a day to day basis. We are interested in your own impressions of these symptoms, so there are no right or wrong answers to these questions. If you would like me to clarify any of the words or phrases within the questions, please let me know."

1. Increased sputum production

Interviewer: "The first question deals with changes in your sputum production. Another word for sputum that you may be more familiar with is mucus. Tell me about any changes in the amount of sputum you have been coughing up since your last visit."

The following response choices will be used:

much more	a little more	no change	a little less	much less

Notes for interviewer:

- If patient asks 'changes relative to what?' clarify that we are interested in changes beyond what the patient considers "normal" on a day to day basis.

REV A

2. Change in sputum appearance

2.1 sputum thickness

Interviewer: "Tell me about any changes in the thickness of your sputum since your last visit."

The following response choices will be used:

much thicker	a little thicker	no change	a little thinner	much thinner

2.2 sputum color

Interviewer: "Tell me about any changes in the color of your sputum since your last visit."

The interviewer will synthesize the patient's comments and complete the following response choice:

worse	no change	better

Notes to interviewer:

- ***The interviewer will be provided with a color scale to assist in the description of color changes***
- ***"worse" is in the direction "clear → yellow → green → brown"***
- ***"better" is in the direction "brown → green → yellow → clear"***

REV A

3. Increased chest congestion

Interviewer: "I am now going to ask you a question about chest congestion. By chest congestion, I mean the feeling you get in your chest when you have mucus in your lungs. Tell me about any changes in your chest congestion since your last visit."

The following response choices will be used:

large increase	a little increase	no change	a little decrease	large decrease

4. New or increased coughing up of blood

Interviewer: "Tell me whether you have been coughing up any blood since your last visit and if so, how the amount of blood you have been coughing up compares with what you are normally used to?"

The following response choices will be used:

large increase	a little increase	no change	a little decrease	large decrease

REV A

5. Increased cough

5.1 intensity of cough

Interviewer: "Tell me about any changes in how hard you have been coughing since your last visit."

The following response choices will be used:

much harder	a little harder	no change	a little lighter	much lighter

5.2 frequency of cough

Interviewer: "Tell me about any changes in how often you have been coughing since your last visit."

The following response choices will be used:

much more often	a little more often	no change	a little less often	much less often

Reminder to Patient

Interviewer: "We are now about one-third of the way through the interview. Thank you for your help so far. As a reminder, for the next questions you should still be considering whether you have noticed any changes in the symptoms that are clearly different from what you are normally used to experiencing on a day to day basis."

REV A

6. Decreased exercise tolerance

Interviewer: "For this next question, I will be asking you about your ability to perform daily activities. By daily activities, I mean any activities that you would normally do as part of your regular day to day routine. Examples of this are climbing stairs, walking to school, playing sports (if this is part of your normal routine) or doing chores around the house. Tell me about any changes in your ability to perform these types of activities since your last visit."

The following response choices will be used:

much harder	a little harder	no change	a little easier	much easier

Notes to interviewer:
– It is important that the patient understands that "activities" includes all types of activities performed routinely, and is not restricted to "organized" activities such as sporting events or exercise programs

7. Increased dyspnea with exertion

Interviewer: "Tell me about any changes in your breathing when performing daily activities since your last visit."

The following response choices will be used:

much more difficult	a little more difficult	no change	a little easier	much easier

Notes to interviewer:
– It is possible that the patient will indicate that their breathing has changed when answering question 6 ("exercise tolerance"). If so, the interviewer may simply ask the patient for confirmation in order to complete the symptom checklist for dyspnea with exertion

REV A

REV A

8. Malaise, fatigue or lethargy

Interviewer: "Tell me about any changes in your energy level since your last visit."

The following response choices will be used:

much more tired	a little more tired	no change	a little more energy	much more energy

9. Fever

Interviewer: "Have you had a fever since your last visit?"

This should be a "yes" or "no" answer; complete symptoms checklist and move to next question.

10. Weight loss

Interviewer: "Tell me about any changes in your weight since your last visit."

The following response choices will be used:

large weight gain	a little weight gain	no change	a little weight loss	large weight loss

Notes to interviewer:

– Patient may ask 'how much of a change?' with respect to changes in weight. If so, interviewer can reiterate that we are interested in changes that are over and above normal daily variations in weight

Reminder to Patient

Interviewer: "We are getting close to the end of the interview. As a final reminder, for the last few questions, we still want you to consider whether you have noticed any changes in the symptoms that are clearly different from what you are normally used to experiencing on a day to day basis."

11. Sinus pain and tenderness

Notes to interviewer:
- An illustration of the face will be provided to allow the interviewer to clarify the location of the sinuses (maxillary and frontal)

Interviewer: "Have you had any pain or tenderness in your sinuses?"

This should be a "yes" or "no" answer; complete symptoms checklist and move to next question.

12. Change in sinus discharge

Interviewer: "Tell me about any changes in your sinus discharge since your last visit?"

The interviewer will synthesize the patient's comments and complete the following response choice:

worse	no change	better

Notes to interviewer:
- A clarifier may be needed for the term "sinus discharge", especially with young children. The clarifier to be used is "what your mucus is like when you blow your nose".
- Interviewer may need to clarify that we are interested in changes related to color, thickness, and amount of sinus discharge
- "worse" would be a change in color in the direction "clear → yellow → green → brown", and / or increase in thickness, and / or increase in amount of discharge
- "better" would be a change in color in the direction "brown → green → yellow → clear", and / or decrease in thickness, and / or decrease in amount of discharge

REV A

13. <u>School or work absenteeism (due to illness)</u>

**Notes for interviewer:**
– If the interview is conducted in a non-school (non-work) period (e.g. summer), or if the patient is unemployed, the question should be rephrased accordingly ("Since your last visit, have you missed any scheduled activities due to illness?")

Interviewer: "Since your last visit, have you missed any school or work due to illness?"

– If patient answers "no", complete symptoms checklist and move to next question.

– If patient answers "yes" and describes the reason for the absence, complete symptoms checklist by describing reason and move to next question.

– If patient answers "yes" but does not describe the reason for the absence, ask the following question:

Interviewer: "What was the illness that made you miss school or work?"

**Notes for interviewer:**
– Interviewer will record specific illness in symptom checklist (e.g. flu, broken leg, trouble breathing etc.)<br>– May need some examples of 'scheduled activities' (e.g. 'going outside to play', 'going to camp' etc.)

14. <u>Decreased appetite</u>

Interviewer: "Tell me about any changes in your appetite since your last visit."

The following response choices will be used:

large increase	a little increase	no change	a little decrease	large decrease

REV A

APPENDIX 10.2 PREDICTED EQUATIONS FOR PULMONARY FUNCTION

****NOTE: The predicted equations are being provided as an appendix for documentation purposes; they are not intended for use by individual sites****

Predicted equations for FVC, FEV_1 and $FEF_{25\text{-}75}$: white boys aged 6-18 years [from Wang et al [R02-1109]]

Age (years)	LnFVC		$LnFEV_1$		$LnFEF_{25\text{-}75}$	
	α	β	α	β	α	β
6	-0.024	2.470	-0.109	2.252	**	
7	-0.018	2.489	-0.104	2.270	**	
8	0.005	2.443	-0.089	2.257	0.264	1.505
9	0.017	2.426	-0.063	2.197	0.308	1.443
10	0.030	2.407	-0.057	2.212	0.290	1.557
11	0.009	2.468	-0.093	2.324	0.242	1.738
12	-0.061	2.649	-0.161	2.512	0.165	1.982
13	-0.175	2.924	-0.292	2.843	0.007	2.396
14	-0.219	3.060	-0.329	2.983	0.014	2.483
15	-0.079	2.859	-0.141	2.709	0.241	2.163
16	0.104	2.591	0.062	2.409	0.503	1.764
17	0.253	2.374	0.262	2.099	0.762	1.368
18	0.296	2.316	0.251	2.129	0.678	1.528

Model: $Ln(PF) = \alpha + \beta Ln(Ht)$
Where,
PF is FVC (L), FEV_1 (L) or $FEF_{25\text{-}75\%}$ (L/s), and Ht is height (m).

** not available: use equations from Knudson et al [R98-1298]

REVA

Predicted equations for FVC, FEV_1 and FEF_{25-75}: white girls aged 6-18 years [from Wang et al [R02-1109]]

Age (years)	LnFVC		$LnFEV_1$		$LnFEF_{25-75\%}$	
	α	β	α	β	α	β
6	-0.013	2.007	-0.109	1.949	**	
7	-0.062	2.385	-0.144	2.243	**	
8	-0.055	2.381	-0.137	2.239	0.247	1.668
9	-0.039	2.351	-0.123	2.222	0.254	1.710
10	-0.068	2.458	-0.161	2.364	0.195	1.933
11	-0.120	2.617	-0.223	2.558	0.161	2.091
12	-0.174	2.776	-0.264	2.709	0.185	2.120
13	-0.061	2.576	-0.153	2.535	0.294	1.976
14	0.139	2.208	0.046	2.178	0.450	1.711
15	0.210	2.099	0.148	2.008	0.581	1.486
16	0.226	2.097	0.181	1.972	0.654	1.366
17	0.214	2.146	0.176	1.992	0.688	1.290
18	0.195	2.179	0.152	2.031	0.520	1.622

Model: $Ln(PF) = \alpha + \beta Ln(Ht)$

Where,

PF is FVC (L), FEV_1 (L) or $FEF_{25-75\%}$ (L/s), and Ht is height (m).

** not available: use equations from Knudson et al [R98-1298]

REV A

Predicted equations for FVC, FEV_1 and FEF_{25-75} : black boys aged 6-18 years [from Wang et al [R02-1109]]

Age (years)	LnFVC		$LnFEV_1$		$LnFEF_{25-75}$	
	α	β	α	β	α	β
6	-0.088	1.961	-0.166	1.723	**	
7	-0.040	2.040	-0.122	1.846	**	
8	-0.094	2.323	-0.225	2.271	0.097	1.544
9	-0.074	2.308	-0.142	2.059	0.255	1.248
10	-0.110	2.417	-0.157	2.117	0.230	1.428
11	-0.138	2.453	-0.176	2.166	0.256	1.438
12	-0.224	2.710	-0.307	2.548	0.085	1.936
13	-0.342	2.975	-0.486	2.962	-0.121	2.476
14	-0.337	3.035	-0.472	3.010	-0.115	2.536
15	-0.226	2.889	-0.318	2.789	0.170	2.120
16	0.058	2.425	0.074	2.140	0.663	1.299
17	0.148	2.310	0.053	2.223	0.505	1.618
18	0.152	2.341	0.130	2.121	0.859	1.053

Model: $Ln(PF) = \alpha + \beta Ln (Ht)$
Where,
PF is FVC (L), FEV_1 (L) or $FEF_{25-75\%}$ (L/s) and Ht is height (m).

** not available: use equations from Knudson et al [R98-1298]

REV A

Predicted equations for FVC, FEV_1 and FEF_{25-75}: black girls aged 6-18 years [from Wang et al [R02-1109]]

Age (years)	LnFVC		$LnFEV_1$		$LnFEF_{25-75\%}$	
	α	β	α	β	α	β
6	-0.172	2.117	-0.288	2.182	**	
7	-0.135	2.132	-0.250	2.158	**	
8	-0.176	2.362	-0.276	2.295	-0.283	2.990
9	-0.200	2.452	-0.294	2.330	0.025	2.062
10	-0.230	2.571	-0.344	2.507	0.051	2.028
11	-0.204	2.526	-0.308	2.460	0.078	2.006
12	-0.107	2.342	-0.219	2.312	0.225	1.804
13	-0.042	2.294	-0.117	2.196	0.418	1.504
14	0.105	2.021	0.041	1.920	0.574	1.257
15	0.253	1.787	0.203	1.662	0.599	1.281
16	0.111	2.098	0.129	1.824	0.653	1.175
17	0.205	1.930	0.273	1.547	0.713	1.067
18	-0.042	2.423	-0.084	2.259	-0.209	2.896

Model: $Ln(PF) = \alpha + \beta Ln\ (Ht)$
Where,
PF is FVC (L), FEV_1 (L) or $FEF_{25-75\%}$ (L/s) and Ht is height (m).

** not available: use equations from Knudson et al [R98-1298]

REV A

Predicted equations for FVC, FEV_1 and FEF_{25-75}: males and females, 19 years of age and older [from Knudson et al [R98-1298]]

		Coefficients	
	Constant	Height (cm)	Age (yr)
**Males: 19 to 24 years**			
**FEV_1**	-6.1181	0.0519	0.0636
**FVC**	-6.8865	0.0590	0.0739
**FEF_{25-75}**	-6.1990	0.0539	0.0749
**Males: ≥ 25 years**			
**FEV_1**	-6.5147	0.0665	-0.0292
**FVC**	-8.7818	0.0844	-0.0298
**FEF_{25-75}**	-4.5175	0.0579	-0.0363
**Females: 19 years**			
**FEV_1**	-3.7622	0.0351	0.0694
**FVC**	-4.4470	0.0416	0.0699
**FEF_{25-75}**	-2.8007	0.0279	0.1275
**Females ≥ 20 years**			
**FEV_1**	-1.8210	0.0332	-0.0190
**FVC**	-3.1947	0.0444	-0.0169
**FEF_{25-75}**	-0.4057	0.0300	-0.0309

Predicted value = constant + [height coefficient x height (cm)] + [age coefficient x age (yr)]

REVA

APPENDIX 10.3 USE OF THE RESPIMAT DEVICE

Instructions for Use

Respimat® inhaler

How to use your RESPIMAT® inhaler

This leaflet explains how to use and care for your RESPIMAT® inhaler. **Please read and carefully follow these instructions.**

The RESPIMAT® inhaler releases medication slowly and gently, making it easy to inhale it into your lungs.

The RESPIMAT® inhaler enables you to inhale the medicine contained in a cartridge. **You will need to use this inhaler only ONCE A DAY. Each time you use it take 2 PUFFS.** In the box you will find the RESPIMAT® inhaler and the RESPIMAT® cartridge. Before the RESPIMAT ® inhaler is used for the first time, the cartridge provided must be inserted.

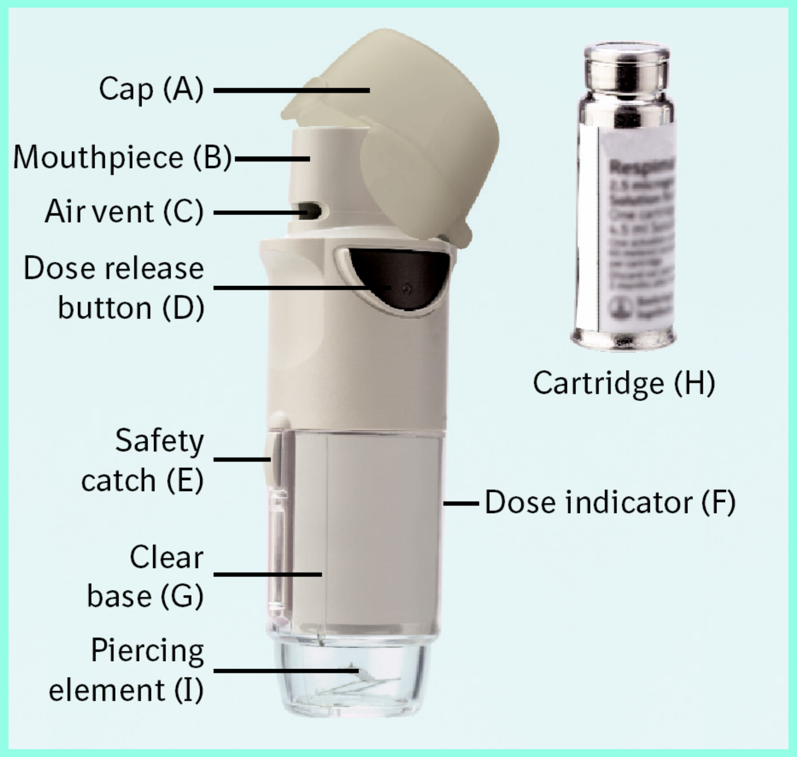

RESPIMAT® inhaler and the RESPIMAT® cartridge

Inserting the cartridge and preparation for use

The following steps 1-6 are necessary before first use:

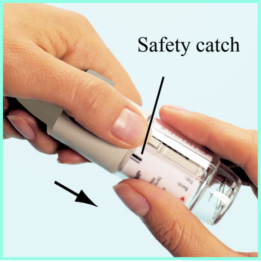 1	**1** With the grey cap closed, press the safety catch (E) and pull off the clear base (G).
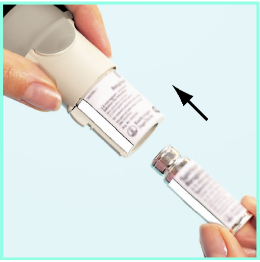 2a	**2** Take the cartridge (H) out of the box. Push the **narrow** end of the cartridge into the inhaler until it clicks into place (2a). The cartridge should be pushed **gently** against a firm surface to ensure that it has gone all the way in (2b).<br><br>Do not remove the cartridge once it has been inserted into the inhaler.
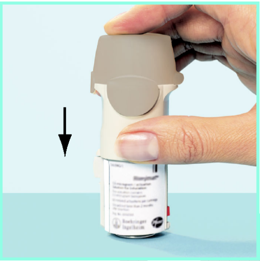 2b	
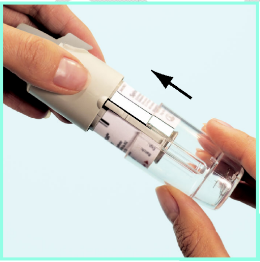 3	**3** Replace the clear base (G).<br><br>Do not remove the clear base again.

To prepare the RESPIMAT® inhaler for first-time use

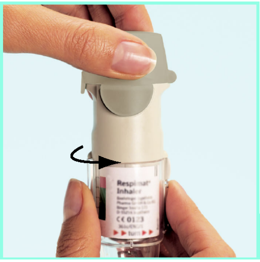 4	**4**	Hold RESPIMAT® inhaler upright, with the grey cap (A) closed. Turn the clear base (G) in the direction of the red arrows on the label until it clicks (half a turn).
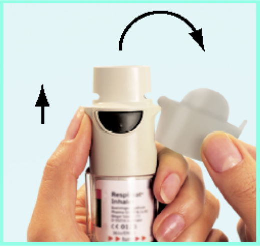 5	**5**	Open the grey cap (A) until it snaps fully open.
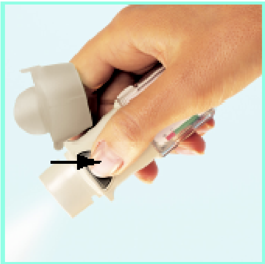 6	**6**	Point the RESPIMAT® inhaler towards the ground.<br>Press the dose release button (D). Close the grey cap (A).<br><br>**Repeat steps 4, 5 and 6 until a cloud is visible.**<br><br>**Then repeat steps 4, 5 and 6 three more times to ensure the inhaler is prepared for use.**<br><br>**Your RESPIMAT® inhaler is now ready to use.**<br><br>These steps will not affect the number of doses available. After preparation your RESPIMAT® inhaler will be able to deliver 60 puffs.

REV A

REV A

Using the RESPIMAT® inhaler

You will need to use this inhaler only ONCE A DAY.
Each time you use it take 2 PUFFS.

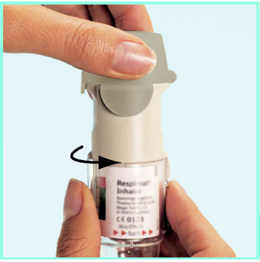 I	**I** Hold RESPIMAT® inhaler upright, with the grey cap (A) closed, to avoid accidental release of dose. Turn the clear base (G) in the direction of the red arrows on the label until it clicks (half a turn).
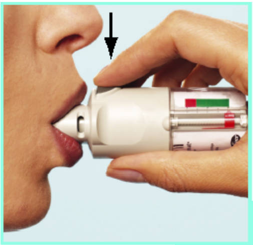 II	**II** Open the grey cap (A) until it snaps fully open. Breathe out slowly and fully, and then close your lips around the end of the mouthpiece without covering the air vents (C). Point your RESPIMAT® inhaler to the back of your throat.<br>While taking in a slow, deep breath through your mouth, press the dose release button (D) and continue to breathe in slowly for as long as you can. Hold your breath for 10 seconds or for as long as comfortable.<br><br>**III Repeat steps I and II so that you get the full dose.**<br><br>**You will need to use this inhaler only ONCE A DAY.**<br><br>**Close the grey cap until you use your RESPIMAT® inhaler again.**<br><br>If the RESPIMAT® inhaler has not been used for more than 3 days release one puff towards the ground. If the RESPIMAT® inhaler has not been used for more than 21 days repeat steps 4 to 6 until a cloud is visible. Then repeat steps 4 to 6 three more times.

When to get a new RESPIMAT® inhaler

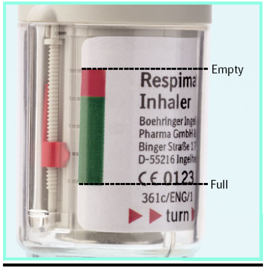	The RESPIMAT® inhaler contains 60 puffs (30 doses). The dose indicator shows approximately how many doses are left. When the pointer enters the red area of the scale, there is, approximately, medication for 14 puffs (7 days) left.<br><br>Once the dose indicator has reached the end of the red scale (i.e. all 60 doses have been used), the RESPIMAT® inhaler is empty and locks automatically. At this point, the base cannot be turned any further.

What if...

What if...	Reason	What to do
I can't turn the base easily.	a) The RESPIMAT® inhaler is already prepared and ready to use.<br><br>b) The RESPIMAT® inhaler is locked after 60 puffs (30 doses).	a) The RESPIMAT® inhaler can be used as it is.<br><br>b) Prepare and use your new RESPIMAT® inhaler.
I can't press the dose release button.	The clear base has not been turned.	Turn the clear base until it **clicks**. (half a turn)
The clear base springs back after I have turned it.	The clear base was not turned far enough.	Prepare the RESPIMAT® inhaler for use by turning the clear base until it **clicks**. (half a turn)
I can turn the clear base past the point where it clicks.	Either the dose release button has been pressed, or the clear base has been turned too far.	With the grey cap closed, turn the base until it **clicks**. (half a turn)

How to care for your inhaler

Clean the mouthpiece including the metal part inside the mouthpiece with a damp cloth or tissue only, at least once a week.

Any minor discoloration in the mouthpiece does not affect the performance of your RESPIMAT® inhaler.

If necessary, wipe the outside of your RESPIMAT® inhaler with a damp cloth.

Further information

The RESPIMAT® inhaler must not be disassembled after inserting the cartridge and replacing the clear base.

Do not touch the piercing element inside the base.

Keep out of the reach and sight of children.

Do not freeze.

Boehringer Ingelheim Pharma GmbH & Co. KG
D - 55216 Ingelheim
Germany

CE 0123

HI-Master-Version-Respimat-20061130

APPENDIX 10.4 USE OF AN MDI

1. Hold canister as illustrated in figure below and shake well before each use.

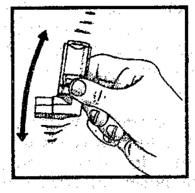

2. Exhale deeply through mouth. Enclose mouthpiece with the lips. The base of the canister should be held vertically. Keep the eyes closed because temporary blurring of vision may result if the aerosol is sprayed into the eyes.

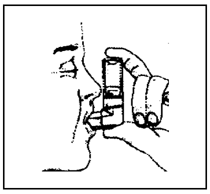

3. Inhale slowly through the mouth and at the same time firmly press once on the upended canister base; continue to inhale deeply.

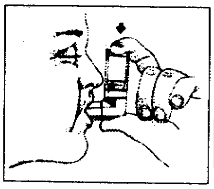

4. Hold your breath for ten seconds and then remove the mouthpiece from the mouth and exhale slowly. Wait approximately fifteen seconds, shake the inhaler again and repeat second inhalation as outlined above.

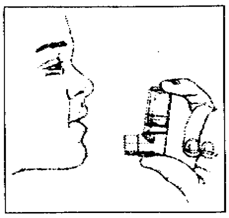

Keep the mouthpiece clean and free of debris.

Discontinue use of the MDI after you have used greater than 200 inhalations or used for more than 25 days.

REV A

APPENDIX 10.5 PHARMACOKINETIC METHODS

10.5.1 Planned analyses for pharmacokinetic evaluations

Concentrations will be used for calculations in the format that is reported in the bioanalytical report. The data format for descriptive statistics of concentrations will be identical with the data format of the respective concentrations. For the calculation of pharmacokinetic parameters, only concentrations within the validated concentration range will be used. The actual sampling times will be used for the evaluation of plasma concentrations. If the actual sampling time was not recorded or is missing for a certain time point, the planned time should generally be used for this time point instead.

For pre-dose samples, the actual sampling time will be set to zero. Noncompartmental pharmacokinetic parameters will be determined using the WinNonlin™ software program (Professional, version 4.1 or higher, Pharsight Corporation, Mountain View, California) or another validated program.

The following descriptive statistics will be calculated for analyte concentrations as well as for all primary and secondary pharmacokinetic parameters: N, arithmetic mean, standard deviation, minimum, median, maximum, arithmetic coefficient of variation, geometric mean, and geometric coefficient of variation. The descriptive statistics of pharmacokinetic parameters will be calculated using the individual values with the number of decimal places as provided by the evaluation program. Then the individual values as well as the descriptive statistics will be reported with three significant digits in the clinical trial report. Plasma concentrations will be plotted graphically versus time for all patients as listed in the drug plasma concentration-time tables. For the presentation of the mean profiles, the arithmetic mean and the planned blood sampling times will be used.

10.5.2 Handling of missing data

Drug concentration-time profiles:

Concentration data identified with NOS (no sample), NOR (no valid result), NOA (not analyzed), BLQ (below the limit of quantification) and NOP (no peak detectable) will be ignored and not replaced by zero at any time point (including the lag phase). Descriptive statistics of concentrations at specific time points will be calculated only when at least 2/3 of the individuals have concentrations within the validated concentration range. The overall sample size to decide whether the '2/3' rule is fulfilled will be based on the total number of samples intended to be drawn for that time point (i.e. BLQ, NOR, NOS, NOA, NOP are included).

Pharmacokinetic parameters:

In the non-compartmental analysis, concentration data identified with NOS, NOR and NOA will not be considered. BLQ and NOP values in the lag-phase will be set to zero. The lag phase is defined as the period between time 0 and the first time point with a concentration above the quantification limit. All other BLQ/NOP values of the profile will be ignored. If

REV A

the pre-dose plasma concentration is less than or equal to 5% of Cmax value in that subject, the subject's data without any adjustments can be included in all pharmacokinetic measurements and calculations (i.e. the pre-dose value will not be changed to zero). If the pre-dose value is greater than 5% of Cmax, the subject should be dropped from all statistical evaluations. The individual pharmacokinetic parameters can be calculated and listed separately. Every effort will be made to include all concentration data in an analysis. If a concentration value is excluded from all calculations, it will not be presented graphically or used for the calculation of descriptive statistics and parameter determination. However the excluded concentration itself will be listed in the clinical trial report associated with an appropriate flag.

Descriptive statistics of parameters will be calculated only when at least 2/3 of the individual parameter estimates of a certain parameter are available. If the actual sampling time will not be recorded or will be missing for a certain time point, the planned time will generally be used for this time point instead. Pharmacokinetic parameters which cannot be determined will be identified by "not calculated" (NC).

Noncompartmental pharmacokinetic parameters will be determined using the WinNonlin™ software program (Professional, version 4.1 or higher, Pharsight Corporation, Mountain View, California) or another validated program.

Individual $C_{max,ss}$, $t_{max,ss}$, and $C_{pre,ss}$ values will be directly determined from the plasma concentration time profiles of each patient. If the same $C_{max,ss}$ concentration occurs at different time points, $t_{max,ss}$ is assigned to the first occurrence of $C_{max,ss}$.

Estimation of $\lambda_{z,ss}$: The apparent terminal rate constant $\lambda_{z,ss}$ will be estimated from a regression of ln(C) versus time over the terminal log-linear disposition portion of the concentration-time profiles. At least three data points should be used in the calculation of $\lambda_{z,ss}$. In addition, the lower (tλz,start(ss)) and upper (tλz,end(ss)) limit on time for values to be included in the calculation of $\lambda_{z,ss}$ will be listed.

$t_{1/2,ss}$: The terminal half-life will be calculated from the terminal rate constant using the equation

$$t_{1/2(ss)} = \frac{\ln 2}{\lambda_{z(ss)}}$$

AUC: The area under the curve will be calculated using the linear up/log down algorithm. If an analyte concentration is equal to or higher than the preceding concentration, the linear trapezoidal method will be used. If the analyte concentration is smaller than the preceding concentration, the logarithmic method will be used.

Linear trapezoidal rule ($t_2 > t_1$ and $C_{t2} \geq C_{t1}$):

The area of the trapezoid between the two data points (t_1, C_{t1}) and (t_2, C_{t2}) will be computed by:

$$AUC_{t1\text{-}t2} = 0.5 \times (t_2 - t_1) \times (C_{t1} + C_{t2})$$

Logarithmic trapezoid rule ($t_2 > t_1$ and $C_{t2} < C_{t1}$):

The area of the trapezoid between the two data points (t_1, C_{t1}) and (t_2, C_{t2}) will be computed by:

$$AUC_{t1-t2} = \frac{(t_2 - t_1) \times (C_{t2} - C_{t1})}{\ln(C_{t2}/C_{t1})}$$

$MRT_{ih,ss}$: $MRT_{ih,ss}$ calculation in the steady state will be performed according to the following equation:

$$MRT_{po,ss} = \frac{AUMC_{ss}}{AUC_{\tau,ss}}$$

$AUMC_{ss}$ is the area under the first moment curve at steady state.

$CL/F_{,ss}$: The apparent clearance at steady state following extravascular multiple dose administration will be calculated as follows:

$$CL/F_{,ss} = \frac{Dose}{AUC_{\tau,ss}}$$

(F = absolute bioavailability factor)

$V_z/F_{,ss}$: The apparent volume of distribution during the terminal phase after inhalative administration will be determined according to the following equation:

$$V_z/F_{,ss} = \frac{CL/F_{,ss}}{\lambda_{z,ss}}$$

$fe_{t1\text{-}t2}$: The fraction excreted is calculated according to

$$fe_{t1-t2} = \frac{Ae_{t1-t2}}{D} * 100$$

where $Ae_{t1\text{-}t2}$ is the total quantity of the analyte that is excreted in urine over the time interval t1 to t2. This may represent the product of urine volume and urine analyte concentration for one time interval, as well as the cumulative amounts excreted calculated as the sum of the excreted amounts of subsequent time intervals. The **renal clearance** (**CL_R**) will be calculated as the quotient of the quantity of the analyte that is excreted in urine from the time point t1 until the time point t2 (Aet1-t2) and the area under the concentration-time curve within the same time interval (AUCt1-t2).

REV A

$$CL_{R,t1-t2} = \frac{Ae_{t1-t2}}{AUC_{t1-t2}}$$

gMean, gCV: The geometric mean (gMean) and coefficient of variation, gCV (given in %), will be calculated by the formulae:

$$\text{gMean} = \exp\left[\tfrac{1}{n}\sum_{i=1}^{n} \ln(x_i)\right] = \exp\left[\overline{\ln(x_i)}\right]$$

$$\text{gCV}(\%) = 100 \cdot \sqrt{\exp\left[\text{Var}(\ln(x_i))\right] - 1}$$

where

$$\text{Var}(\ln(x_i)) = \frac{1}{n-1}\sum_{i=1}^{n}\left[\ln(x_i) - \overline{\ln(x_i)}\right]^2$$

REV A

APPENDIX 10.6 CYSTIC FIBROSIS QUESTIONNAIRES

CFQ - R

Parents/Caregivers (Children Ages 6 to 13)

CYSTIC FIBROSIS QUESTIONNAIRE - REVISED

Understanding the impact of your child's illness and treatments on his or her everyday life can help your healthcare team keep track of your child's health and adjust his or her treatments. For this reason, we have developed a quality of life questionnaire specifically for parents of children with cystic fibrosis. We thank you for your willingness to complete this questionnaire.

Instructions: The following questions are about the current state of your child's health, as he or she perceives it. This information will allow us to better understand how he or she feels in everyday life.
Please answer all the questions. There are no right or wrong answers! If you are not sure how to answer, choose the response that seems closest to your child's situation.

Section I. Demographics

Please fill in the information or check the box indicating your answer.

A. What is your **child's** date of birth?

Date ☐☐|☐☐|☐☐☐☐
Mo Day Year

B. What is your relationship to the child?

- ☐ Mother
- ☐ Father
- ☐ Grandmother
- ☐ Grandfather
- ☐ Other relative
- ☐ Foster mother
- ☐ Foster father
- ☐ Other (please describe)

____

C. Which of the following best describes your child's racial or ethnic background?

- ☐ Caucasian
- ☐ African American
- ☐ Hispanic
- ☐ Asian/Oriental or Pacific Islander
- ☐ Native American or Native Alaskan
- ☐ Other (please describe)

____

- ☐ Prefer not to answer this question

D. During the **past two weeks**, has your child been on vacation or out of school for reasons **NOT** related to his or her health?

☐ Yes ☐ No

E. What is **your** date of birth?

Date ☐☐|☐☐|☐☐☐☐
Mo Day Year

F. What is your current marital status?

- ☐ Single/never married
- ☐ Married
- ☐ Widowed
- ☐ Divorced
- ☐ Separated
- ☐ Remarried
- ☐ With a partner

G. What is the highest grade in school you have completed?

- ☐ Some high school or less
- ☐ High school diploma/GED
- ☐ Vocational school
- ☐ Some college
- ☐ College degree
- ☐ Professional or graduate degree

H. Which of the following best describes you current work status?

- ☐ Seeking Work
- ☐ Working full or part time (either outside the home or at a home-based business)
- ☐ Full time homemaker
- ☐ Not working due to my health
- ☐ Not working for other reasons

Appendix 10.6 Part I (contdl)

CFQ - R

Parents/Caregivers (Children Ages 6 to 13)

CYSTIC FIBROSIS QUESTIONNAIRE - REVISED

Section II. Quality of Life

Please indicate how your child has been feeling during the past two weeks by checking the box matching your response.

To what extent has your child had difficulty:	A lot of difficulty	Some difficulty	A little difficulty	No difficulty
**1.** Performing vigorous activities such as running or playing sports	☐	☐	☐	☐
**2.** Walking as fast as others	☐	☐	☐	☐
**3.** Climbing stairs as fast as others	☐	☐	☐	☐
**4.** Carrying or lifting heavy objects such as books, a school bag, or backpack	☐	☐	☐	☐
**5.** Climbing several flights of stairs	☐	☐	☐	☐

Please check the box matching your response.

During the past two weeks, indicate how often your child:	Always	Often	Sometimes	Never
**6.** Seemed happy	☐	☐	☐	☐
**7.** Seemed worried	☐	☐	☐	☐
**8.** Seemed tired	☐	☐	☐	☐
**9.** Seemed short-tempered	☐	☐	☐	☐
**10.** Seemed well	☐	☐	☐	☐
**11.** Seemed grouchy	☐	☐	☐	☐
**12.** Seemed energetic	☐	☐	☐	☐
**13.** Was absent or late for school or other activities because of his/her illness or treatments	☐	☐	☐	☐

Please circle the number indicating your answer. Please choose only one answer for each question.

Thinking about the state of your child's health over the past two weeks, indicate:

14. The extent to which your child participated in sports and other physical activities, such as gym class
1. Has not participated in physical activities
2. Has participated less than usual in sports
3. Has participated as much as usual but with some difficulty
4. Has been able to participate in physical activities without any difficulty

15. The extent to which your child has difficulty walking
1. He or she can walk a long time without getting tired
2. He or she can walk a long time but gets tired
3. He or she cannot walk a long time, because he or she gets tired quickly
4. He or she avoids walking whenever possible, because it's too tiring for him or her

Please check the box that matches your response to these questions.

Appendix 10.6 Part I (contd.)

CFQ - R

Parents/Caregivers (Children Ages 6 to 13)

CYSTIC FIBROSIS QUESTIONNAIRE - REVISED

Please check the box that matches your response to these questions.

Thinking about your child's state of health during the past two weeks, indicate the extent to which each sentence is true or false for your child:

	Very true	Somewhat true	Somewhat false	Very false
**16.** My child has trouble recovering after physical effort........	☐	☐	☐	☐
**17.** Mealtimes are a struggle........	☐	☐	☐	☐
**18.** My child's treatments get in the way of his/her activities	☐	☐	☐	☐
**19.** My child feels small compared to other kids the same age	☐	☐	☐	☐
**20.** My child feels physically different from other kids the same age	☐	☐	☐	☐
**21.** My child thinks that he/she is too thin........	☐	☐	☐	☐
**22.** My child feels healthy	☐	☐	☐	☐
**23.** My child tends to be withdrawn	☐	☐	☐	☐
**24.** My child leads a normal life	☐	☐	☐	☐
**25.** My child has less fun than usual........	☐	☐	☐	☐
**26.** My child has trouble getting along with others	☐	☐	☐	☐
**27.** My child has trouble concentrating	☐	☐	☐	☐
**28.** My child is able to keep up with his/her school work or summer activities (e.g. camp)	☐	☐	☐	☐
**29.** My child is not doing as well as usual in school or summer activities (e.g. camp)....	☐	☐	☐	☐
**30.** My child spends a lot of time on his/her treatments everyday........	☐	☐	☐	☐

Please circle the number indicating your answer. Please choose only one answer for each question.

31. How difficult is it for your child to do his/her treatments (including medications) each day?
1. Not at all
2. A little
3. Moderately
4. Very

32. How do you think your child's health is now?
1. Excellent
2. Good
3. Fair
4. Poor

Appendix 10.6 Part I (contd.)

CFQ - R

Parents/Caregivers (Children Ages 6 to 13)

CYSTIC FIBROSIS QUESTIONNAIRE - REVISED

Section III. Symptom Difficulties

The next set of questions is designed to determine the frequency with which your child has certain respiratory difficulties, such as coughing or shortness of breath.

Please indicate how your child has been feeling during the past two weeks.	A great deal	Somewhat	A little	Not at all
33. My child had trouble gaining weight	☐	☐	☐	☐
34. My child was congested	☐	☐	☐	☐
35. My child coughed during the day	☐	☐	☐	☐
36. My child had to cough up mucus	☐	☐	☐	☐ → Go to Question 38

37. My child's mucus has been mostly: ☐ Clear ☐ Clear to yellow ☐ Yellowish-green ☐ Green with traces of blood ☐ Don't know

During the past two weeks:	Always	Often	Sometimes	Never
38. My child wheezed	☐	☐	☐	☐
39. My child had trouble breathing	☐	☐	☐	☐
40. My child woke up during the night because he/she was coughing	☐	☐	☐	☐
41. My child had gas	☐	☐	☐	☐
42. My child had diarrhea	☐	☐	☐	☐
43. My child had abdominal pain	☐	☐	☐	☐
44. My child has had eating problems	☐	☐	☐	☐

Please be sure you have answered all the questions.

THANK YOU FOR YOUR COOPERATION!

Appendix 10.6 Part II

CFQ-R

Children Ages 6 to 11 (Interviewer Format)

CYSTIC FIBROSIS QUESTIONNAIRE-REVISED

This questionnaire is formatted for use by an interviewer. Please use this format for younger children. For older children who seem able to read and answer the questions on their own, such as 12 and 13 year olds, use this questionnaire in its self-report format.

There are directions for the interviewer for each section of the questionnaire. Directions that you should *read* to the child are indicated by quotation marks. Directions that you are to *follow* are underlined and set in italics.

Interviewer: *Please ask the following questions.*

A. What is your date of birth?

Date ☐☐ ☐☐ ☐☐☐☐
Mo Day Year

B. Are you?
☐ Male ☐ Female

C. During the **past two weeks**, have you been on vacation or out of school for reasons **NOT** related to your health?

☐ Yes ☐ No

D. Which of the following best describes your racial background?
☐ Caucasian
☐ African American
☐ Hispanic
☐ Asian/Oriental or Pacific Islander
☐ Native American or Native Alaskan
☐ Other (please describe)
____
☐ Prefer not to answer this question

E. What grade are you in now?
(If summer, grade just finished)
☐ Kindergarten
☐ 1st grade
☐ 2nd grade
☐ 3rd grade
☐ 4th grade
☐ 5th grade
☐ 6th grade
☐ 7th grade
☐ Not in school

Appendix 10.6 Part II (contd.)

CFQ-R

Children Ages 6 to 11 (Interviewer Format)

CYSTIC FIBROSIS QUESTIONNAIRE-REVISED

Interviewer: *Please read the following to the child:*

"These questions are for children like you who have cystic fibrosis. Your answers will help us understand what this disease is like and how your treatments help you. So, answering these questions will help you and others like you in the future."

"For each question that I ask, choose one of the answers on the cards I'm about to show you."

Present the orange card to the child.

"Look at this card and read with me what it says: **very true, mostly true, somewhat true, not at all true**."

"Here's an example: If I asked you if it is **very true, mostly true, somewhat true, not at all true** that elephants can fly, which one of the four answers on the card would you choose?"

Present the blue card to the child.

"Now, look at this card and read with me what it says: **always** / **often** / **sometimes** / **never**."

"Here's another example: If I asked you if you go to the moon **always, often, sometimes**, or **never**, which answer on the card would you choose?"

Present the orange card to the child.

"Now, I will ask you some questions about your everyday life."

"Tell me if you find the statements I read to you to be **very true, mostly true, somewhat true, or not at all true**."

Please check the box indicating the child's response.

"During the past **two weeks**":	Very True	Mostly True	Somewhat True	Not at all True
**1.** You were able to walk as fast as others	☐	☐	☐	☐
**2.** You were able to climb stairs as fast as others	☐	☐	☐	☐
**3.** You were able to run, jump, and climb as you wanted	☐	☐	☐	☐
**4.** You were able to run as quickly and as long as others	☐	☐	☐	☐
**5.** You were able to participate in sports that you enjoy (e.g., swimming, soccer, dancing or others)	☐	☐	☐	☐
**6.** You had difficulty carrying or lifting heavy things such as books, your school bag, or a backpack	☐	☐	☐	☐

Appendix 10.6 Part II (contd.)

CFQ-R

Children Ages 6 to 11 (Interviewer Format)

CYSTIC FIBROSIS QUESTIONNAIRE-REVISED

Interviewer: *Present the blue card to the child.*

Please check the box indicating the child's response.

"And during these past **two weeks**, tell me how often":	Always	Often	Sometimes	Never
7. You felt tired	☐	☐	☐	☐
8. You felt mad	☐	☐	☐	☐
9. You felt grouchy	☐	☐	☐	☐
10. You felt worried	☐	☐	☐	☐
11. You felt sad	☐	☐	☐	☐
12. You had trouble falling asleep	☐	☐	☐	☐
13. You had bad dreams or nightmares	☐	☐	☐	☐
14. You felt good about yourself	☐	☐	☐	☐
15. You had trouble eating	☐	☐	☐	☐
16. You had to stop fun activities to do your treatments	☐	☐	☐	☐
17. You were pushed to eat	☐	☐	☐	☐

Interviewer: *Present the orange card to the child.*

"Now tell me if you find the statements I read to you to be **very true, mostly true, somewhat true**, or **not at all true**."

Please check the box indicating the child's response.

"During the past **two weeks**":	Very True	Mostly True	Somewhat True	Not at all True
18. You were able to do all of your treatments	☐	☐	☐	☐
19. You enjoyed eating	☐	☐	☐	☐
20. You got together with friends a lot	☐	☐	☐	☐
21. You stayed at home more than you wanted to	☐	☐	☐	☐
22. You felt comfortable sleeping away from home (at a friend or family member's house or elsewhere)	☐	☐	☐	☐
23. You felt left out	☐	☐	☐	☐

Appendix 10.6 Part II (contd.)

CFQ-R

Children Ages 6 to 11 (Interviewer Format)

CYSTIC FIBROSIS QUESTIONNAIRE-REVISED

"During the past **two weeks**":	Very True	Mostly True	Somewhat True	Not at all True
**24.** You often invited friends to your house	☐	☐	☐	☐
**25.** You were teased by other children	☐	☐	☐	☐
**26.** You felt comfortable discussing your illness with others (friends, teachers	☐	☐	☐	☐
**27.** You thought you were too short	☐	☐	☐	☐
**28.** You thought you were too thin	☐	☐	☐	☐
**29.** You thought you were physically different from others your age	☐	☐	☐	☐
**30.** Doing your treatments bothered you	☐	☐	☐	☐

Interviewer: ***Present the blue card to the child again***

Please check the box indicating the child's response.

"Tell me how often in the past **two weeks**":	Always	Often	Sometimes	Never
**31.** You coughed during the day	☐	☐	☐	☐
**32.** You woke up during the night because you were coughing	☐	☐	☐	☐
**33.** You had to cough up mucus	☐	☐	☐	☐
**34.** You had trouble breathing	☐	☐	☐	☐
**35.** Your stomach hurt	☐	☐	☐	☐

*Please be sure **all** the questions have been answered.*

THANK YOU FOR YOUR COOPERATION!

Appendix 10.6 Part III

CFQ-R

Children Ages 12 and 13 (Self-report Format)

CYSTIC FIBROSIS QUESTIONNAIRE-REVISED

These questions are for children like you who have cystic fibrosis. Your answers will help us understand what this disease is like and how your treatments help you. So, answering these questions will help you and others like you in the future.

Please answer all the questions. There are no right or wrong answers! If you are not sure how to answer, choose the response that seems closest to your situation.

Please fill in the answer or check the box that matches your response to these questions.

A. What is your date of birth?

Date ☐☐ ☐☐ ☐☐☐☐
Mo Day Year

B. Are you?

☐ Male ☐ Female

C. During the **past two weeks**, have you been on vacation or out of school for reasons **NOT** related to your health?

☐ Yes ☐ No

D. Which of the following best describes your racial background?

- ☐ Caucasian
- ☐ African American
- ☐ Hispanic
- ☐ Asian/Oriental or Pacific Islander
- ☐ Native American or Native Alaskan
- ☐ Other (please describe) ____________________
- ☐ Prefer not to answer this question

E. What grade are you in now?

(If summer, grade you just finished)

- ☐ 5th grade
- ☐ 6th grade
- ☐ 7th grade
- ☐ 8th grade
- ☐ 9th grade
- ☐ Not in school

©Quittner, Buu, Watrous and Davis, 2000. Revised 2002. CFQ-R — Child (Self-report Format), English Version 2.0

Appendix 10.6 Part III (contd.)

CFQ-R

Children Ages 12 and 13 (Self-report Format)

CYSTIC FIBROSIS QUESTIONNAIRE-REVISED

Please check the box matching your response.

In the past **two weeks**:	Very True	Mostly True	Somewhat True	Not at all True
1. You were able to walk as fast as others	☐	☐	☐	☐
2. You were able to climb stairs as fast as others	☐	☐	☐	☐
3. You were able to run, jump, and climb as you wanted	☐	☐	☐	☐
4. You were able to run as quickly and as long as others	☐	☐	☐	☐
5. You were able to participate in sports that you enjoy (e.g., swimming, soccer, dancing or others)	☐	☐	☐	☐
6. You had difficulty carrying or lifting heavy things such as books, your school bag, or a backpack	☐	☐	☐	☐

Please check the box matching your response.

And during these past **two weeks**, indicate how often:	Always	Often	Sometimes	Never
7. You felt tired	☐	☐	☐	☐
8. You felt mad	☐	☐	☐	☐
9. You felt grouchy	☐	☐	☐	☐
10. You felt worried	☐	☐	☐	☐
11. You felt sad	☐	☐	☐	☐
12. You had trouble falling asleep	☐	☐	☐	☐
13. You had bad dreams or nightmares	☐	☐	☐	☐
14. You felt good about yourself	☐	☐	☐	☐
15. You had trouble eating	☐	☐	☐	☐

©Quittner, Buu, Watrous and Davis, 2000. Revised 2002. CFQ-R — Child (Self-report Format), English Version 2.0

Appendix 10.6 Part III (contd.)

CFQ-R

Children Ages 12 and 13 (Self-report Format)

CYSTIC FIBROSIS QUESTIONNAIRE-REVISED

Please check the box matching your response.

And during these past **two weeks**, indicate how often:

	Always	Often	Sometimes	Never
**16.** You had to stop fun activities to do your treatments	☐	☐	☐	☐
**17.** You were pushed to eat	☐	☐	☐	☐

Please check the box matching your response.

During the past **two weeks**:	Very True	Mostly True	Somewhat True	Not at all True
**18.** You were able to do all of your treatments	☐	☐	☐	☐
**19.** You enjoyed eating	☐	☐	☐	☐
**20.** You got together with friends a lot	☐	☐	☐	☐
**21.** You stayed at home more than you wanted to	☐	☐	☐	☐
**22.** You felt comfortable sleeping away from home (at a friend or family member's house or elsewhere)	☐	☐	☐	☐
**23.** You felt left out	☐	☐	☐	☐
**24.** You often invited friends to your house	☐	☐	☐	☐
**25.** You were teased by other children	☐	☐	☐	☐
**26.** You felt comfortable discussing your illness with others (friends, teachers)	☐	☐	☐	☐
**27.** You thought you were too short	☐	☐	☐	☐
**28.** You thought you were too thin	☐	☐	☐	☐
**29.** You thought you were physically different from others your age	☐	☐	☐	☐
**30.** Doing your treatments bothered you	☐	☐	☐	☐

Appendix 10.6 Part III (contd.)

CFQ-R **Children Ages 12 and 13** (Self-report Format)

CYSTIC FIBROSIS QUESTIONNAIRE-REVISED

Please check the box matching your response.

Let us know how often in the past **two weeks**:	Always	Often	Sometimes	Never
**31.** You coughed during the day	☐	☐	☐	☐
**32.** You woke up during the night because you were coughing	☐	☐	☐	☐
**33.** You had to cough up mucus	☐	☐	☐	☐
**34.** You had trouble breathing	☐	☐	☐	☐
**35.** Your stomach hurt	☐	☐	☐	☐

Please be sure all the questions have been answered.

THANK YOU FOR YOUR COOPERATION!

Appendix 10.6 Part IV

CFQ-R **Adolescents and Adults (Patients 14 Years Old and Older)**

CYSTIC FIBROSIS QUESTIONNAIRE - REVISED

Understanding the impact of your illness and treatments on your everyday life can help your healthcare team keep track of your health and adjust your treatments. For this reason, this questionnaire was specifically developed for people who have cystic fibrosis. Thank you for your willingness to complete this form.

Instructions: The following questions are about the current state of your health, as you perceive it. This information will allow us to better understand how you feel in your everyday life.

Please answer all the questions. There are **no** right or wrong answers! If you are not sure how to answer, choose the response that seems closest to your situation.

Section I. Demographics

Please fill-in the information or check the box indicating your answer.

A. What is your date of birth?
Date ☐☐ ☐☐ ☐☐☐☐
Mo Day Year

B. What is your gender?
☐ Male ☐ Female

C. During the past **two weeks**, have you been on vacation or out of school or work for reasons **NOT** related to your health?

☐ Yes ☐ No

D. What is your current marital status?
- ☐ Single/never married
- ☐ Married
- ☐ Widowed
- ☐ Divorced
- ☐ Separated
- ☐ Remarried
- ☐ With a partner

E. Which of the following best describes your racial background?
- ☐ Caucasian
- ☐ African American
- ☐ Hispanic
- ☐ Asian/Oriental or Pacific Islander
- ☐ Native American or Native Alaskan
- ☐ Other (please describe) ____________________
- ☐ Prefer not to answer this question

F. What is the highest grade of school you have completed?
- ☐ Some high school or less
- ☐ High school diploma/GED
- ☐ Vocational school
- ☐ Some college
- ☐ College degree
- ☐ Professional or graduate degree

G. Which of the following best describes your current work or school status?
- ☐ Attending school outside the home
- ☐ Taking educational courses at home
- ☐ Seeking work
- ☐ Working full or part time (either outside the home or at a home-based business)
- ☐ Full time homemaker
- ☐ Not attending school or working due to my health
- ☐ Not working for other reasons

©2000, Quittner, Buu, Watrous and Davis. Revised 2002. CFQ-R Teen/Adult, English Version 2.0

Appendix 10.6 Part IV (contd.)

CFQ-R

Adolescents and Adults (Patients 14 Years Old and Older)

CYSTIC FIBROSIS QUESTIONNAIRE - REVISED

Section II. Quality of Life

Please check the box indicating your answer.

During the past two weeks, to what extent have you had difficulty:	A lot of difficulty	Some difficulty	A little difficulty	No difficulty
1. Performing vigorous activities such as running or playing sports	☐	☐	☐	☐
2. Walking as fast as others	☐	☐	☐	☐
3. Carrying or lifting heavy things such as books, groceries, or school bags	☐	☐	☐	☐
4. Climbing one flight of stairs	☐	☐	☐	☐
5. Climbing stairs as fast as others	☐	☐	☐	☐

During the past two weeks, indicate how often:	Always	Often	Sometimes	Never
6. You felt well	☐	☐	☐	☐
7. You felt worried	☐	☐	☐	☐
8. You felt useless	☐	☐	☐	☐
9. You felt tired	☐	☐	☐	☐
10. You felt energetic	☐	☐	☐	☐
11. You felt exhausted	☐	☐	☐	☐
12. You felt sad	☐	☐	☐	☐

Please circle the number indicating your answer. Please choose only one answer for each question.

Thinking about the state of your health over the last two weeks:

13. To what extent do you have difficulty walking?
1. You can walk a long time without getting tired
2. You can walk a long time but you get tired
3. You cannot walk a long time because you get tired quickly
4. You avoid walking whenever possible because it's too tiring for you

14. How do you feel about eating?
1. Just thinking about food makes you feel sick
2. You never enjoy eating
3. You are sometimes able to enjoy eating
4. You are always able to enjoy eating

15. To what extent do your treatments make your daily life more difficult?
1. Not at all
2. A little
3. Moderately
4. A lot

©2000, Quittner, Buu, Watrous and Davis. Revised 2002. CFQ-R Teen/Adult, English Version 2.0

Appendix 10.6 Part IV (contd.)

CFQ-R **Adolescents and Adults** (Patients 14 Years Old and Older)

CYSTIC FIBROSIS QUESTIONNAIRE - REVISED

16. How much time do you currently spend each day on your treatments?
    1. A lot
    2. Some
    3. A little
    4. Not very much
17. How difficult is it for you to do your treatments (including medications) each day?
    1. Not at all
    2. A little
    3. Moderately
    4. Very
18. How do you think your health is now?
    1. Excellent
    2. Good
    3. Fair
    4. Poor

Please select a box indicating your answer.

*Thinking about your health during the past **two weeks**, indicate the extent to which each sentence is true or false for you.*	Very true	Somewhat true	Somewhat false	Very false
19. I have trouble recovering after physical effort	☐	☐	☐	☐
20. I have to limit vigorous activities such as running or playing sports	☐	☐	☐	☐
21. I have to force myself to eat	☐	☐	☐	☐
22. I have to stay at home more than I want to	☐	☐	☐	☐
23. I feel comfortable discussing my illness with others	☐	☐	☐	☐
24. I think I am too thin	☐	☐	☐	☐
25. I think I look different from others my age	☐	☐	☐	☐
26. I feel bad about my physical appearance	☐	☐	☐	☐
27. People are afraid that I may be contagious	☐	☐	☐	☐
28. I get together with my friends a lot	☐	☐	☐	☐
29. I think my coughing bothers others	☐	☐	☐	☐
30. I feel comfortable going out at night	☐	☐	☐	☐
31. I often feel lonely	☐	☐	☐	☐
32. I feel healthy	☐	☐	☐	☐
33. It is difficult to make plans for the future (for example, going to college, getting married, advancing in a job, etc.)	☐	☐	☐	☐
34. I lead a normal life	☐	☐	☐	☐

Appendix 10.6 Part IV (contd.)

CFQ-R

Adolescents and Adults (Patients 14 Years Old and Older)

CYSTIC FIBROSIS QUESTIONNAIRE - REVISED

Section III. School, Work, or Daily Activities

Questions 35 through 38 are about school, work, or other daily tasks.

35. To what extent did you have trouble keeping up with your schoolwork, professional work, or other daily activities during the past two weeks?
    1. You have had no trouble keeping up
    2. You have managed to keep up but it's been difficult
    3. You have been behind
    4. You have not been able to do these activities at all

36. How often were you absent from school, work, or unable to complete daily activities during the last two weeks because of your illness or treatments?
    ☐ Always ☐ Often ☐ Sometimes ☐ Never

37. How often does CF get in the way of meeting your school, work, or personal goals
    ☐ Always ☐ Often ☐ Sometimes ☐ Never

38. How often does CF interfere with getting out of the house to run errands such as shopping or going to the bank?
    ☐ Always ☐ Often ☐ Sometimes ☐ Never

Section IV. Symptom Difficulties

Please select a box indicating your answer.

Indicate how you have been feeling during the past two weeks.	A great deal	Somewhat	A little	Not at all
39. Have you had trouble gaining weight?	☐	☐	☐	☐
40. Have you been congested?	☐	☐	☐	☐
41. Have you been coughing during the day?	☐	☐	☐	☐
42. Have you had to cough up mucus?	☐	☐	☐	☐ Go to Question 44

43. Has your mucus been mostly: ☐ Clear ☐ Clear to yellow ☐ Yellowish-green ☐ Green with traces of blood ☐ Don't know

How often during the past two weeks:	Always	Often	Sometimes	Never
44. Have you been wheezing?	☐	☐	☐	☐
45. Have you had trouble breathing?	☐	☐	☐	☐
46. Have you woken up during the night because you were coughing?	☐	☐	☐	☐
47. Have you had problems with gas?	☐	☐	☐	☐
48. Have you had diarrhea?	☐	☐	☐	☐
49. Have you had abdominal pain?	☐	☐	☐	☐
50. Have you had eating problems?	☐	☐	☐	☐

Please be sure you have answered all the questions.

THANK YOU FOR YOUR COOPERATION!

Cystic Fibrosis Foundation

©2000, Quittner, Buu, Watrous and Davis. Revised 2002. CFQ-R Teen/Adult, English Version 2.0

Boehringer Ingelheim

Clinical Trial Protocol Amendment

Doc. No.: U08-3220-01-AM2

**Amendment Number:**	2		
**Date:**	07 May 2009	X	To be implemented only after documented approval of the *IRB / IEC / Competent Authorities*
**EudraCT No.:**<br>**BI Trial No.:**	2008-001156-43<br>205.339	☐	To be implemented immediately in order to eliminate hazard – *IRB / IEC / Competent Authority* to be notified of change with request for approval
**Investigational Product:**	Tiotropium bromide	☐	Can be implemented without *IRB / IEC / Competent Authority* approval as changes involve logistical or administrative aspects only
**Title:**	A randomized, double-blind, placebo-controlled parallel group study to investigate the safety and efficacy of two doses of tiotropium bromide (2.5 µg and 5 µg) administered once daily via the Respimat® device for 12 weeks in patients with cystic fibrosis		
**Rationale for Amendment:**	(i) To characterize the study population based on their reversibility to albuterol (salbutamol), (ii) Provide greater flexibility in FEV_1 requirements between Visits 1 and 2, (iii) Provide time frame for PFT testing.		

Proprietary confidential information.
© 2009 Boehringer Ingelheim International GmbH or one or more of its affiliated companies. All rights reserved.
This document may not - in full or in part - be passed on, reproduced, published or otherwise used without prior written permission.

Change 1: Addition of reversibility testing

Text from original protocol: None

Added text:

Section 5.1 (under sub-heading Spirometry):

At the screening visit (Visit 1), following the completion of three acceptable pre-bronchodilator forced expiratory maneuvers, albuterol (salbutamol) will be administered to each patient in order to document the degree of reversibility. The results of the reversibility test will only be used to characterize the patients enrolled in the trial, i.e., it will **not** be used as a specific inclusion criterion. After a gentle and incomplete expiration, a dose of 100 µg of salbutamol (albuterol) is inhaled in one breath to TLC. The breath is then held for 5–10 s before the subject exhales. Four separate doses (total dose 400 µg) are delivered at approximately 30-s intervals (this dose ensures that the response is high on the albuterol dose–response curve). Three additional, acceptable post-bronchodilator forced expiratory maneuvers tests are recorded ≥10 min and up to 15 min later after the last dose of albuterol (salbutamol) is inhaled.

Section 6.2.1.1 Visit 1:

Patients will have 400 µg of albuterol (salbutamol) administered. PFTs will be conducted between 10 and 15 minutes post-albuterol (salbutamol) (see Section 5.1 for details).

Reason for Change 1:

CF experts suggested that characterizing the population in this study by their response to albuterol (salbutamol) could be helpful to understanding the response to tiotropium. And this information may prove useful for planning Phase III.

Change 2: Change in requirement for a ≤10% change in FEV_1 between Visits 1 and 2.

Text from original protocol: Inclusion criterion 5c, Section 3.3.1:

c. Pre-bronchodilator FEV_1 at Visit 2 must be within 10% of FEV_1 at Visit 1. If pre-bronchodilator FEV_1 at Visit 2 is not within 10% of FEV_1 at Visit 1, Visit 2 may be re-scheduled once within 7 days

Added text:

c. Pre-bronchodilator FEV_1 at Visit 2 must be within 15% of FEV_1 at Visit 1. If pre-bronchodilator FEV_1 at Visit 2 is not within 15% of FEV_1 at Visit 1, Visit 2 may be re-scheduled once within 7 days. Investigator should also ascertain that the patient is symptomatically stable as per inclusion criterion 5.

Reason for Change 2:

A 15% change in FEV_1 is within the normal range of variability for the CF population. This will give the sites greater flexibility for including patients.

Change 3: Provide a time framework for PFT and body plethysmography testing.

Text from original protocol: None

Added text:

To footnote number 9 in Flow Chart:

PFTs should be conducted within ±10 minutes of the specified time.

To last paragraph under Spirometry, Section 5.1:

PFTs should be conducted within ±10 minutes of the specified time.

To body plethysmography bullets in Sections 6.2.2.1 and 6.2.2.5:

(±10 minutes)

Reason for Change 3:

This provides the site with flexibility to have the patient perform PFTs and body plethysmography within a time framework so as not to incur a protocol violation.
