# Supplementary material for: Testing Two Different Doses of Tiotropium Respimat® in Cystic Fibrosis: Phase 2 Randomized Trial Results
Source: PLoS One. 2014 Sep 4;9(9):e106195. doi: 10.1371/journal.pone.0106195 (PMC4154718; doi:10.1371/journal.pone.0106195)
Supplement: Checklist S1 — CONSORT checklist. (DOC) [file pone.0106195.s003.doc]

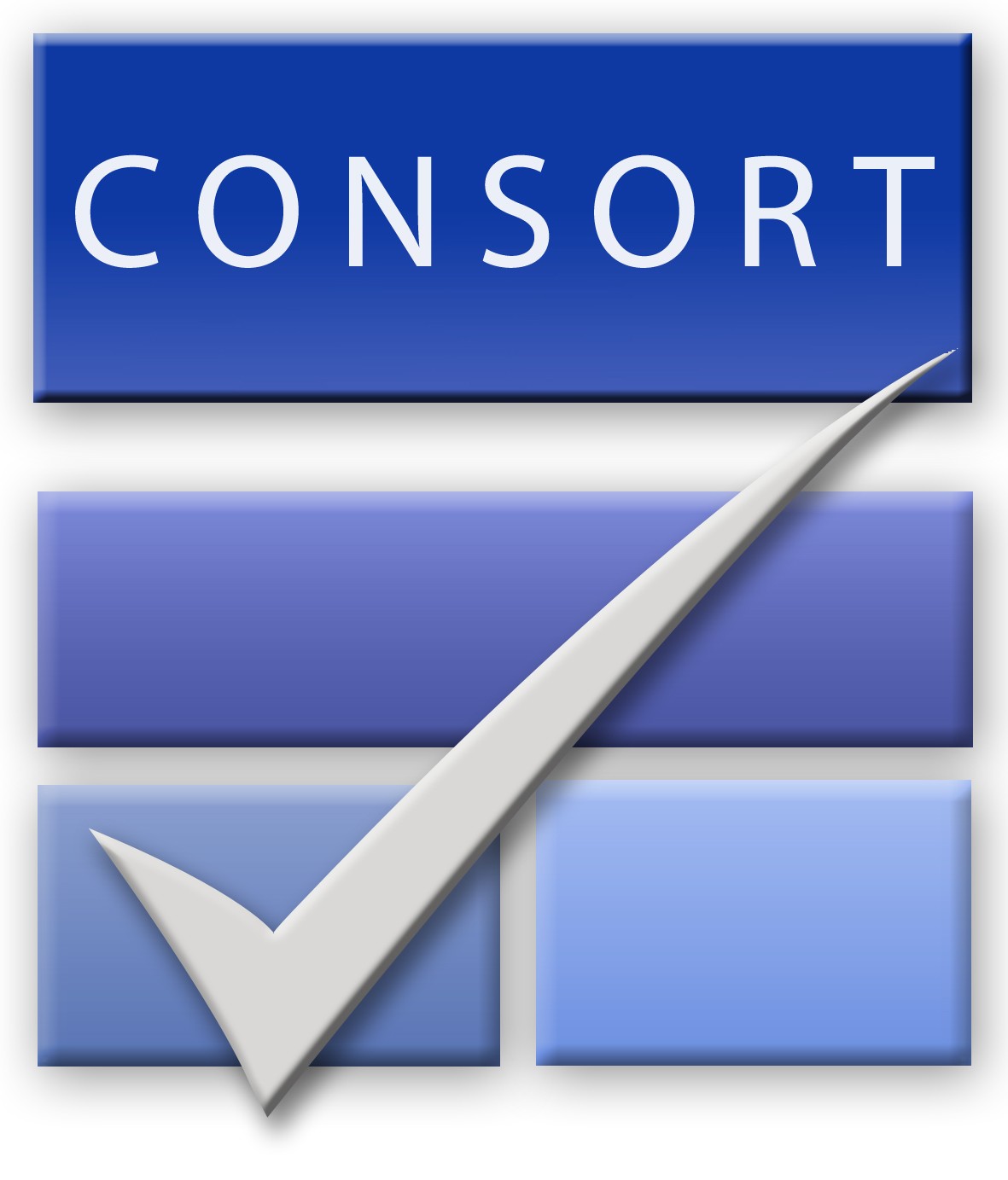
CONSORT 2010 checklist of information to include when reporting a randomised trial*

| Section/Topic | Item No | Checklist item | Reported in section: |
| --- | --- | --- | --- |
| Title and abstract | | | |
|  | 1a | Identification as a randomised trial in the title | Abstract |
|  | 1b | Structured summary of trial design, methods, results, and conclusions (for specific guidance see CONSORT for abstracts) | Abstract |
| Introduction | | | |
| Background and objectives | 2a | Scientific background and explanation of rationale | Methods (Study Design, Participants, End Points subsections |
|  | 2b | Specific objectives or hypotheses | Methods (End Points subsection) |
| Methods | | | |
| Trial design | 3a | Description of trial design (such as parallel, factorial) including allocation ratio | Methods (Study Design) and File S1 pages I-II |
|  | 3b | Important changes to methods after trial commencement (such as eligibility criteria), with reasons | File S1 page I |
| Participants | 4a | Eligibility criteria for participants | Methods (Participants subsection) |
|  | 4b | Settings and locations where the data were collected | Methods (Study Design subsection) |
| Interventions | 5 | The interventions for each group with sufficient details to allow replication, including how and when they were actually administered | Methods |
| Outcomes | 6a | Completely defined pre-specified primary and secondary outcome measures, including how and when they were assessed | Methods (End Points subsection) and File S1 pages I-IV |
|  | 6b | Any changes to trial outcomes after the trial commenced, with reasons | N/A |
| Sample size | 7a | How sample size was determined | File S1 pages I-II |
|  | 7b | When applicable, explanation of any interim analyses and stopping guidelines | N/A |
| Randomisation: |  |  |  |
| Sequence generation | 8a | Method used to generate the random allocation sequence | File S1 pages I-II |
|  | 8b | Type of randomisation; details of any restriction (such as blocking and block size) | File S1 pages I-II |
| Allocation concealment mechanism | 9 | Mechanism used to implement the random allocation sequence (such as sequentially numbered containers), describing any steps taken to conceal the sequence until interventions were assigned | File S1 pages I-II |
| Implementation | 10 | Who generated the random allocation sequence, who enrolled participants, and who assigned participants to interventions | File S1 page I |
| Blinding | 11a | If done, who was blinded after assignment to interventions (for example, participants, care providers, those assessing outcomes) and how | File S1 page I |
|  | 11b | If relevant, description of the similarity of interventions | Methods (Statistical Analyses subsection) |
| Statistical methods | 12a | Statistical methods used to compare groups for primary and secondary outcomes | Methods (Statistical Analyses subsection) |
|  | 12b | Methods for additional analyses, such as subgroup analyses and adjusted analyses | Methods (Statistical Analyses subsection) |
| Results | | | |
| Participant flow (a diagram is strongly recommended) | 13a | For each group, the numbers of participants who were randomly assigned, received intended treatment, and were analysed for the primary outcome | Figure 1 |
|  | 13b | For each group, losses and exclusions after randomisation, together with reasons | Figure 1 |
| Recruitment | 14a | Dates defining the periods of recruitment and follow-up | Methods (Study Design subsection) |
|  | 14b | Why the trial ended or was stopped | Methods (Study Design subsection) and File S1 pages I-II |
| Baseline data | 15 | A table showing baseline demographic and clinical characteristics for each group | Table 1 |
| Numbers analysed | 16 | For each group, number of participants (denominator) included in each analysis and whether the analysis was by original assigned groups | Results and File S1 pages VI-V |
| Outcomes and estimation | 17a | For each primary and secondary outcome, results for each group, and the estimated effect size and its precision (such as 95% confidence interval) | Results and File S1 pages VI-V |
|  | 17b | For binary outcomes, presentation of both absolute and relative effect sizes is recommended | N/A |
| Ancillary analyses | 18 | Results of any other analyses performed, including subgroup analyses and adjusted analyses, distinguishing pre-specified from exploratory | Results and File S1 pages I-II |
| Harms | 19 | All important harms or unintended effects in each group (for specific guidance see CONSORT for harms) | Results (Adverse Events) and File S1 page V |
| Discussion | | | |
| Limitations | 20 | Trial limitations, addressing sources of potential bias, imprecision, and, if relevant, multiplicity of analyses | Discussion |
| Generalisability | 21 | Generalisability (external validity, applicability) of the trial findings | Discussion |
| Interpretation | 22 | Interpretation consistent with results, balancing benefits and harms, and considering other relevant evidence | Discussion |
| Other information | | |  |
| Registration | 23 | Registration number and name of trial registry | Abstract |
| Protocol | 24 | Where the full trial protocol can be accessed, if available | N/A |
| Funding | 25 | Sources of funding and other support (such as supply of drugs), role of funders | Not included as per journal guidelines |

*We strongly recommend reading this statement in conjunction with the CONSORT 2010 Explanation and Elaboration for important clarifications on all the items. If relevant, we also recommend reading CONSORT extensions for cluster randomised trials, non-inferiority and equivalence trials, non-pharmacological treatments, herbal interventions, and pragmatic trials. Additional extensions are forthcoming: for those and for up to date references relevant to this checklist, see [www.consort-statement.org](http://www.consort-statement.org/).
